# Supplementary material for: A Spectroscopic Validation of the Improved Lennard–Jones Model
Source: Molecules. 2021 Jun 26;26(13):3906. doi: 10.3390/molecules26133906 (PMC8271504; doi:10.3390/molecules26133906)
Supplement: Supplementary file 1 [file molecules-26-03906-s001.zip › Supplementary_Material-1269185.pdf]

Supplementary Materials: A spectroscopic validation of the Improved Lennard-Jones model

Rhuiago Mendes de Oliveira[1], Luiz Guilherme Machado de Macedo[2], Thiago Ferreira da Cunha[3], Fernando Pirani[4], and Ricardo Gargano[5,*]

Table S1: He_2 electronic energies (in Hartree) calculated at CCSD(T)/aug-cc-pV4Z (CCSD(T)-4Z), CCSD(T)/aug-cc-pV5Z (CCSD(T)-5Z), and CCSD(T)/CBS levels.

R(Å)	CCSD(T)-4Z	SCF-4Z	CCSD(T)-5Z	SCF-5Z	CBS
2.00	-5.8033189597	-5.7207745338	-5.8046755184	-5.7209829996	-5.8059816783
2.10	-5.8040164346	-5.7215845688	-5.8053685586	-5.7217930053	-5.8066700821
2.20	-5.8044528490	-5.7221084119	-5.8058013855	-5.7223170000	-5.8070990601
2.30	-5.8047224658	-5.7224460207	-5.8060680255	-5.7226548959	-5.8073624156
2.40	-5.8048864130	-5.7226629979	-5.8062294864	-5.7228721004	-5.8075211402
2.50	-5.8049840425	-5.7228021569	-5.8063250417	-5.7230112554	-5.8076145214
2.60	-5.8050405004	-5.7228912296	-5.8063798134	-5.7231001078	-5.8076676478
2.70	-5.8050717326	-5.7229480610	-5.8064097098	-5.7231566864	-5.8076962848
2.80	-5.8050877661	-5.7229841159	-5.8064247135	-5.7231926571	-5.8077102554
2.90	-5.8050948472	-5.7230067994	-5.8064310192	-5.7232155063	-5.8077156544
2.91	-5.8050952267	-5.7230085348	-5.8064313328	-5.7232172705	-5.8077158827
2.92	-5.8050955586	-5.7230101899	-5.8064316007	-5.7232189562	-5.8077160662
2.93	-5.8050958465	-5.7230117681	-5.8064318268	-5.7232205666	-5.8077162094
2.94	-5.8050960926	-5.7230132730	-5.8064320131	-5.7232221053	-5.8077163140
2.95	-5.8050962995	-5.7230147078	-5.8064321619	-5.7232235752	-5.8077163822
2.96	-5.8050964694	-5.7230160756	-5.8064322756	-5.7232249794	-5.8077164164
2.97	-5.8050966046	-5.7230173796	-5.8064323563	-5.7232263208	-5.8077164189
2.98	-5.8050967071	-5.7230186226	-5.8064324060	-5.7232276021	-5.8077163916
2.99	-5.8050967790	-5.7230198074	-5.8064324267	-5.7232288260	-5.8077163367
3.00	-5.8050968222	-5.7230209367	-5.8064324202	-5.7232299949	-5.8077162559
3.01	-5.8050968384	-5.7230220129	-5.8064323884	-5.7232311112	-5.8077161511
3.02	-5.8050968294	-5.7230230386	-5.8064323328	-5.7232321772	-5.8077160240
3.03	-5.8050967967	-5.7230240161	-5.8064322550	-5.7232331952	-5.8077158762
3.04	-5.8050967420	-5.7230249476	-5.8064321566	-5.7232341671	-5.8077157093
3.05	-5.8050966667	-5.7230258353	-5.8064320390	-5.7232350951	-5.8077155246
3.06	-5.8050965721	-5.7230266812	-5.8064319035	-5.7232359810	-5.8077153237
3.07	-5.8050964597	-5.7230274874	-5.8064317514	-5.7232368266	-5.8077151077
3.08	-5.8050963305	-5.7230282555	-5.8064315839	-5.7232376338	-5.8077148781
3.09	-5.8050961859	-5.7230289876	-5.8064314021	-2.8616273298	-7.4153487627
3.10	-5.8050960268	-5.7230296852	-5.8064312076	-5.7232384041	-5.8077147962
3.20	-5.8050938545	-5.7230350966	-5.8064287330	-5.7232448612	-5.8077114169
3.30	-5.8050911162	-5.7230384757	-5.8064257761	-5.7232484011	-5.8077081403
3.40	-5.8050882652	-5.7230406257	-5.8064227672	-5.7232505679	-5.8077049563
3.50	-5.8050855423	-5.7230420235	-5.8064199288	-5.7232518886	-5.8077020402
3.60	-5.8050830607	-5.7230429441	-5.8064173623	-5.7232526998	-5.8076994459
3.70	-5.8050808630	-5.7230435461	-5.8064150996	-5.7232532093	-5.8076971669
3.80	-5.8050789506	-5.7230439257	-5.8064131368	-5.7232535397	-5.8076951790
3.90	-5.8050773043	-5.7230441473	-5.8064114510	-5.7232537597	-5.8076934527
4.00	-5.8050758966	-5.7230442583	-5.8064100120	-5.7232539071	-5.8076919603
4.10	-5.8050746973	-5.7230442959	-5.8064087876	-5.7232540031	-5.8076906767
4.20	-5.8050736773	-5.7230442887	-5.8064077474	-5.7232540611	-5.8076895787
4.30	-5.8050728099	-5.7230442579	-5.8064068637	-5.7232540908	-5.8076886440
4.40	-5.8050720716	-5.7230442178	-5.8064061123	-5.7232541003	-5.8076878510

4.50	-5.8050714421	-5.7230441774	-5.8064054723	-5.7232540964	-5.8076871795
4.60	-5.8050709044	-5.7230441412	-5.8064049261	-5.7232540846	-5.8076866106
4.70	-5.8050704439	-5.7230441110	-5.8064044603	-5.7232540691	-5.8076861309
4.80	-5.8050700484	-5.7230440868	-5.8064040578	-5.7232540527	-5.8076857167
4.90	-5.8050697080	-5.7230440679	-5.8064037128	-5.7232540372	-5.8076853651
5.00	-5.8050694140	-5.7230440531	-5.8064034152	-5.7232540235	-5.8076850631
5.10	-5.8050691595	-5.7230440415	-5.8064031577	-5.7232540117	-5.8076848026
5.20	-5.8050689385	-5.7230440322	-5.8064029343	-5.7232540020	-5.8076845769
5.30	-5.8050687461	-5.7230440246	-5.8064027400	-5.7232539941	-5.8076843806
5.40	-5.8050685782	-5.7230440183	-5.8064025711	-5.7232539876	-5.8076842107
5.50	-5.8050684313	-5.7230440129	-5.8064024227	-5.7232539823	-5.8076840608
5.60	-5.8050683023	-5.7230440084	-5.8064022920	-5.7232539779	-5.8076839284
5.70	-5.8050681890	-5.7230440047	-5.8064021777	-5.7232539743	-5.8076838130
5.80	-5.8050680890	-5.7230440015	-5.8064020773	-5.7232539712	-5.8076837120
5.90	-5.8050680007	-5.7230439990	-5.8064019880	-5.7232539687	-5.8076836217
6.00	-5.8050679225	-5.7230439970	-5.8064019093	-5.7232539666	-5.8076835424
6.10	-5.8050678531	-5.7230439954	-5.8064018394	-5.7232539648	-5.8076834721
6.20	-5.8050677914	-5.7230439942	-5.8064017773	-5.7232539634	-5.8076834098
6.30	-5.8050677365	-5.7230439932	-5.8064017220	-5.7232539622	-5.8076833542
6.40	-5.8050676874	-5.7230439926	-5.8064016727	-5.7232539613	-5.8076833048
6.50	-5.8050676435	-5.7230439921	-5.8064016286	-5.7232539605	-5.8076832606
6.60	-5.8050676042	-5.7230439918	-5.8064015891	-5.7232539599	-5.8076832210
6.70	-5.8050675690	-5.7230439916	-5.8064015536	-5.7232539595	-5.8076831855
6.80	-5.8050675372	-5.7230439915	-5.8064015217	-5.7232539591	-5.8076831536
6.90	-5.8050675087	-5.7230439914	-5.8064014931	-5.7232539589	-5.8076831249
7.00	-5.8050674829	-5.7230439914	-5.8064014672	-5.7232539587	-5.8076830990
7.10	-5.8050674597	-5.7230439914	-5.8064014438	-5.7232539586	-5.8076830756
7.20	-5.8050674386	-5.7230439914	-5.8064014227	-5.7232539585	-5.8076830545
7.30	-5.8050674196	-5.7230439913	-5.8064014036	-5.7232539584	-5.8076830353
7.40	-5.8050674023	-5.7230439913	-5.8064013863	-5.7232539584	-5.8076830179
7.50	-5.8050673866	-5.7230439913	-5.8064013705	-5.7232539583	-5.8076830021
7.60	-5.8050673723	-5.7230439913	-5.8064013562	-5.7232539583	-5.8076829877
7.70	-5.8050673593	-5.7230439913	-5.8064013431	-5.7232539583	-5.8076829746
7.80	-5.8050673474	-5.7230439913	-5.8064013312	-5.7232539583	-5.8076829627
7.90	-5.8050673366	-5.7230439913	-5.8064013204	-5.7232539583	-5.8076829518
8.00	-5.8050673267	-5.7230439913	-5.8064013104	-5.7232539583	-5.8076829418
8.10	-5.8050673177	-5.7230439913	-5.8064013014	-5.7232539583	-5.8076829327
8.20	-5.8050673094	-5.7230439913	-5.8064012930	-5.7232539583	-5.8076829244
8.30	-5.8050673017	-5.7230439913	-5.8064012854	-5.7232539583	-5.8076829167
8.40	-5.8050672948	-5.7230439913	-5.8064012784	-5.7232539583	-5.8076829097
8.50	-5.8050672883	-5.7230439913	-5.8064012720	-5.7232539583	-5.8076829033
8.60	-5.8050672824	-5.7230439913	-5.8064012660	-5.7232539583	-5.8076828973
8.70	-5.8050672770	-5.7230439913	-5.8064012606	-5.7232539583	-5.8076828919
8.80	-5.8050672720	-5.7230439913	-5.8064012556	-5.7232539583	-5.8076828868
8.90	-5.8050672673	-5.7230439913	-5.8064012509	-5.7232539583	-5.8076828822
9.00	-5.8050672631	-5.7230439913	-5.8064012467	-5.7232539583	-5.8076828779
9.10	-5.8050672591	-5.7230439913	-5.8064012427	-5.7232539583	-5.8076828739
9.20	-5.8050672555	-5.7230439913	-5.8064012390	-5.7232539583	-5.8076828703
9.30	-5.8050672521	-5.7230439913	-5.8064012356	-5.7232539583	-5.8076828669
9.40	-5.8050672489	-5.7230439913	-5.8064012325	-5.7232539583	-5.8076828637
9.50	-5.8050672460	-5.7230439913	-5.8064012296	-5.7232539583	-5.8076828608
9.60	-5.8050672433	-5.7230439913	-5.8064012269	-5.7232539583	-5.8076828581
9.70	-5.8050672408	-5.7230439913	-5.8064012244	-5.7232539583	-5.8076828556
9.80	-5.8050672385	-5.7230439913	-5.8064012220	-5.7232539583	-5.8076828533

9.90	-5.8050672363	-5.7230439913	-5.8064012199	-5.7232539583	-5.8076828511
10.00	-5.8050672343	-5.7230439913	-5.8064012178	-5.7232539583	-5.8076828490
10.10	-5.8050672324	-5.7230439913	-5.8064012160	-5.7232539583	-5.8076828471
10.20	-5.8050672306	-5.7230439913	-5.8064012142	-5.7232539583	-5.8076828454
10.30	-5.8050672290	-5.7230439913	-5.8064012126	-5.7232539583	-5.8076828438
10.40	-5.8050672275	-5.7230439913	-5.8064012110	-5.7232539583	-5.8076828422
10.50	-5.8050672260	-5.7230439913	-5.8064012096	-5.7232539583	-5.8076828408
10.60	-5.8050672247	-5.7230439913	-5.8064012083	-5.7232539583	-5.8076828395
10.70	-5.8050672234	-5.7230439913	-5.8064012070	-5.7232539583	-5.8076828382
10.80	-5.8050672223	-5.7230439913	-5.8064012058	-5.7232539583	-5.8076828370
10.90	-5.8050672212	-5.7230439913	-5.8064012047	-5.7232539583	-5.8076828359
11.00	-5.8050672202	-5.7230439913	-5.8064012037	-5.7232539583	-5.8076828349
11.10	-5.8050672192	-5.7230439913	-5.8064012027	-5.7232539583	-5.8076828339
11.20	-5.8050672183	-5.7230439913	-5.8064012018	-5.7232539583	-5.8076828331
11.30	-5.8050672174	-5.7230439913	-5.8064012010	-5.7232539583	-5.8076828322
11.40	-5.8050672166	-5.7230439913	-5.8064012002	-5.7232539583	-5.8076828314
11.50	-5.8050672159	-5.7230439913	-5.8064011994	-5.7232539583	-5.8076828306
11.60	-5.8050672152	-5.7230439913	-5.8064011987	-5.7232539583	-5.8076828299
11.70	-5.8050672145	-5.7230439913	-5.8064011981	-5.7232539583	-5.8076828293
11.80	-5.8050672139	-5.7230439913	-5.8064011974	-5.7232539583	-5.8076828286
11.90	-5.8050672133	-5.7230439913	-5.8064011968	-5.7232539583	-5.8076828281

Table S2: He-Ne electronic energies (in hartree) calculated at CCSD(T)/aug-cc-pV4Z (CCSD(T)-4Z), CCSD(T)/aug-cc-pV5Z (CCSD(T)-5Z), and CCSD(T)/CBS levels.

R(Å)	CCSD(T)-4Z	SCF-4Z	CCSD(T)-5Z	SCF-5Z	CBS
2.00	-131.7455729475	-131.3999545480	-131.7586952802	-131.4030546790	-131.7707213404
2.10	-131.7473055782	-131.4018769490	-131.7604120412	-131.4049800580	-131.7724197783
2.20	-131.7483937939	-131.4031139600	-131.7614870695	-131.4062185070	-131.7734801627
2.30	-131.7490705522	-131.4039081660	-131.7621529526	-131.4070133620	-131.7741342713
2.40	-131.7494861673	-131.4044162150	-131.7625597473	-131.4075224240	-131.7745312426
2.50	-131.7497372058	-131.4047391420	-131.7628037912	-131.4078476560	-131.7747666530
2.60	-131.7498854056	-131.4049425470	-131.7629465689	-131.4080548630	-131.7749016059
2.70	-131.7499700188	-131.4050694910	-131.7630270690	-131.4081865570	-131.7749751222
2.80	-131.7500158447	-131.4051483400	-131.7630698207	-131.4082701590	-131.7750119783
2.90	-131.7500384318	-131.4051975260	-131.7630901336	-131.4083232260	-131.7750277248
3.00	-131.7500474504	-131.4052286440	-131.7630974665	-131.4083568920	-131.7750318577
3.01	-131.7500478479	-131.4052310610	-131.7630977303	-131.4083594870	-131.7750318813
3.02	-131.7500481841	-131.4052333760	-131.7630979295	-131.4083619670	-131.7750318439
3.03	-131.7500484549	-131.4052355910	-131.7630980669	-131.4083643350	-131.7750317554
3.04	-131.7500486637	-131.4052377130	-131.7630981461	-131.4083665960	-131.7750316207
3.05	-131.7500488140	-131.4052397450	-131.7630981709	-131.4083687550	-131.7750314424
3.06	-131.7500489097	-131.4052416910	-131.7630981447	-131.4083708170	-131.7750312231
3.07	-131.7500489540	-131.4052435560	-131.7630980708	-131.4083727860	-131.7750309667
3.08	-131.7500489503	-131.4052453420	-131.7630979522	-131.4083746650	-131.7750306754
3.09	-131.7500489015	-131.4052470540	-131.7630977920	-131.4083764590	-131.7750303522
3.10	-131.7500488106	-131.4052486940	-131.7630975929	-131.4083781710	-131.7750299991
3.11	-131.7500486804	-131.4052502650	-131.7630973576	-131.4083798050	-131.7750296182
3.12	-131.7500485136	-131.4052517710	-131.7630970886	-131.4083813640	-131.7750292123
3.13	-131.7500483125	-131.4052532140	-131.7630967884	-131.4083828520	-131.7750287827
3.14	-131.7500480795	-131.4052545970	-131.7630964591	-131.4083842710	-131.7750283321
3.15	-131.7500478170	-131.4052559210	-131.7630961029	-131.4083856250	-131.7750278608
3.16	-131.7500475270	-131.4052571910	-131.7630957218	-131.4083869170	-131.7750273718
3.17	-131.7500472115	-131.4052584070	-131.7630953178	-131.4083881480	-131.7750268665

3.18	-131.7500468725	-131.4052595720	-131.7630948927	-131.4083893230	-131.7750263455
3.19	-131.7500465117	-131.4052606880	-131.7630944482	-131.4083904420	-131.7750258115
3.20	-131.7500461303	-131.4052617560	-131.7630939865	-131.4083915100	-131.7750252654
3.30	-131.7500415358	-131.4052701970	-131.7630886859	-131.4083997680	-131.7750193269
3.40	-131.7500362550	-131.4052754540	-131.7630828612	-131.4084048160	-131.7750130490
3.50	-131.7500309644	-131.4052784910	-131.7630771366	-131.4084078760	-131.7750068561
3.60	-131.7500260100	-131.4052800160	-131.7630718362	-131.4084097370	-131.7750010038
3.70	-131.7500215462	-131.4052805700	-131.7630670915	-131.4084108890	-131.7749956285
3.80	-131.7500176157	-131.4052805610	-131.7630629190	-131.4084116200	-131.7749907864
3.90	-131.7500142050	-131.4052802710	-131.7630593394	-131.4084120930	-131.7749866011
4.00	-131.7500112712	-131.4052798840	-131.7630562552	-131.4084123980	-131.7749829702
4.10	-131.7500087615	-131.4052795000	-131.7630536238	-131.4084125820	-131.7749798919
4.20	-131.7500066202	-131.4052791670	-131.7630513853	-131.4084126800	-131.7749773094
4.30	-131.7500047950	-131.4052788970	-131.7630494811	-131.4084127130	-131.7749751522
4.40	-131.7500032390	-131.4052786870	-131.7630478620	-131.4084127040	-131.7749733539
4.50	-131.7500019021	-131.4052785240	-131.7630464833	-131.4084126670	-131.7749718606
4.60	-131.7500007761	-131.4052783960	-131.7630453073	-131.4084126170	-131.7749705882
4.70	-131.7499998039	-131.4052782930	-131.7630443018	-131.4084125620	-131.7749695207
4.80	-131.7499989690	-131.4052782070	-131.7630434395	-131.4084125100	-131.7749686108
4.90	-131.7499982505	-131.4052781340	-131.7630426987	-131.4084124630	-131.7749678319
5.00	-131.7499976303	-131.4052780710	-131.7630420601	-131.4084124230	-131.7749671611
5.10	-131.7499970936	-131.4052780170	-131.7630415082	-131.4084123890	-131.7749665820
5.20	-131.7499966282	-131.4052779700	-131.7630410300	-131.4084123610	-131.7749660798
5.30	-131.7499962228	-131.4052779300	-131.7630406141	-131.4084123370	-131.7749656437
5.40	-131.7499958694	-131.4052778970	-131.7630402518	-131.4084123180	-131.7749652643
5.50	-131.7499955603	-131.4052778710	-131.7630399351	-131.4084123010	-131.7749649346
5.60	-131.7499952893	-131.4052778500	-131.7630396578	-131.4084122860	-131.7749646471
5.70	-131.7499950512	-131.4052778330	-131.7630394143	-131.4084122740	-131.7749643953
5.80	-131.7499948413	-131.4052778210	-131.7630391997	-131.4084122630	-131.7749641751
5.90	-131.7499946560	-131.4052778110	-131.7630390105	-131.4084122540	-131.7749639812
6.00	-131.7499944921	-131.4052778050	-131.7630388430	-131.4084122470	-131.7749638107
6.10	-131.7499943467	-131.4052778000	-131.7630386947	-131.4084122400	-131.7749636604
6.20	-131.7499942175	-131.4052777970	-131.7630385631	-131.4084122350	-131.7749635273
6.30	-131.7499941023	-131.4052777940	-131.7630384456	-131.4084122310	-131.7749634080
6.40	-131.7499940001	-131.4052777930	-131.7630383410	-131.4084122270	-131.7749633027
6.50	-131.7499939079	-131.4052777920	-131.7630382475	-131.4084122250	-131.7749632084
6.60	-131.7499938257	-131.4052777910	-131.7630381639	-131.4084122220	-131.7749631243
6.70	-131.7499937521	-131.4052777910	-131.7630380890	-131.4084122210	-131.7749630488
6.80	-131.7499936857	-131.4052777910	-131.7630380215	-131.4084122200	-131.7749629805
6.90	-131.7499936260	-131.4052777900	-131.7630379609	-131.4084122190	-131.7749629190
7.00	-131.7499935722	-131.4052777900	-131.7630379062	-131.4084122180	-131.7749628639
7.10	-131.7499935238	-131.4052777900	-131.7630378569	-131.4084122180	-131.7749628137
7.20	-131.7499934799	-131.4052777900	-131.7630378125	-131.4084122170	-131.7749627692
7.30	-131.7499934400	-131.4052777900	-131.7630377719	-131.4084122170	-131.7749627280
7.40	-131.7499934040	-131.4052777900	-131.7630377354	-131.4084122170	-131.7749626909
7.50	-131.7499933712	-131.4052777900	-131.7630377021	-131.4084122170	-131.7749626572
7.60	-131.7499933415	-131.4052777900	-131.7630376719	-131.4084122170	-131.7749626265
7.70	-131.7499933144	-131.4052777900	-131.7630376446	-131.4084122170	-131.7749625989
7.80	-131.7499932896	-131.4052777900	-131.7630376193	-131.4084122170	-131.7749625732
7.90	-131.7499932670	-131.4052777900	-131.7630375965	-131.4084122170	-131.7749625501
8.00	-131.7499932464	-131.4052777900	-131.7630375756	-131.4084122170	-131.7749625288
8.10	-131.7499932276	-131.4052777900	-131.7630375565	-131.4084122170	-131.7749625094
8.20	-131.7499932102	-131.4052777900	-131.7630375391	-131.4084122170	-131.7749624921
8.30	-131.7499931945	-131.4052777900	-131.7630375229	-131.4084122170	-131.7749624754

8.40	-131.7499931799	-131.4052777900	-131.7630375082	-131.4084122170	-131.7749624606
8.50	-131.7499931665	-131.4052777900	-131.7630374947	-131.4084122170	-131.7749624468
8.60	-131.7499931543	-131.4052777900	-131.7630374822	-131.4084122170	-131.7749624342
8.70	-131.7499931428	-131.4052777900	-131.7630374709	-131.4084122170	-131.7749624230
8.80	-131.7499931325	-131.4052777900	-131.7630374602	-131.4084122170	-131.7749624119
8.90	-131.7499931228	-131.4052777900	-131.7630374505	-131.4084122170	-131.7749624022
9.00	-131.7499931139	-131.4052777900	-131.7630374415	-131.4084122170	-131.7749623930
9.10	-131.7499931057	-131.4052777900	-131.7630374332	-131.4084122170	-131.7749623846
9.20	-131.7499930981	-131.4052777900	-131.7630374255	-131.4084122170	-131.7749623768
9.30	-131.7499930911	-131.4052777900	-131.7630374184	-131.4084122170	-131.7749623696
9.40	-131.7499930846	-131.4052777900	-131.7630374118	-131.4084122170	-131.7749623630
9.50	-131.7499930785	-131.4052777900	-131.7630374057	-131.4084122170	-131.7749623568
9.60	-131.7499930729	-131.4052777900	-131.7630374000	-131.4084122170	-131.7749623510
9.70	-131.7499930677	-131.4052777900	-131.7630373947	-131.4084122170	-131.7749623457
9.80	-131.7499930628	-131.4052777900	-131.7630373898	-131.4084122170	-131.7749623408
9.90	-131.7499930583	-131.4052777900	-131.7630373853	-131.4084122170	-131.7749623362
10.00	-131.7499930542	-131.4052777900	-131.7630373810	-131.4084122170	-131.7749623318
10.10	-131.7499930501	-131.4052777900	-131.7630373772	-131.4084122170	-131.7749623283
10.20	-131.7499930466	-131.4052777900	-131.7630373734	-131.4084122170	-131.7749623241
10.30	-131.7499930431	-131.4052777900	-131.7630373700	-131.4084122170	-131.7749623208
10.40	-131.7499930400	-131.4052777900	-131.7630373667	-131.4084122170	-131.7749623174
10.50	-131.7499930370	-131.4052777900	-131.7630373637	-131.4084122170	-131.7749623144
10.60	-131.7499930342	-131.4052777900	-131.7630373609	-131.4084122170	-131.7749623116
10.70	-131.7499930316	-131.4052777900	-131.7630373583	-131.4084122170	-131.7749623089
10.80	-131.7499930292	-131.4052777900	-131.7630373558	-131.4084122170	-131.7749623065
10.90	-131.7499930269	-131.4052777900	-131.7630373535	-131.4084122170	-131.7749623041
11.00	-131.7499930248	-131.4052777900	-131.7630373514	-131.4084122170	-131.7749623020
11.10	-131.7499930228	-131.4052777900	-131.7630373494	-131.4084122170	-131.7749622999
11.20	-131.7499930209	-131.4052777900	-131.7630373475	-131.4084122170	-131.7749622980
11.30	-131.7499930191	-131.4052777900	-131.7630373457	-131.4084122170	-131.7749622962
11.40	-131.7499930175	-131.4052777900	-131.7630373440	-131.4084122170	-131.7749622945
11.50	-131.7499930159	-131.4052777900	-131.7630373424	-131.4084122170	-131.7749622929
11.60	-131.7499930144	-131.4052777900	-131.7630373410	-131.4084122170	-131.7749622915
11.70	-131.7499930131	-131.4052777900	-131.7630373396	-131.4084122170	-131.7749622901
11.80	-131.7499930118	-131.4052777900	-131.7630373383	-131.4084122170	-131.7749622887
11.90	-131.7499930105	-131.4052777900	-131.7630373370	-131.4084122170	-131.7749622875

Table S3: He-Ar electronic energies (in hartree) calculated at CCSD(T)/aug-cc-pV4Z (CCSD(T)-4Z), CCSD(T)/aug-cc-pV5Z (CCSD(T)-5Z), and CCSD(T)/CBS levels.

R(Å)	CCSD(T)-4Z	SCF-4Z	CCSD(T)-5Z	SCF-5Z	CBS
2.00	-529.9549989866	-529.6520310050	-529.9642745230	-529.6526937420	-529.9736339121
2.10	-529.9623943541	-529.6601672940	-529.9716153726	-529.6608209870	-529.9809226434
2.20	-529.9674611891	-529.6658242490	-529.9766416682	-529.6664729420	-529.9859092149
2.30	-529.9709095666	-529.6697428880	-529.9800597727	-529.6703889950	-529.9892970103
2.40	-529.9732399704	-529.6724483660	-529.9823674431	-529.6730933250	-529.9915814741
2.50	-529.9748028136	-529.6743106990	-529.9839130943	-529.6749552670	-529.9931093075
2.60	-529.9758419312	-529.6755892150	-529.9849391005	-529.6762336650	-529.9941216237
2.70	-529.9765259992	-529.6764647740	-529.9856130653	-529.6771091280	-529.9947850425
2.80	-529.9769710072	-529.6770629820	-529.9860501844	-529.6777072330	-529.9952139425
2.90	-529.9772562513	-529.6774707940	-529.9863291896	-529.6781150060	-529.9954864239
3.00	-529.9774355963	-529.6777482570	-529.9865035523	-529.6783925180	-529.9956555318
3.10	-529.9775453958	-529.6779367190	-529.9866093509	-529.6785810700	-529.9957570821
3.20	-529.9776100307	-529.6780645450	-529.9866707583	-529.6787089650	-529.9958150645

3.30	-529.9776457258	-529.6781511060	-529.9867038506	-529.6787955690	-529.9958454019
3.40	-529.9776632046	-529.6782095850	-529.9867192333	-529.6788541230	-529.9958585433
3.41	-529.9776642470	-529.6782142620	-529.9867200895	-529.6788588120	-529.9958591974
3.42	-529.9776651835	-529.6782187580	-529.9867208447	-529.6788633210	-529.9958597549
3.43	-529.9776660208	-529.6782230780	-529.9867215045	-529.6788676560	-529.9958602202
3.44	-529.9776667637	-529.6782272310	-529.9867220739	-529.6788718230	-529.9958605996
3.45	-529.9776674171	-529.6782312210	-529.9867225575	-529.6788758300	-529.9958608956
3.46	-529.9776679854	-529.6782350560	-529.9867229599	-529.6788796830	-529.9958611137
3.47	-529.9776684731	-529.6782387410	-529.9867232853	-529.6788833870	-529.9958612581
3.48	-529.9776688844	-529.6782422820	-529.9867235377	-529.6788869480	-529.9958613328
3.49	-529.9776692231	-529.6782456840	-529.9867237212	-529.6788903720	-529.9958613411
3.50	-529.9776694932	-529.6782489530	-529.9867238395	-529.6788936630	-529.9958612878
3.51	-529.9776696982	-529.6782520930	-529.9867238962	-529.6788968280	-529.9958611747
3.52	-529.9776698418	-529.6782551100	-529.9867238946	-529.6788998700	-529.9958610069
3.53	-529.9776699271	-529.6782580080	-529.9867238381	-529.6789027950	-529.9958607864
3.54	-529.9776699574	-529.6782607920	-529.9867237298	-529.6789056070	-529.9958605168
3.55	-529.9776699358	-529.6782634660	-529.9867235726	-529.6789083100	-529.9958602011
3.56	-529.9776698651	-529.6782660340	-529.9867233694	-529.6789109090	-529.9958598415
3.57	-529.9776697483	-529.6782685010	-529.9867231230	-529.6789134070	-529.9958594418
3.58	-529.9776695878	-529.6782708700	-529.9867228360	-529.6789158080	-529.9958590040
3.59	-529.9776693863	-529.6782731450	-529.9867225107	-529.6789181170	-529.9958585298
3.60	-529.9776691450	-529.6782753290	-529.9867221490	-529.6789203360	-529.9958580220
3.70	-529.9776650804	-529.6782929080	-529.9867170164	-529.6789383040	-529.9958515504
3.80	-529.9776591377	-529.6783045740	-529.9867102295	-529.6789503780	-529.9958436486
3.90	-529.9776524472	-529.6783123020	-529.9867028713	-529.6789584580	-529.9958353921
4.00	-529.9776456813	-529.6783174360	-529.9866955776	-529.6789638340	-529.9958274087
4.10	-529.9776392186	-529.6783208720	-529.9866886989	-529.6789673880	-529.9958200272
4.20	-529.9776332565	-529.6783231950	-529.9866824042	-529.6789697260	-529.9958133751
4.30	-529.9776278743	-529.6783247800	-529.9866767584	-529.6789712630	-529.9958074798
4.40	-529.9776230871	-529.6783258630	-529.9866717601	-529.6789722790	-529.9958022976
4.50	-529.9776188707	-529.6783265940	-529.9866673735	-529.6789729600	-529.9957977606
4.60	-529.9776151814	-529.6783270720	-529.9866635453	-529.6789734250	-529.9957937938
4.70	-529.9776119653	-529.6783273650	-529.9866602231	-529.6789737470	-529.9957903442
4.80	-529.9776091693	-529.6783275230	-529.9866573312	-529.6789739710	-529.9957873145
4.90	-529.9776067409	-529.6783275850	-529.9866548294	-529.6789741240	-529.9957846845
5.00	-529.9776046320	-529.6783275830	-529.9866526608	-529.6789742240	-529.9957823959
5.10	-529.9776028002	-529.6783275410	-529.9866507799	-529.6789742840	-529.9957804063
5.20	-529.9776012058	-529.6783274770	-529.9866491467	-529.6789743130	-529.9957786800
5.30	-529.9775998176	-529.6783274030	-529.9866477263	-529.6789743210	-529.9957771798
5.40	-529.9775986062	-529.6783273280	-529.9866464887	-529.6789743130	-529.9957758771
5.50	-529.9775975470	-529.6783272580	-529.9866454082	-529.6789742950	-529.9957747452
5.60	-529.9775966205	-529.6783271940	-529.9866444629	-529.6789742720	-529.9957737571
5.70	-529.9775958055	-529.6783271370	-529.9866436343	-529.6789742480	-529.9957728956
5.80	-529.9775950896	-529.6783270880	-529.9866429061	-529.6789742220	-529.9957721417
5.90	-529.9775944585	-529.6783270460	-529.9866422649	-529.6789741990	-529.9957714791
6.00	-529.9775939011	-529.6783270090	-529.9866416989	-529.6789741770	-529.9957708957
6.10	-529.9775934085	-529.6783269780	-529.9866411982	-529.6789741570	-529.9957703803
6.20	-529.9775929700	-529.6783269510	-529.9866407547	-529.6789741390	-529.9957699264
6.30	-529.9775925810	-529.6783269280	-529.9866403606	-529.6789741240	-529.9957695225
6.40	-529.9775922346	-529.6783269080	-529.9866400099	-529.6789741100	-529.9957691638
6.50	-529.9775919256	-529.6783268910	-529.9866396971	-529.6789740980	-529.9957688444
6.60	-529.9775916501	-529.6783268770	-529.9866394176	-529.6789740880	-529.9957685584
6.70	-529.9775914019	-529.6783268650	-529.9866391676	-529.6789740790	-529.9957683049
6.80	-529.9775911799	-529.6783268550	-529.9866389433	-529.6789740710	-529.9957680771

6.90	-529.9775909804	-529.6783268470	-529.9866387419	-529.6789740640	-529.9957678730
7.00	-529.9775908009	-529.6783268400	-529.9866385606	-529.6789740580	-529.9957676894
7.10	-529.9775906398	-529.6783268350	-529.9866383972	-529.6789740530	-529.9957675235
7.20	-529.9775904929	-529.6783268310	-529.9866382498	-529.6789740490	-529.9957673757
7.30	-529.9775903607	-529.6783268280	-529.9866381165	-529.6789740450	-529.9957672418
7.40	-529.9775902409	-529.6783268250	-529.9866379959	-529.6789740420	-529.9957671201
7.50	-529.9775901323	-529.6783268230	-529.9866378864	-529.6789740400	-529.9957670098
7.60	-529.9775900344	-529.6783268220	-529.9866377870	-529.6789740380	-529.9957669093
7.70	-529.9775899439	-529.6783268210	-529.9866376967	-529.6789740370	-529.9957668193
7.80	-529.9775898622	-529.6783268200	-529.9866376145	-529.6789740350	-529.9957667371
7.90	-529.9775897877	-529.6783268190	-529.9866375395	-529.6789740340	-529.9957666616
8.00	-529.9775897197	-529.6783268190	-529.9866374711	-529.6789740340	-529.9957665928
8.10	-529.9775896583	-529.6783268190	-529.9866374085	-529.6789740330	-529.9957665294
8.20	-529.9775896007	-529.6783268190	-529.9866373514	-529.6789740330	-529.9957664728
8.30	-529.9775895485	-529.6783268180	-529.9866372990	-529.6789740320	-529.9957664202
8.40	-529.9775895007	-529.6783268180	-529.9866372509	-529.6789740320	-529.9957663719
8.50	-529.9775894568	-529.6783268180	-529.9866372068	-529.6789740320	-529.9957663275
8.60	-529.9775894173	-529.6783268180	-529.9866371662	-529.6789740320	-529.9957662858
8.70	-529.9775893794	-529.6783268180	-529.9866371290	-529.6789740320	-529.9957662493
8.80	-529.9775893452	-529.6783268180	-529.9866370946	-529.6789740320	-529.9957662148
8.90	-529.9775893137	-529.6783268180	-529.9866370630	-529.6789740320	-529.9957661831
9.00	-529.9775892846	-529.6783268180	-529.9866370338	-529.6789740320	-529.9957661537
9.10	-529.9775892577	-529.6783268180	-529.9866370068	-529.6789740310	-529.9957661272
9.20	-529.9775892329	-529.6783268180	-529.9866369819	-529.6789740310	-529.9957661022
9.30	-529.9775892099	-529.6783268180	-529.9866369588	-529.6789740310	-529.9957660791
9.40	-529.9775891886	-529.6783268180	-529.9866369375	-529.6789740310	-529.9957660576
9.50	-529.9775891689	-529.6783268180	-529.9866369177	-529.6789740310	-529.9957660378
9.60	-529.9775891506	-529.6783268180	-529.9866368993	-529.6789740310	-529.9957660194
9.70	-529.9775891336	-529.6783268180	-529.9866368823	-529.6789740310	-529.9957660023
9.80	-529.9775891178	-529.6783268180	-529.9866368664	-529.6789740310	-529.9957659864
9.90	-529.9775891031	-529.6783268180	-529.9866368517	-529.6789740310	-529.9957659716
10.00	-529.9775890894	-529.6783268180	-529.9866368380	-529.6789740310	-529.9957659579
10.10	-529.9775890767	-529.6783268180	-529.9866368253	-529.6789740310	-529.9957659451
10.20	-529.9775890648	-529.6783268180	-529.9866368134	-529.6789740310	-529.9957659332
10.30	-529.9775890538	-529.6783268180	-529.9866368023	-529.6789740310	-529.9957659221
10.40	-529.9775890435	-529.6783268180	-529.9866367920	-529.6789740310	-529.9957659117
10.50	-529.9775890338	-529.6783268180	-529.9866367823	-529.6789740310	-529.9957659020
10.60	-529.9775890248	-529.6783268180	-529.9866367733	-529.6789740310	-529.9957658930
10.70	-529.9775890164	-529.6783268180	-529.9866367648	-529.6789740310	-529.9957658846
10.80	-529.9775890085	-529.6783268180	-529.9866367569	-529.6789740310	-529.9957658766
10.90	-529.9775890011	-529.6783268180	-529.9866367495	-529.6789740310	-529.9957658692
11.00	-529.9775889942	-529.6783268180	-529.9866367426	-529.6789740310	-529.9957658623
11.10	-529.9775889877	-529.6783268180	-529.9866367361	-529.6789740310	-529.9957658558
11.20	-529.9775889816	-529.6783268180	-529.9866367300	-529.6789740310	-529.9957658497
11.30	-529.9775889759	-529.6783268180	-529.9866367243	-529.6789740310	-529.9957658440
11.40	-529.9775889705	-529.6783268180	-529.9866367189	-529.6789740310	-529.9957658386
11.50	-529.9775889655	-529.6783268180	-529.9866367139	-529.6789740310	-529.9957658335
11.60	-529.9775889608	-529.6783268180	-529.9866367092	-529.6789740310	-529.9957658288
11.70	-529.9775889563	-529.6783268180	-529.9866367047	-529.6789740310	-529.9957658243
11.80	-529.9775889521	-529.6783268180	-529.9866367005	-529.6789740310	-529.9957658201
11.90	-529.9775889482	-529.6783268180	-529.9866366965	-529.6789740310	-529.9957658162
12.00	-529.9775889444	-529.6783268180	-529.9866366928	-529.6789740310	-529.9957658125

Table S4: He-Kr electronic energies (in hartree) calculated at CCSD(T)/aug-cc-pV4Z (CCSD(T)-4Z), CCSD(T)/aug-cc-pV5Z (CCSD(T)-5Z), and CCSD(T)/CBS levels.

R(Å)	CCSD(T)-4Z	SCF-4Z	CCSD(T)-5Z	SCF-5Z	CBS
2.00	-2755.252990564	-2754.874497040	-2755.339920716	-2754.874785400	-2755.430964123
2.10	-2755.264150870	-2754.886635060	-2755.351002217	-2754.886921450	-2755.441964050
2.20	-2755.272029362	-2754.895312420	-2755.358816592	-2754.895597920	-2755.449711655
2.30	-2755.277557443	-2754.901490130	-2755.364292366	-2754.901775200	-2755.455132791
2.40	-2755.281412801	-2754.905872180	-2755.368105111	-2754.906156980	-2755.458900977
2.50	-2755.284084640	-2754.908970370	-2755.370742511	-2754.909254920	-2755.461502386
2.60	-2755.285923723	-2754.911154410	-2755.372554139	-2754.911438670	-2755.463285372
2.70	-2755.287180099	-2754.912689900	-2755.373788978	-2754.912973870	-2755.464497777
2.80	-2755.288031074	-2754.913766780	-2755.374623317	-2754.914050480	-2755.465314815
2.90	-2755.288601730	-2754.914520370	-2755.375181262	-2754.914803780	-2755.465859586
3.00	-2755.288979834	-2754.915046670	-2755.375549714	-2754.915329780	-2755.466218080
3.10	-2755.289226640	-2754.915413570	-2755.375789186	-2754.915696370	-2755.466450032
3.20	-2755.289384656	-2754.915668890	-2755.375941591	-2754.915951440	-2755.466596689
3.30	-2755.289483182	-2754.915846240	-2755.376035781	-2754.916128660	-2755.466686402
3.40	-2755.289542292	-2754.915969180	-2755.376091496	-2754.916251640	-2755.466738534
3.50	-2755.289575633	-2754.916054220	-2755.376122151	-2754.916336860	-2755.466766270
3.60	-2755.289592413	-2754.916112940	-2755.376136785	-2754.916395840	-2755.466778507
3.70	-2755.289598786	-2754.916153440	-2755.376141447	-2754.916436600	-2755.466781227
3.71	-2755.289599017	-2754.916156720	-2755.376141520	-2754.916439900	-2755.466781123
3.72	-2755.289599195	-2754.916159880	-2755.376141550	-2754.916443090	-2755.466780982
3.73	-2755.289599316	-2754.916162930	-2755.376141526	-2754.916446150	-2755.466780800
3.74	-2755.289599384	-2754.916165860	-2755.376141452	-2754.916449110	-2755.466780560
3.75	-2755.289599402	-2754.916168690	-2755.376141332	-2754.916451960	-2755.466780282
3.76	-2755.289599372	-2754.916171410	-2755.376141166	-2754.916454700	-2755.466779963
3.77	-2755.289599298	-2754.916174040	-2755.376140959	-2754.916457350	-2755.466779605
3.78	-2755.289599180	-2754.916176570	-2755.376140711	-2754.916459900	-2755.466779210
3.79	-2755.289599023	-2754.916179000	-2755.376140427	-2754.916462350	-2755.466778781
3.80	-2755.289598828	-2754.916181350	-2755.376140107	-2754.916464720	-2755.466778319
3.81	-2755.289598596	-2754.916183610	-2755.376139754	-2754.916466990	-2755.466777833
3.82	-2755.289598329	-2754.916185790	-2755.376139370	-2754.916469190	-2755.466777315
3.83	-2755.289598032	-2754.916187890	-2755.376138956	-2754.916471300	-2755.466776774
3.84	-2755.289597705	-2754.916189910	-2755.376138516	-2754.916473340	-2755.466776203
3.85	-2755.289597350	-2754.916191860	-2755.376138049	-2754.916475300	-2755.466775614
3.86	-2755.289596969	-2754.916193740	-2755.376137559	-2754.916477190	-2755.466775004
3.87	-2755.289596562	-2754.916195550	-2755.376137046	-2754.916479020	-2755.466774368
3.88	-2755.289596133	-2754.916197290	-2755.376136513	-2754.916480770	-2755.466773719
3.89	-2755.289595681	-2754.916198970	-2755.376135959	-2754.916482460	-2755.466773054
3.90	-2755.289595213	-2754.916200590	-2755.376135398	-2754.916484090	-2755.466772389
4.00	-2755.289589656	-2754.916213850	-2755.376128955	-2754.916497410	-2755.466764983
4.10	-2755.289583235	-2754.916222980	-2755.376121826	-2754.916506560	-2755.466757101
4.20	-2755.289576627	-2754.916229270	-2755.376114660	-2754.916512850	-2755.466749348
4.30	-2755.289570217	-2754.916233580	-2755.376107801	-2754.916517160	-2755.466742018
4.40	-2755.289564221	-2754.916236530	-2755.376101448	-2754.916520110	-2755.466735292
4.50	-2755.289558735	-2754.916238530	-2755.376095685	-2754.916522130	-2755.466729226
4.60	-2755.289553812	-2754.916239890	-2755.376090527	-2754.916523510	-2755.466723811
4.70	-2755.289549418	-2754.916240810	-2755.376085959	-2754.916524460	-2755.466719042
4.80	-2755.289545543	-2754.916241420	-2755.376081935	-2754.916525100	-2755.466714845
4.90	-2755.289542135	-2754.916241830	-2755.376078408	-2754.916525540	-2755.466711176
5.00	-2755.289539149	-2754.916242110	-2755.376075324	-2754.916525840	-2755.466707980
5.10	-2755.289536540	-2754.916242290	-2755.376072633	-2754.916526050	-2755.466705184
5.20	-2755.289534249	-2754.916242400	-2755.376070282	-2754.916526190	-2755.466702755

5.30	-2755.289532251	-2754.916242460	-2755.376068232	-2754.916526280	-2755.466700632
5.40	-2755.289530507	-2754.916242500	-2755.376066440	-2754.916526340	-2755.466698780
5.50	-2755.289528974	-2754.916242510	-2755.376064874	-2754.916526380	-2755.466697161
5.60	-2755.289527630	-2754.916242510	-2755.376063502	-2754.916526410	-2755.466695742
5.70	-2755.289526450	-2754.916242500	-2755.376062298	-2754.916526420	-2755.466694503
5.80	-2755.289525412	-2754.916242480	-2755.376061241	-2754.916526420	-2755.466693414
5.90	-2755.289524497	-2754.916242460	-2755.376060310	-2754.916526420	-2755.466692455
6.00	-2755.289523689	-2754.916242440	-2755.376059488	-2754.916526420	-2755.466691608
6.10	-2755.289522974	-2754.916242420	-2755.376058762	-2754.916526410	-2755.466690865
6.20	-2755.289522340	-2754.916242400	-2755.376058119	-2754.916526400	-2755.466690207
6.30	-2755.289521777	-2754.916242390	-2755.376057548	-2754.916526400	-2755.466689622
6.40	-2755.289521276	-2754.916242370	-2755.376057041	-2754.916526390	-2755.466689102
6.50	-2755.289520829	-2754.916242350	-2755.376056589	-2754.916526380	-2755.466688638
6.60	-2755.289520431	-2754.916242340	-2755.376056185	-2754.916526370	-2755.466688230
6.70	-2755.289520073	-2754.916242330	-2755.376055824	-2754.916526360	-2755.466687865
6.80	-2755.289519754	-2754.916242320	-2755.376055501	-2754.916526350	-2755.466687538
6.90	-2755.289519466	-2754.916242310	-2755.376055210	-2754.916526350	-2755.466687239
7.00	-2755.289519208	-2754.916242300	-2755.376054949	-2754.916526340	-2755.466686975
7.10	-2755.289518975	-2754.916242290	-2755.376054714	-2754.916526340	-2755.466686732
7.20	-2755.289518765	-2754.916242280	-2755.376054502	-2754.916526330	-2755.466686518
7.30	-2755.289518575	-2754.916242280	-2755.376054311	-2754.916526330	-2755.466686325
7.40	-2755.289518403	-2754.916242270	-2755.376054138	-2754.916526320	-2755.466686151
7.50	-2755.289518248	-2754.916242270	-2755.376053981	-2754.916526320	-2755.466685992
7.60	-2755.289518106	-2754.916242270	-2755.376053838	-2754.916526320	-2755.466685849
7.70	-2755.289517978	-2754.916242260	-2755.376053709	-2754.916526310	-2755.466685718
7.80	-2755.289517861	-2754.916242260	-2755.376053591	-2754.916526310	-2755.466685600
7.90	-2755.289517754	-2754.916242260	-2755.376053484	-2754.916526310	-2755.466685492
8.00	-2755.289517657	-2754.916242260	-2755.376053386	-2754.916526310	-2755.466685393
8.10	-2755.289517568	-2754.916242260	-2755.376053297	-2754.916526310	-2755.466685303
8.20	-2755.289517487	-2754.916242260	-2755.376053215	-2754.916526310	-2755.466685221
8.30	-2755.289517412	-2754.916242250	-2755.376053140	-2754.916526310	-2755.466685140
8.40	-2755.289517344	-2754.916242250	-2755.376053072	-2754.916526310	-2755.466685071
8.50	-2755.289517282	-2754.916242250	-2755.376053009	-2754.916526300	-2755.466685014
8.60	-2755.289517224	-2754.916242250	-2755.376052951	-2754.916526300	-2755.466684956
8.70	-2755.289517171	-2754.916242250	-2755.376052898	-2754.916526300	-2755.466684902
8.80	-2755.289517123	-2754.916242250	-2755.376052849	-2754.916526300	-2755.466684853
8.90	-2755.289517078	-2754.916242250	-2755.376052804	-2754.916526300	-2755.466684808
9.00	-2755.289517036	-2754.916242250	-2755.376052762	-2754.916526300	-2755.466684766
9.10	-2755.289516998	-2754.916242250	-2755.376052724	-2754.916526300	-2755.466684727
9.20	-2755.289516963	-2754.916242250	-2755.376052688	-2754.916526300	-2755.466684692
9.30	-2755.289516930	-2754.916242250	-2755.376052655	-2754.916526300	-2755.466684659
9.40	-2755.289516900	-2754.916242250	-2755.376052625	-2754.916526300	-2755.466684628
9.50	-2755.289516872	-2754.916242250	-2755.376052597	-2754.916526300	-2755.466684600
9.60	-2755.289516846	-2754.916242250	-2755.376052571	-2754.916526300	-2755.466684574
9.70	-2755.289516821	-2754.916242250	-2755.376052547	-2754.916526300	-2755.466684550
9.80	-2755.289516799	-2754.916242250	-2755.376052524	-2754.916526300	-2755.466684527
9.90	-2755.289516778	-2754.916242250	-2755.376052503	-2754.916526300	-2755.466684506
10.00	-2755.289516759	-2754.916242250	-2755.376052484	-2754.916526300	-2755.466684487
10.10	-2755.289516741	-2754.916242250	-2755.376052466	-2754.916526300	-2755.466684468
10.20	-2755.289516724	-2754.916242250	-2755.376052449	-2754.916526300	-2755.466684452
10.30	-2755.289516708	-2754.916242250	-2755.376052433	-2754.916526300	-2755.466684436
10.40	-2755.289516694	-2754.916242250	-2755.376052418	-2754.916526300	-2755.466684421
10.50	-2755.289516680	-2754.916242250	-2755.376052405	-2754.916526300	-2755.466684407
10.60	-2755.289516667	-2754.916242250	-2755.376052392	-2754.916526300	-2755.466684394

10.70	-2755.289516655	-2754.916242250	-2755.376052380	-2754.916526300	-2755.466684383
10.80	-2755.289516644	-2754.916242250	-2755.376052369	-2754.916526300	-2755.466684371
10.90	-2755.289516634	-2754.916242250	-2755.376052358	-2754.916526300	-2755.466684361
11.00	-2755.289516624	-2754.916242250	-2755.376052349	-2754.916526300	-2755.466684351
11.10	-2755.289516615	-2754.916242250	-2755.376052339	-2754.916526300	-2755.466684342
11.20	-2755.289516606	-2754.916242250	-2755.376052331	-2754.916526300	-2755.466684333
11.30	-2755.289516598	-2754.916242250	-2755.376052323	-2754.916526300	-2755.466684325
11.40	-2755.289516590	-2754.916242250	-2755.376052315	-2754.916526300	-2755.466684318
11.50	-2755.289516583	-2754.916242250	-2755.376052308	-2754.916526300	-2755.466684310
11.60	-2755.289516577	-2754.916242250	-2755.376052301	-2754.916526300	-2755.466684304
11.70	-2755.289516570	-2754.916242250	-2755.376052295	-2754.916526300	-2755.466684297
11.80	-2755.289516564	-2754.916242250	-2755.376052289	-2754.916526300	-2755.466684291
11.90	-2755.289516559	-2754.916242250	-2755.376052283	-2754.916526300	-2755.466684286
12.00	-2755.289516553	-2754.916242250	-2755.376052278	-2754.916526300	-2755.466684281

Table S5: He-Xe electronic energies (in hartree) calculated at CCSD(T)/aug-cc-pV4Z (CCSD(T)-4Z), CCSD(T)/aug-cc-pV5Z (CCSD(T)-5Z), and CCSD(T)/CBS levels.

R(Å)	CCSD(T)-4Z	SCF-4Z	CCSD(T)-5Z	SCF-5Z	CBS
2.00	-331.5173188458	-331.0926810160	-331.5937502896	-331.0928577950	-331.6738413436
2.10	-331.5343322241	-331.1108579400	-331.6106435185	-331.1110258780	-331.6906134809
2.20	-331.5467926710	-331.1243147760	-331.6230062665	-331.1244782510	-331.7028762324
2.30	-331.5558619375	-331.1342264650	-331.6319954535	-331.1343876490	-331.7117826887
2.40	-331.5624241177	-331.1414942530	-331.6384916951	-331.1416541920	-331.7182104482
2.50	-331.5671452511	-331.1468023620	-331.6431583699	-331.1469614980	-331.7228204373
2.60	-331.5705226807	-331.1506656840	-331.6464907404	-331.1508241220	-331.7261059248
2.70	-331.5729248513	-331.1534687640	-331.6488557056	-331.1536264420	-331.7284322819
2.80	-331.5746229090	-331.1554969310	-331.6505232721	-331.1556537450	-331.7300683429
2.90	-331.5758152328	-331.1569607170	-331.6516908679	-331.1571166080	-331.7312105131
3.00	-331.5766462069	-331.1580147350	-331.6525020526	-331.1581697260	-331.7320014408
3.10	-331.5772203778	-331.1580147350	-331.6530606312	-331.1589262690	-331.7321186394
3.20	-331.5776131609	-331.1587720750	-331.6534412584	-331.1594687260	-331.7326072326
3.30	-331.5778785918	-331.1593151740	-331.6536973289	-331.1598570180	-331.7329404519
3.40	-331.5780552423	-331.1597039290	-331.6538668222	-331.1601345350	-331.7331649288
3.50	-331.5781704831	-331.1599817290	-331.6539766004	-331.1603326010	-331.7333137698
3.60	-331.5782436416	-331.1601799150	-331.6540455597	-331.1604737770	-331.7334103512
3.70	-331.5782882364	-331.1603210730	-331.6540869196	-331.1605742730	-331.7334711607
3.80	-331.5783137117	-331.1604927230	-331.6541098483	-331.1606457200	-331.7335477110
3.90	-331.5783266058	-331.1605432620	-331.6541207390	-331.1606964500	-331.7335340754
3.91	-331.5783273920	-331.1605474240	-331.6541213505	-331.1607006300	-331.7335578360
3.92	-331.5783280989	-331.1605514440	-331.6541218850	-331.1607046680	-331.7335548420
3.93	-331.5783287329	-331.1605553280	-331.6541223507	-331.1607085700	-331.7335493653
3.94	-331.5783292973	-331.1605590800	-331.6541227504	-331.1607123400	-331.7335426515
3.95	-331.5783297952	-331.1605627050	-331.6541230870	-331.1607159820	-331.7335355181
3.96	-331.5783302296	-331.1605662070	-331.6541233636	-331.1607195010	-331.7335284077
3.97	-331.5783306036	-331.1605695910	-331.6541235833	-331.1607229010	-331.7335216382
3.98	-331.5783309199	-331.1605728590	-331.6541237487	-331.1607261860	-331.7335153566
3.99	-331.5783311812	-331.1605760170	-331.6541238624	-331.1607293600	-331.7335096302
4.00	-331.5783313902	-331.1605790680	-331.6541239416	-331.1607324260	-331.7335044723
4.01	-331.5783315495	-331.1605820150	-331.6541239595	-331.1607353880	-331.7334998685
4.02	-331.5783316612	-331.1605848620	-331.6541239331	-331.1607382490	-331.7334957864
4.03	-331.5783317277	-331.1605876130	-331.6541238643	-331.1607410130	-331.7334921753
4.04	-331.5783317514	-331.1605902700	-331.6541237480	-331.1607436840	-331.7334890026
4.05	-331.5783317344	-331.1605928370	-331.6541236022	-331.1607462640	-331.7334862131

4.06	-331.5783316786	-331.1605953170	-331.6541234189	-331.1607487560	-331.7334837625
4.07	-331.5783315860	-331.1605977130	-331.6541232026	-331.1607511640	-331.7334816109
4.08	-331.5783314586	-331.1606000270	-331.6541229535	-331.1607534900	-331.7334797192
4.09	-331.5783312981	-331.1606022630	-331.6541226740	-331.1607557360	-331.7334780505
4.10	-331.5783311057	-331.1606044230	-331.6541223672	-331.1607579060	-331.7334765857
4.20	-331.5783278020	-331.1606223770	-331.6541180231	-331.1607759330	-331.7334752918
4.30	-331.5783228352	-331.1606350910	-331.6541122103	-331.1607886710	-331.7334741484
4.40	-331.5783170857	-331.1606440950	-331.6541057821	-331.1607976630	-331.7334731353
4.50	-331.5783111177	-331.1606504660	-331.6540992496	-331.1608040080	-331.7334722369
4.60	-331.5783052628	-331.1606549680	-331.6540929410	-331.1608084830	-331.7334714391
4.70	-331.5782997228	-331.1606581410	-331.6540870346	-331.1608116400	-331.7334707288
4.80	-331.5782946014	-331.1606603700	-331.6540816177	-331.1608138680	-331.7334700947
4.90	-331.5782899404	-331.1606619290	-331.6540767183	-331.1608154420	-331.7334695296
5.00	-331.5782857442	-331.1606630160	-331.6540723298	-331.1608165530	-331.7334690227
5.10	-331.5782819950	-331.1606637720	-331.6540684258	-331.1608173370	-331.7334685691
5.20	-331.5782786641	-331.1606642950	-331.6540649664	-331.1608178900	-331.7334681616
5.30	-331.5782757143	-331.1606646580	-331.6540619147	-331.1608182780	-331.7334677953
5.40	-331.5782731077	-331.1606649080	-331.6540592249	-331.1608185500	-331.7334674651
5.50	-331.5782708073	-331.1606650790	-331.6540568561	-331.1608187390	-331.7334671680
5.60	-331.5782687779	-331.1606651960	-331.6540547706	-331.1608188690	-331.7334668993
5.70	-331.5782669875	-331.1606652730	-331.6540529341	-331.1608189580	-331.7334666566
5.80	-331.5782654110	-331.1606653230	-331.6540513157	-331.1608190190	-331.7334664364
5.90	-331.5782640139	-331.1606653530	-331.6540498880	-331.1608190580	-331.7334662369
6.00	-331.5782627788	-331.1606653680	-331.6540486269	-331.1608190830	-331.7334660561
6.10	-331.5782616849	-331.1606653730	-331.6540475116	-331.1608190980	-331.7334658914
6.20	-331.5782607148	-331.1606653700	-331.6540465236	-331.1608191060	-331.7334657418
6.30	-331.5782598531	-331.1606653630	-331.6540456470	-331.1608191100	-331.7334656053
6.40	-331.5782590865	-331.1606653530	-331.6540448679	-331.1608191100	-331.7334654806
6.50	-331.5782584034	-331.1606653410	-331.6540441742	-331.1608191080	-331.7334653675
6.60	-331.5782577937	-331.1606653280	-331.6540435556	-331.1608191060	-331.7334652629
6.70	-331.5782572485	-331.1606653160	-331.6540430029	-331.1608191020	-331.7334651676
6.80	-331.5782567602	-331.1606653030	-331.6540425083	-331.1608190990	-331.7334650805
6.90	-331.5782563222	-331.1606652920	-331.6540420649	-331.1608190960	-331.7334650002
7.00	-331.5782559287	-331.1606652810	-331.6540416667	-331.1608190930	-331.7334649263
7.10	-331.5782555745	-331.1606652710	-331.6540413085	-331.1608190900	-331.7334648587
7.20	-331.5782552552	-331.1606652620	-331.6540409858	-331.1608190880	-331.7334647960
7.30	-331.5782549669	-331.1606652550	-331.6540406946	-331.1608190860	-331.7334647386
7.40	-331.5782547063	-331.1606652480	-331.6540404314	-331.1608190840	-331.7334646846
7.50	-331.5782544703	-331.1606652420	-331.6540401932	-331.1608190820	-331.7334646351
7.60	-331.5782542563	-331.1606652360	-331.6540399773	-331.1608190800	-331.7334645900
7.70	-331.5782540620	-331.1606652320	-331.6540397813	-331.1608190790	-331.7334645477
7.80	-331.5782538853	-331.1606652280	-331.6540396031	-331.1608190770	-331.7334645084
7.90	-331.5782537244	-331.1606652240	-331.6540394410	-331.1608190750	-331.7334644721
8.00	-331.5782535777	-331.1606652220	-331.6540392931	-331.1608190740	-331.7334644388
8.10	-331.5782534438	-331.1606652190	-331.6540391582	-331.1608190720	-331.7334644075
8.20	-331.5782533214	-331.1606652170	-331.6540390350	-331.1608190710	-331.7334643783
8.30	-331.5782532095	-331.1606652160	-331.6540389222	-331.1608190690	-331.7334643512
8.40	-331.5782531070	-331.1606652140	-331.6540388189	-331.1608190680	-331.7334643259
8.50	-331.5782530129	-331.1606652130	-331.6540387242	-331.1608190670	-331.7334643023
8.60	-331.5782529265	-331.1606652130	-331.6540386373	-331.1608190660	-331.7334642804
8.70	-331.5782528471	-331.1606652120	-331.6540385574	-331.1608190650	-331.7334642599
8.80	-331.5782527740	-331.1606652110	-331.6540384839	-331.1608190640	-331.7334642407
8.90	-331.5782527068	-331.1606652110	-331.6540384163	-331.1608190630	-331.7334642229
9.00	-331.5782526448	-331.1606652110	-331.6540383539	-331.1608190630	-331.7334642061

9.10	-331.5782525876	-331.1606652110	-331.6540382963	-331.1608190620	-331.7334641905
9.20	-331.5782525347	-331.1606652100	-331.6540382432	-331.1608190620	-331.7334641758
9.30	-331.5782524858	-331.1606652100	-331.6540381940	-331.1608190620	-331.7334641621
9.40	-331.5782524406	-331.1606652100	-331.6540381486	-331.1608190610	-331.7334641492
9.50	-331.5782523987	-331.1606652100	-331.6540381065	-331.1608190610	-331.7334641372
9.60	-331.5782523599	-331.1606652100	-331.6540380674	-331.1608190610	-331.7334641258
9.70	-331.5782523239	-331.1606652100	-331.6540380313	-331.1608190610	-331.7334641152
9.80	-331.5782522905	-331.1606652100	-331.6540379977	-331.1608190600	-331.7334641052
9.90	-331.5782522594	-331.1606652100	-331.6540379664	-331.1608190600	-331.7334640958
10.00	-331.5782522305	-331.1606652100	-331.6540379374	-331.1608190600	-331.7334640870
10.10	-331.5782522036	-331.1606652100	-331.6540379104	-331.1608190600	-331.7334640787
10.20	-331.5782521786	-331.1606652100	-331.6540378852	-331.1608190600	-331.7334640708

Table S6: He-Rn electronic energies (in hartree) calculated at CCSD(T)/aug-cc-pV4Z (CCSD(T)-4Z), CCSD(T)/aug-cc-pV5Z (CCSD(T)-5Z), and CCSD(T)/CBS levels.

R(Å)	CCSD(T)-4Z	SCF-4Z	CCSD(T)-5Z	SCF-5Z	CBS
3.00	-290.8478632322	-290.4015535240	-290.9433623324	-290.4017131920	-291.0434684093
3.10	-290.8486121173	-290.4025097060	-290.9440902255	-290.4026684060	-291.0441748217
3.20	-290.8491356538	-290.4032047400	-290.9445970548	-290.4033626150	-291.0446645857
3.30	-290.8494981805	-290.4037091100	-290.9449464576	-290.4038663420	-291.0450005804
3.40	-290.8497463281	-290.4040745400	-290.9451844069	-290.4042313300	-291.0452280781
3.50	-290.8499137425	-290.4043389030	-290.9453439816	-290.4044954450	-291.0453795670
3.60	-290.8500245850	-290.4045298690	-290.9454488312	-290.4046863330	-291.0454781728
3.70	-290.8500961258	-290.4046676200	-290.9455157908	-290.4048241350	-291.0455402972
3.80	-290.8501406268	-290.4047668510	-290.9455567762	-290.4049234960	-291.0455775210
3.90	-290.8501667490	-290.4048382450	-290.9455801766	-290.4049950540	-291.0455979736
4.00	-290.8501805605	-290.4048895570	-290.9455918668	-290.4050465280	-291.0456073472
4.10	-290.8501863140	-290.4049264060	-290.9455959208	-290.4050835120	-291.0456095422
4.11	-290.8501865677	-290.4049294620	-290.9455960231	-290.4050865800	-291.0456094790
4.12	-290.8501867714	-290.4049324190	-290.9455960802	-290.4050895480	-291.0456093761
4.13	-290.8501869287	-290.4049352800	-290.9455960943	-290.4050924190	-291.0456092343
4.14	-290.8501870418	-290.4049380470	-290.9455960672	-290.4050951960	-291.0456090546
4.15	-290.8501871129	-290.4049407240	-290.9455959909	-290.4050978830	-291.0456088180
4.16	-290.8501871440	-290.4049433130	-290.9455958894	-290.4051004820	-291.0456085717
4.17	-290.8501871371	-290.4049458180	-290.9455957512	-290.4051029950	-291.0456082913
4.18	-290.8501870939	-290.4049482410	-290.9455955797	-290.4051054270	-291.0456079800
4.19	-290.8501870163	-290.4049505850	-290.9455953764	-290.4051077790	-291.0456076404
4.20	-290.8501869060	-290.4049528530	-290.9455951430	-290.4051100540	-291.0456072740
4.21	-290.8501867646	-290.4049550460	-290.9455948811	-290.4051122550	-291.0456068811
4.22	-290.8501865937	-290.4049571680	-290.9455945920	-290.4051143830	-291.0456064647
4.23	-290.8501863947	-290.4049592200	-290.9455942775	-290.4051164420	-291.0456060250
4.24	-290.8501861692	-290.4049612050	-290.9455939387	-290.4051184340	-291.0456055635
4.25	-290.8501859186	-290.4049631260	-290.9455935772	-290.4051203600	-291.0456050827
4.30	-290.8501843340	-290.4049718270	-290.9455914780	-290.4051290830	-291.0456024313
4.40	-290.8501799392	-290.4049854380	-290.9455861895	-290.4051427100	-291.0455961960
4.50	-290.8501746240	-290.4049952000	-290.9455801348	-290.4051524600	-291.0455893723
4.60	-290.8501689588	-290.4050021970	-290.9455738709	-290.4051594320	-291.0455824942
4.70	-290.8501633140	-290.4050072090	-290.9455677184	-290.4051644140	-291.0455758261
4.80	-290.8501578939	-290.4050107950	-290.9455618885	-290.4051679750	-291.0455695802
4.90	-290.8501528281	-290.4050133540	-290.9455564870	-290.4051705220	-291.0455638332
5.00	-290.8501481749	-290.4050151750	-290.9455515595	-290.4051723440	-291.0455586174
5.10	-290.8501439514	-290.4050164670	-290.9455471295	-290.4051736470	-291.0455539646
5.20	-290.8501401516	-290.4050173790	-290.9455431427	-290.4051745790	-291.0455497702

5.30	-290.8501367521	-290.4050180210	-290.9455395978	-290.4051752450	-291.0455460594
5.40	-290.8501337237	-290.4050184700	-290.9455364505	-290.4051757190	-291.0455427733
5.50	-290.8501310337	-290.4050187820	-290.9455336638	-290.4051760550	-291.0455398716
5.60	-290.8501286504	-290.4050189980	-290.9455311993	-290.4051762930	-291.0455373095
5.70	-290.8501265374	-290.4050191470	-290.9455290213	-290.4051764600	-291.0455350533
5.80	-290.8501246662	-290.4050192480	-290.9455270967	-290.4051765760	-291.0455330641
5.90	-290.8501230083	-290.4050193160	-290.9455253950	-290.4051766560	-291.0455313098
6.00	-290.8501215388	-290.4050193610	-290.9455238895	-290.4051767110	-291.0455297609
6.10	-290.8501202351	-290.4050193890	-290.9455225561	-290.4051767480	-291.0455283913
6.20	-290.8501190773	-290.4050194060	-290.9455213737	-290.4051767710	-291.0455271797
6.30	-290.8501180478	-290.4050194140	-290.9455203238	-290.4051767860	-291.0455261045
6.40	-290.8501171311	-290.4050194170	-290.9455193900	-290.4051767940	-291.0455251500
6.50	-290.8501163138	-290.4050194170	-290.9455185584	-290.4051767980	-291.0455243011
6.60	-290.8501155839	-290.4050194130	-290.9455178165	-290.4051767990	-291.0455235438
6.70	-290.8501149311	-290.4050194080	-290.9455171536	-290.4051767980	-291.0455228680
6.80	-290.8501143464	-290.4050194020	-290.9455165603	-290.4051767960	-291.0455222635
6.90	-290.8501138218	-290.4050193950	-290.9455160284	-290.4051767940	-291.0455217211
7.00	-290.8501133504	-290.4050193880	-290.9455155508	-290.4051767910	-291.0455212347
7.10	-290.8501129262	-290.4050193800	-290.9455151211	-290.4051767880	-291.0455207966
7.20	-290.8501125438	-290.4050193740	-290.9455147342	-290.4051767850	-291.0455204032
7.30	-290.8501121986	-290.4050193670	-290.9455143852	-290.4051767820	-291.0455200478
7.40	-290.8501118866	-290.4050193610	-290.9455140697	-290.4051767790	-291.0455197272
7.50	-290.8501116040	-290.4050193550	-290.9455137843	-290.4051767760	-291.0455194369
7.60	-290.8501113479	-290.4050193500	-290.9455135256	-290.4051767740	-291.0455191740
7.70	-290.8501111153	-290.4050193450	-290.9455132908	-290.4051767720	-291.0455189351
7.80	-290.8501109039	-290.4050193410	-290.9455130774	-290.4051767690	-291.0455187192
7.90	-290.8501107114	-290.4050193380	-290.9455128833	-290.4051767670	-291.0455185227
8.00	-290.8501105359	-290.4050193340	-290.9455127063	-290.4051767650	-291.0455183431
8.10	-290.8501103757	-290.4050193320	-290.9455125449	-290.4051767640	-291.0455181798
8.20	-290.8501102294	-290.4050193290	-290.9455123975	-290.4051767620	-291.0455180306
8.30	-290.8501100955	-290.4050193270	-290.9455122625	-290.4051767600	-291.0455178946
8.40	-290.8501099729	-290.4050193250	-290.9455121391	-290.4051767590	-291.0455177697
8.50	-290.8501098604	-290.4050193240	-290.9455120258	-290.4051767580	-291.0455176557
8.60	-290.8501097573	-290.4050193230	-290.9455119219	-290.4051767570	-291.0455175510
8.70	-290.8501096624	-290.4050193220	-290.9455118265	-290.4051767560	-291.0455174549
8.80	-290.8501095751	-290.4050193210	-290.9455117386	-290.4051767550	-291.0455173664
8.90	-290.8501094947	-290.4050193200	-290.9455116578	-290.4051767540	-291.0455172851
9.00	-290.8501094206	-290.4050193200	-290.9455115833	-290.4051767530	-291.0455172108
9.10	-290.8501093522	-290.4050193190	-290.9455115146	-290.4051767520	-291.0455171418
9.20	-290.8501092891	-290.4050193190	-290.9455114511	-290.4051767520	-291.0455170779
9.30	-290.8501092308	-290.4050193190	-290.9455113925	-290.4051767520	-291.0455170190
9.40	-290.8501091768	-290.4050193190	-290.9455113382	-290.4051767510	-291.0455169650
9.50	-290.8501091268	-290.4050193180	-290.9455112880	-290.4051767510	-291.0455169140
9.60	-290.8501090804	-290.4050193180	-290.9455112414	-290.4051767510	-291.0455168672
9.70	-290.8501090375	-290.4050193180	-290.9455111983	-290.4051767500	-291.0455168244
9.80	-290.8501089976	-290.4050193180	-290.9455111582	-290.4051767500	-291.0455167841
9.90	-290.8501089605	-290.4050193180	-290.9455111210	-290.4051767500	-291.0455167468
10.00	-290.8501089260	-290.4050193180	-290.9455110864	-290.4051767500	-291.0455167120
10.10	-290.8501088939	-290.4050193180	-290.9455110542	-290.4051767500	-291.0455166797
10.20	-290.8501088640	-290.4050193180	-290.9455110241	-290.4051767500	-291.0455166495
10.30	-290.8501088362	-290.4050193180	-290.9455109962	-290.4051767500	-291.0455166215
10.40	-290.8501088102	-290.4050193180	-290.9455109701	-290.4051767500	-291.0455165953
10.50	-290.8501087860	-290.4050193180	-290.9455109458	-290.4051767500	-291.0455165709
10.60	-290.8501087633	-290.4050193180	-290.9455109231	-290.4051767500	-291.0455165482

10.70	-290.8501087422	-290.4050193180	-290.9455109019	-290.4051767500	-291.0455165268
10.80	-290.8501087223	-290.4050193180	-290.9455108820	-290.4051767500	-291.0455165069
10.90	-290.8501087038	-290.4050193180	-290.9455108634	-290.4051767500	-291.0455164882
11.00	-290.8501086865	-290.4050193180	-290.9455108460	-290.4051767500	-291.0455164708
11.10	-290.8501086703	-290.4050193180	-290.9455108298	-290.4051767500	-291.0455164545
11.20	-290.8501086550	-290.4050193180	-290.9455108144	-290.4051767500	-291.0455164391
11.30	-290.8501086407	-290.4050193180	-290.9455108001	-290.4051767500	-291.0455164248
11.40	-290.8501086273	-290.4050193180	-290.9455107867	-290.4051767500	-291.0455164113
11.50	-290.8501086147	-290.4050193180	-290.9455107741	-290.4051767500	-291.0455163986
11.60	-290.8501086028	-290.4050193180	-290.9455107622	-290.4051767500	-291.0455163867
11.70	-290.8501085917	-290.4050193180	-290.9455107510	-290.4051767500	-291.0455163755
11.80	-290.8501085812	-290.4050193180	-290.9455107405	-290.4051767500	-291.0455163650
11.90	-290.8501085714	-290.4050193180	-290.9455107306	-290.4051767500	-291.0455163550
12.00	-290.8501085621	-290.4050193180	-290.9455107213	-290.4051767500	-291.0455163457

Table S7: Ne_2 electronic energies (in hartree) calculated at CCSD(T)/aug-cc-pV4Z (CCSD(T)-4Z), CCSD(T)/aug-cc-pV5Z (CCSD(T)-5Z), and CCSD(T)/CBS levels.

R(Å)	CCSD(T)-4Z	SCF-4Z	CCSD(T)-5Z	SCF-5Z	CBS
2.00	-257.6820198143	-257.0731902770	-257.7070088970	-257.0791695350	-257.7298678435
2.10	-257.6870548539	-257.0784825640	-257.7119987964	-257.0844720040	-257.7348046624
2.20	-257.6901938001	-257.0818277560	-257.7150987484	-257.0878249030	-257.7378593729
2.30	-257.6921398026	-257.0839446710	-257.7170113403	-257.0899447390	-257.7397352700
2.40	-257.6933366747	-257.0852843910	-257.7181803622	-257.0912846250	-257.7408749787
2.50	-257.6940645625	-257.0861292950	-257.7188858334	-257.0921310050	-257.7415561016
2.60	-257.6945001539	-257.0866580890	-257.7193040528	-257.0926651200	-257.7419531054
2.70	-257.6947547138	-257.0869859900	-257.7195456632	-257.0930018420	-257.7421761738
2.80	-257.6948981519	-257.0871880210	-257.7196797777	-257.0932140210	-257.7422948053
2.90	-257.6949742693	-257.0873125800	-257.7197493572	-257.0933477500	-257.7423523736
3.00	-257.6950104078	-257.0873900970	-257.7197809377	-257.0934320600	-257.7423753556
3.10	-257.6950233940	-257.0874390940	-257.7197907830	-257.0934851640	-257.7423795983
3.11	-257.6950238655	-257.0874428940	-257.7197909923	-257.0934892410	-257.7423793769
3.12	-257.6950242237	-257.0874465300	-257.7197910782	-257.0934931320	-257.7423790338
3.13	-257.6950244680	-257.0874500100	-257.7197910790	-257.0934968450	-257.7423786483
3.14	-257.6950246106	-257.0874533390	-257.7197909824	-257.0935003890	-257.7423781799
3.15	-257.6950246569	-257.0874565260	-257.7197907972	-257.0935037700	-257.7423776428
3.16	-257.6950246133	-257.0874595770	-257.7197905288	-257.0935069970	-257.7423770396
3.17	-257.6950244854	-257.0874624960	-257.7197901821	-257.0935100750	-257.7423763740
3.18	-257.6950242792	-257.0874652910	-257.7197897636	-257.0935130110	-257.7423756537
3.19	-257.6950239998	-257.0874679660	-257.7197892779	-257.0935158120	-257.7423748807
3.20	-257.6950236522	-257.0874705270	-257.7197887294	-257.0935184840	-257.7423740592
3.21	-257.6950232411	-257.0874729780	-257.7197881231	-257.0935210310	-257.7423731940
3.22	-257.6950227712	-257.0874753250	-257.7197874628	-257.0935234610	-257.7423722874
3.23	-257.6950222466	-257.0874775700	-257.7197867526	-257.0935257780	-257.7423713420
3.24	-257.6950216714	-257.0874797200	-257.7197859964	-257.0935279860	-257.7423703633
3.25	-257.6950210494	-257.0874817760	-257.7197851976	-257.0935300920	-257.7423693510
3.26	-257.6950203843	-257.0874837440	-257.7197843596	-257.0935320990	-257.7423683096
3.27	-257.6950196794	-257.0874856260	-257.7197834861	-257.0935340110	-257.7423672425
3.28	-257.6950189380	-257.0874874270	-257.7197825798	-257.0935358340	-257.7423661508
3.29	-257.6950181632	-257.0874891490	-257.7197816437	-257.0935375710	-257.7423650369
3.30	-257.6950173573	-257.0874907950	-257.7197806775	-257.0935392250	-257.7423638981
3.40	-257.6950081540	-257.0875036810	-257.7197700535	-257.0935519860	-257.7423518537
3.50	-257.6949980877	-257.0875115360	-257.7197588001	-257.0935597350	-257.7423394144
3.60	-257.6949882487	-257.0875159430	-257.7197479208	-257.0935643970	-257.7423273004

3.70	-257.6949791655	-257.0875180430	-257.7197379510	-257.0935672050	-257.7423160026
3.80	-257.6949710455	-257.0875186840	-257.7197290767	-128.5467861750	-257.7422965303
3.90	-257.6949639263	-257.0875184850	-257.7197212947	-257.0935700130	-257.7422885661
4.00	-257.6949577574	-257.0875178670	-257.7197146111	-257.0935707130	-257.7422817670
4.10	-257.6949524504	-257.0875170910	-257.7197089112	-257.0935711600	-257.7422759336
4.20	-257.6949479023	-257.0875163010	-257.7197040251	-257.0935714250	-257.7422710048
4.30	-257.6949440178	-257.0875155610	-257.7196998692	-257.0935715540	-257.7422668482
4.40	-257.6949406944	-257.0875148940	-257.7196963295	-257.0935715810	-257.7422633550
4.50	-257.6949378498	-257.0875143030	-257.7196933191	-257.0935715400	-257.7422603976
4.60	-257.6949354206	-257.0875137830	-257.7196907522	-257.0935714580	-257.7422579340
4.70	-257.6949333357	-257.0875133310	-257.7196885788	-257.0935713570	-257.7422557636
4.80	-257.6949315487	-257.0875129430	-257.7196866825	-257.0935712530	-257.7422539490
4.90	-257.6949300099	-257.0875126180	-257.7196850708	-257.0935711530	-257.7422524004
5.00	-257.6949286825	-257.0875123520	-257.7196836837	-257.0935710630	-257.7422510756
5.10	-257.6949275356	-257.0875121400	-257.7196824865	-257.0935709840	-257.7422499423
5.20	-257.6949265398	-257.0875119760	-257.7196814493	-257.0935709140	-257.7422489715
5.30	-257.6949256742	-257.0875118530	-257.7196805492	-257.0935708520	-257.7422481379
5.40	-257.6949249202	-257.0875117640	-257.7196797654	-257.0935707960	-257.7422474198
5.50	-257.6949242612	-257.0875117010	-257.7196790814	-257.0935707470	-257.7422468005
5.60	-257.6949236848	-257.0875116590	-257.7196784832	-257.0935707020	-257.7422462616
5.70	-257.6949231771	-257.0875116300	-257.7196779575	-257.0935706630	-257.7422457933
5.80	-257.6949227305	-257.0875116130	-257.7196774954	-257.0935706280	-257.7422453828
5.90	-257.6949223366	-257.0875116020	-257.7196770879	-257.0935705970	-257.7422450218
6.00	-257.6949219882	-257.0875115960	-257.7196767279	-257.0935705710	-257.7422447022
6.10	-257.6949216802	-257.0875115920	-257.7196764095	-257.0935705500	-257.7422444194
6.20	-257.6949214053	-257.0875115910	-257.7196761262	-257.0935705320	-257.7422441673
6.30	-257.6949211609	-257.0875115900	-257.7196758744	-257.0935705180	-257.7422439417
6.40	-257.6949209432	-257.0875115890	-257.7196756501	-257.0935705070	-257.7422437404
6.50	-257.6949207486	-257.0875115890	-257.7196754498	-257.0935704980	-257.7422435597
6.60	-257.6949205752	-257.0875115890	-257.7196752709	-257.0935704910	-257.7422433972
6.70	-257.6949204184	-257.0875115890	-257.7196751100	-257.0935704860	-257.7422432509
6.80	-257.6949202778	-257.0875115890	-257.7196749658	-257.0935704830	-257.7422431193
6.90	-257.6949201516	-257.0875115890	-257.7196748361	-257.0935704800	-257.7422430004
7.00	-257.6949200378	-257.0875115890	-257.7196747193	-257.0935704780	-257.7422428926
7.10	-257.6949199358	-257.0875115890	-257.7196746142	-257.0935704770	-257.7422427954
7.20	-257.6949198424	-257.0875115890	-257.7196745186	-257.0935704760	-257.7422427072
7.30	-257.6949197581	-257.0875115890	-257.7196744324	-257.0935704760	-257.7422426272
7.40	-257.6949196819	-257.0875115880	-257.7196743543	-257.0935704750	-257.7422425545
7.50	-257.6949196126	-257.0875115880	-257.7196742834	-257.0935704750	-257.7422424884
7.60	-257.6949195505	-257.0875115880	-257.7196742193	-257.0935704750	-257.7422424283
7.70	-257.6949194927	-257.0875115880	-257.7196741604	-257.0935704750	-257.7422423739
7.80	-257.6949194402	-257.0875115880	-257.7196741070	-257.0935704750	-257.7422423240
7.90	-257.6949193925	-257.0875115880	-257.7196740582	-257.0935704750	-257.7422422785
8.00	-257.6949193490	-257.0875115880	-257.7196740137	-257.0935704750	-257.7422422367
8.10	-257.6949193099	-257.0875115880	-257.7196739734	-257.0935704750	-257.7422421985
8.20	-257.6949192729	-257.0875115880	-257.7196739358	-257.0935704750	-257.7422421639
8.30	-257.6949192392	-257.0875115880	-257.7196739016	-257.0935704750	-257.7422421318
8.40	-257.6949192085	-257.0875115880	-257.7196738703	-257.0935704750	-257.7422421025
8.50	-257.6949191803	-257.0875115880	-257.7196738415	-257.0935704750	-257.7422420753
8.60	-257.6949191551	-257.0875115880	-257.7196738153	-257.0935704750	-257.7422420503
8.70	-257.6949191307	-257.0875115880	-257.7196737906	-257.0935704750	-257.7422420276
8.80	-257.6949191084	-257.0875115880	-257.7196737682	-257.0935704750	-257.7422420065
8.90	-257.6949190881	-257.0875115880	-257.7196737475	-257.0935704750	-257.7422419871
9.00	-257.6949190693	-257.0875115880	-257.7196737284	-257.0935704750	-257.7422419691

9.10	-257.6949190520	-257.0875115880	-257.7196737107	-257.0935704750	-257.7422419525
9.20	-257.6949190359	-257.0875115880	-257.7196736944	-257.0935704750	-257.7422419370
9.30	-257.6949190211	-257.0875115880	-257.7196736792	-257.0935704750	-257.7422419227
9.40	-257.6949190074	-257.0875115880	-257.7196736652	-257.0935704750	-257.7422419095
9.50	-257.6949189947	-257.0875115880	-257.7196736523	-257.0935704750	-257.7422418972
9.60	-257.6949189828	-257.0875115880	-257.7196736402	-257.0935704750	-257.7422418865
9.70	-257.6949189719	-257.0875115880	-257.7196736294	-257.0935704750	-257.7422418751
9.80	-257.6949189616	-257.0875115880	-257.7196736186	-257.0935704750	-257.7422418654
9.90	-257.6949189521	-257.0875115880	-257.7196736090	-257.0935704750	-257.7422418553
10.00	-257.6949189441	-257.0875115880	-257.7196735999	-257.0935704750	-257.7422418473
10.10	-257.6949189353	-257.0875115880	-257.7196735915	-257.0935704750	-257.7422418396
10.20	-257.6949189273	-257.0875115880	-257.7196735837	-257.0935704750	-257.7422418322
10.30	-257.6949189201	-257.0875115880	-257.7196735764	-257.0935704750	-257.7422418253
10.40	-257.6949189134	-257.0875115880	-257.7196735696	-257.0935704750	-257.7422418189
10.50	-257.6949189071	-257.0875115880	-257.7196735632	-257.0935704750	-257.7422418129
10.60	-257.6949189013	-257.0875115880	-257.7196735573	-257.0935704750	-257.7422418079
10.70	-257.6949188959	-257.0875115880	-257.7196735521	-257.0935704750	-257.7422418019
10.80	-257.6949188908	-257.0875115880	-257.7196735465	-257.0935704750	-257.7422417971
10.90	-257.6949188860	-257.0875115880	-257.7196735418	-257.0935704750	-257.7422417923
11.00	-257.6949188815	-257.0875115880	-257.7196735371	-257.0935704750	-257.7422417797
11.10	-257.6949188773	-257.0875115880	-257.7196735288	-257.0935704750	-257.7422417762
11.20	-257.6949188733	-257.0875115880	-257.7196735251	-257.0935704750	-257.7422417728
11.30	-257.6949188696	-257.0875115880	-257.7196735215	-257.0935704750	-257.7422417696
11.40	-257.6949188661	-257.0875115880	-257.7196735182	-257.0935704750	-257.7422417730
11.50	-257.6949188628	-257.0875115880	-257.7196735182	-257.0935704750	-257.7422417698
11.60	-257.6949188598	-257.0875115880	-257.7196735150	-257.0935704750	-257.7422417675
11.70	-257.6949188569	-257.0875115880	-257.7196735124	-257.0935704750	-257.7422417640
11.80	-257.6949188541	-257.0875115880	-257.7196735093	-257.0935704750	-257.7422417615
11.90	-257.6949188515	-257.0875115880	-257.7196735068	-257.0935704750	-257.7422417589

Table S8: Ne-Ar electronic energies (in hartree) calculated at CCSD(T)/aug-cc-pV4Z (CCSD(T)-4Z), CCSD(T)/aug-cc-pV5Z (CCSD(T)-5Z), and CCSD(T)/CBS levels.

R(Å)	CCSD(T)-4Z	SCF-4Z	CCSD(T)-5Z	SCF-5Z	CBS
2.00	-655.8678603251	-655.2991724750	-655.8893126982	-655.8893126982	-655.9097933072
2.10	-655.8860183300	-655.3186612080	-655.9073121981	-655.9073121981	-655.9276430673
2.20	-655.8983450195	-655.3320388020	-655.9195170341	-655.9195170341	-655.9397301125
2.30	-655.9066625598	-655.3411916920	-655.9277406831	-655.9277406831	-655.9478616330
2.40	-655.9122399805	-655.3474373050	-655.9332456317	-655.9332456317	-655.9532947250
2.50	-655.9159547590	-655.3516894830	-655.9369043991	-655.9369043991	-655.9568975754
2.60	-655.9184100630	-655.3545786160	-655.9393164205	-655.9393164205	-655.9592661334
2.70	-655.9200182925	-655.3565375040	-655.9408912847	-655.9408912847	-655.9608070679
2.80	-655.9210600272	-655.3578623090	-655.9419073953	-655.9419073953	-655.9617962845
2.90	-655.9217253059	-655.3587554900	-655.9425530861	-655.9425530861	-655.9624201512
3.00	-655.9221422508	-655.3593555990	-655.9429550836	-655.9429550836	-655.9628040426
3.10	-655.9223968202	-655.3597575520	-655.9431982677	-655.9431982677	-655.9630321500
3.20	-655.9225463902	-655.3600262740	-655.9433391595	-655.9433391595	-655.9631607054
3.30	-655.9226289673	-655.3602059170	-655.9434150739	-655.9434150739	-655.9632268231
3.40	-655.9226695916	-655.3603262150	-655.9434505196	-655.9434505196	-655.9632547368
3.41	-655.9226720234	-655.3603358100	-655.9434524955	-655.9434524955	-655.9632560657
3.42	-655.9226742259	-655.3603450310	-655.9434542561	-655.9434542561	-655.9632572016
3.43	-655.9226762014	-655.3603538920	-655.9434558001	-655.9434558001	-655.9632581389
3.44	-655.9226779612	-655.3603624070	-655.9434571384	-655.9434571384	-655.9632588878
3.45	-655.9226795164	-655.3603705910	-655.9434582818	-655.9434582818	-655.9632594592

3.46	-655.9226808776	-655.3603784560	-655.9434592404	-655.9434592404	-655.9632598623
3.47	-655.9226820550	-655.3603860140	-655.9434600242	-655.9434600242	-655.9632601061
3.48	-655.9226830583	-655.3603932790	-655.9434606425	-655.9434606425	-655.9632602002
3.49	-655.9226838967	-655.3604002600	-655.9434611041	-655.9434611041	-655.9632601520
3.50	-655.9226845790	-655.3604069700	-655.9434614178	-655.9434614178	-655.9632599705
3.51	-655.9226851133	-655.3604134190	-655.9434615916	-655.9434615916	-655.9632596631
3.52	-655.9226855079	-655.3604196170	-655.9434616333	-655.9434616333	-655.9632592375
3.53	-655.9226857702	-655.3604255740	-655.9434615505	-655.9434615505	-655.9632587010
3.54	-655.9226859076	-655.3604312980	-655.9434613502	-655.9434613502	-655.9632580593
3.55	-655.9226859355	-655.3604368000	-655.9434610393	-655.9434610393	-655.9632573104
3.56	-655.9226858437	-655.3604420880	-655.9434606242	-655.9434606242	-655.9632564792
3.57	-655.9226856472	-655.3604471700	-655.9434601112	-655.9434601112	-655.9632555611
3.58	-655.9226853520	-655.3604520540	-655.9434595062	-655.9434595062	-655.9632545619
3.59	-655.9226849640	-655.3604567470	-655.9434588148	-655.9434588148	-655.9632534869
3.60	-655.9226844787	-655.3604612570	-655.9434580454	-655.9434580454	-655.9632523583
3.70	-655.9226759890	-655.3604976880	-655.9434468857	-655.9434468857	-655.9632379366
3.80	-655.9226632845	-655.3605219810	-655.9434319953	-655.9434319953	-655.9632111715
3.90	-655.9226489263	-655.3605379930	-655.9434158345	-655.9434158345	-655.9632021148
4.00	-655.9226344165	-655.3605483680	-655.9433998281	-655.9433998281	-655.9631842303
4.10	-655.9226205859	-655.3605549420	-655.9433847453	-655.9433847453	-655.9631674844
4.20	-655.9226078709	-655.3605589900	-655.9433709859	-655.9433709859	-655.9631522512
4.30	-655.9225964240	-655.3605613900	-655.9433586739	-655.9433586739	-655.9631386433
4.40	-655.9225862717	-655.3605627320	-655.9433478036	-655.9433478036	-655.9631266359
4.50	-655.9225773530	-655.3605634070	-655.9433382774	-655.9433382774	-655.9631161054
4.60	-655.9225695611	-655.3605636700	-655.9433300147	-655.9433300147	-655.9631070074
4.70	-655.9225627938	-655.3605636840	-655.9433228359	-655.9433228359	-655.9630990855
4.80	-655.9225569160	-655.3605635540	-655.9433166295	-655.9433166295	-655.9630922580
4.90	-655.9225518194	-655.3605633450	-655.9433112640	-655.9433112640	-655.9630863687
5.00	-655.9225474022	-655.3605631000	-655.9433066318	-655.9433066318	-655.9630813049
5.10	-655.9225435615	-655.3605628450	-655.9433026127	-655.9433026127	-655.9630769243
5.20	-655.9225402339	-655.3605625930	-655.9432991370	-655.9432991370	-655.9630731473
5.30	-655.9225373368	-655.3605623530	-655.9432961164	-655.9432961164	-655.9630698740
5.40	-655.9225348124	-655.3605621290	-655.9432934905	-655.9432934905	-655.9630670371
5.50	-655.9225326081	-655.3605619220	-655.9432912019	-655.9432912019	-655.9630645701
5.60	-655.9225306765	-655.3605617330	-655.9432892014	-655.9432892014	-655.9630624199
5.70	-655.9225289890	-655.3605615610	-655.9432874536	-655.9432874536	-655.9630605404
5.80	-655.9225275030	-655.3605614070	-655.9432859174	-655.9432859174	-655.9630588918
5.90	-655.9225261881	-655.3605612700	-655.9432845670	-655.9432845670	-655.9630574525
6.00	-655.9225250348	-655.3605611490	-655.9432833763	-655.9432833763	-655.9630561782
6.10	-655.9225240149	-655.3605610460	-655.9432823231	-655.9432823231	-655.9630550541
6.20	-655.9225231150	-655.3605609570	-655.9432813934	-655.9432813934	-655.9630540635
6.30	-655.9225223104	-655.3605608830	-655.9432805664	-655.9432805664	-655.9630531904
6.40	-655.9225215949	-655.3605608220	-655.9432798315	-655.9432798315	-655.9630524182
6.50	-655.9225209568	-655.3605607730	-655.9432791766	-655.9432791766	-655.9630517344
6.60	-655.9225203824	-655.3605607340	-655.9432785911	-655.9432785911	-655.9630511306
6.70	-655.9225198770	-655.3605607030	-655.9432780693	-655.9432780693	-655.9630505877
6.80	-655.9225194193	-655.3605606790	-655.9432776004	-655.9432776004	-655.9630501059
6.90	-655.9225190085	-655.3605606620	-655.9432771798	-655.9432771798	-655.9630496762
7.00	-655.9225186388	-655.3605606480	-655.9432768016	-655.9432768016	-655.9630492906
7.10	-655.9225183014	-655.3605606390	-655.9432764602	-655.9432764602	-655.9630489481
7.20	-655.9225180052	-655.3605606320	-655.9432761537	-655.9432761537	-655.9630486341
7.30	-655.9225177332	-655.3605606270	-655.9432758758	-655.9432758758	-655.9630483527
7.40	-655.9225174871	-655.3605606230	-655.9432756245	-655.9432756245	-655.9630480983
7.50	-655.9225172639	-655.3605606210	-655.9432753967	-655.9432753967	-655.9630478684

7.60	-655.9225170570	-655.3605606190	-655.9432751894	-655.9432751894	-655.9630476630
7.70	-655.9225168773	-655.3605606180	-655.9432750023	-655.9432750023	-655.9630474698
7.80	-655.9225167093	-655.3605606180	-655.9432748311	-655.9432748311	-655.9630472969
7.90	-655.9225165564	-655.3605606170	-655.9432746754	-655.9432746754	-655.9630471392
8.00	-655.9225164169	-655.3605606170	-655.9432745332	-655.9432745332	-655.9630469954
8.10	-655.9225162851	-655.3605606170	-655.9432744028	-655.9432744028	-655.9630468671
8.20	-655.9225161728	-655.3605606170	-655.9432742848	-655.9432742848	-655.9630467436
8.30	-655.9225160658	-655.3605606170	-655.9432741758	-655.9432741758	-655.9630466332
8.40	-655.9225159678	-655.3605606170	-655.9432740762	-655.9432740762	-655.9630465323
8.50	-655.9225158778	-655.3605606170	-655.9432739846	-655.9432739846	-655.9630464392
8.60	-655.9225157908	-655.3605606170	-655.9432739000	-655.9432739000	-655.9630463577
8.70	-655.9225157192	-655.3605606170	-655.9432738235	-655.9432738235	-655.9630462759
8.80	-655.9225156490	-655.3605606170	-655.9432737521	-655.9432737521	-655.9630462033
8.90	-655.9225155844	-655.3605606170	-655.9432736865	-655.9432736865	-655.9630461367
9.00	-655.9225155248	-655.3605606170	-655.9432736260	-655.9432736260	-655.9630460752
9.10	-655.9225154698	-655.3605606170	-655.9432735702	-655.9432735702	-655.9630460185
9.20	-655.9225154189	-655.3605606170	-655.9432735185	-655.9432735185	-655.9630459661
9.30	-655.9225153719	-655.3605606170	-655.9432734708	-655.9432734708	-655.9630459176
9.40	-655.9225153283	-655.3605606170	-655.9432734266	-655.9432734266	-655.9630458727
9.50	-655.9225152879	-655.3605606170	-655.9432733856	-655.9432733856	-655.9630458312
9.60	-655.9225152505	-655.3605606170	-655.9432733478	-655.9432733478	-655.9630457930
9.70	-655.9225152158	-655.3605606170	-655.9432733124	-655.9432733124	-655.9630457567
9.80	-655.9225151834	-655.3605606170	-655.9432732796	-655.9432732796	-655.9630457237
9.90	-655.9225151533	-655.3605606170	-655.9432732492	-655.9432732492	-655.9630456928
10.00	-655.9225151254	-655.3605606170	-655.9432732209	-655.9432732209	-655.9630456641
10.10	-655.9225150993	-655.3605606170	-655.9432731945	-655.9432731945	-655.9630456373
10.20	-655.9225150751	-655.3605606170	-655.9432731699	-655.9432731699	-655.9630456124
10.30	-655.9225150525	-655.3605606170	-655.9432731470	-655.9432731470	-655.9630455892
10.40	-655.9225150314	-655.3605606170	-655.9432731256	-655.9432731256	-655.9630455676
10.50	-655.9225150116	-655.3605606170	-655.9432731057	-655.9432731057	-655.9630455473
10.60	-655.9225149932	-655.3605606170	-655.9432730872	-655.9432730872	-655.9630455288
10.70	-655.9225149761	-655.3605606170	-655.9432730696	-655.9432730696	-655.9630455106
10.80	-655.9225149599	-655.3605606170	-655.9432730533	-655.9432730533	-655.9630454943
10.90	-655.9225149448	-655.3605606170	-655.9432730380	-655.9432730380	-655.9630454788
11.00	-655.9225149306	-655.3605606170	-655.9432730237	-655.9432730237	-655.9630454644
11.10	-655.9225149174	-655.3605606170	-655.9432730103	-655.9432730103	-655.9630454507
11.20	-655.9225149049	-655.3605606170	-655.9432729977	-655.9432729977	-655.9630454380
11.30	-655.9225148933	-655.3605606170	-655.9432729859	-655.9432729859	-655.9630454261
11.40	-655.9225148823	-655.3605606170	-655.9432729748	-655.9432729748	-655.9630454149
11.50	-655.9225148720	-655.3605606170	-655.9432729644	-655.9432729644	-655.9630454044
11.60	-655.9225148623	-655.3605606170	-655.9432729548	-655.9432729548	-655.9630453949
11.70	-655.9225148533	-655.3605606170	-655.9432729454	-655.9432729454	-655.9630453850
11.80	-655.9225148446	-655.3605606170	-655.9432729368	-655.9432729368	-655.9630453764
11.90	-655.9225148366	-655.3605606170	-655.9432729286	-655.9432729286	-655.9630453681
12.00	-655.9225148290	-655.3605606170	-655.9432729209	-655.9432729209	-655.9630453604

Table S9: Ne-Kr electronic energies (in Hartree) calculated at CCSD(T)/aug-cc-pV4Z (CCSD(T)-4Z), CCSD(T)/aug-cc-pV5Z (CCSD(T)-5Z), and CCSD(T)/CBS levels.

R(Å)	CCSD(T)-4Z	SCF-4Z	CCSD(T)-5Z	SCF-5Z	CBS
2.00	-2881.151717788	-2880.506408620	-2881.250978357	-2880.509592860	-2881.353331707
2.10	-2881.177406849	-2880.533798470	-2881.276487968	-2880.536989490	-2881.378649236
2.20	-2881.195405944	-2880.553194960	-2881.294335295	-2880.556389640	-2881.396335274
2.30	-2881.207937990	-2880.566868520	-2881.306735974	-2880.570061470	-2881.408599097

2.40	-2881.216612163	-2880.576472790	-2881.315296369	-2880.579659700	-2881.417043511
2.50	-2881.222579702	-2880.583197760	-2881.321167473	-2880.586377010	-2881.422817742
2.60	-2881.226657984	-2880.587893260	-2881.325166562	-2880.591065660	-2881.426737590
2.70	-2881.229424188	-2880.591163060	-2881.327870061	-2880.594330790	-2881.429377925
2.80	-2881.231284207	-2880.593434300	-2881.329681755	-2880.596599950	-2881.431140086
2.90	-2881.232522065	-2880.595007940	-2881.330883179	-2880.598174030	-2881.432303037
3.00	-2881.233335554	-2880.596095360	-2881.331669606	-2880.599264120	-2881.433059569
3.10	-2881.233861664	-2880.596844720	-2881.332175733	-2880.600017890	-2881.433542253
3.20	-2881.234194768	-2880.597359780	-2881.332494135	-2880.600538430	-2881.433842153
3.30	-2881.234399498	-2880.597713090	-2881.332687969	-2880.600897490	-2881.434021324
3.40	-2881.234519884	-2880.597955240	-2881.332800188	-2880.601144910	-2881.434122014
3.50	-2881.234585677	-2880.598121280	-2881.332859755	-2880.601315200	-2881.434172662
3.60	-2881.234616840	-2880.598235300	-2881.332886049	-2880.601432250	-2881.434192145
3.61	-2881.234618608	-2880.598244520	-2881.332887406	-2880.601441710	-2881.434192935
3.62	-2881.234620174	-2880.598253410	-2881.332888530	-2880.601450830	-2881.434193465
3.63	-2881.234621551	-2880.598261970	-2881.332889522	-2880.601459600	-2881.434193935
3.64	-2881.234622748	-2880.598270220	-2881.332890295	-2880.601468060	-2881.434194147
3.65	-2881.234623773	-2880.598278160	-2881.332890928	-2880.601476200	-2881.434194256
3.66	-2881.234624635	-2880.598285810	-2881.332891407	-2880.601484030	-2881.434194232
3.67	-2881.234625343	-2880.598293180	-2881.332891739	-2880.601491580	-2881.434194070
3.68	-2881.234625904	-2880.598300290	-2881.332891933	-2880.601498850	-2881.434193788
3.69	-2881.234626327	-2880.598307130	-2881.332891996	-2880.601505850	-2881.434193383
3.70	-2881.234626618	-2880.598313720	-2881.332891934	-2880.601512590	-2881.434192866
3.71	-2881.234626784	-2880.598320070	-2881.332891754	-2880.601519080	-2881.434192244
3.72	-2881.234626833	-2880.598326190	-2881.332891463	-2880.601525320	-2881.434191530
3.73	-2881.234626771	-2880.598332080	-2881.332891068	-2880.601531340	-2881.434190712
3.74	-2881.234626603	-2880.598337750	-2881.332890574	-2880.601537130	-2881.434189808
3.75	-2881.234626336	-2880.598343220	-2881.332889986	-2880.601542710	-2881.434188823
3.76	-2881.234625975	-2880.598348490	-2881.332889311	-2880.601548080	-2881.434187762
3.77	-2881.234625524	-2880.598353560	-2881.332888553	-2880.601553250	-2881.434186626
3.78	-2881.234624992	-2880.598358450	-2881.332887718	-2880.601558230	-2881.434185422
3.79	-2881.234624382	-2880.598363160	-2881.332886810	-2880.601563020	-2881.434184156
3.80	-2881.234623696	-2880.598478200	-2881.332885855	-2752.054899610	-2953.650910695
3.90	-2881.234613686	-2880.598478220	-2881.332873195	-2880.601605270	-2881.434208384
4.00	-2881.234600144	-2880.598478050	-2881.332857425	-2880.601630970	-2881.434175741
4.10	-2881.234585255	-2880.598477530	-2881.332840653	-2880.601648470	-2881.434146871
4.20	-2881.234570340	-2880.598476480	-2881.332824178	-2880.601660380	-2881.434121478
4.30	-2881.234556133	-2880.598474610	-2881.332808632	-2880.601668480	-2881.434098927
4.40	-2881.234543016	-2880.598471500	-2881.332794420	-2880.601673980	-2881.434078728
4.50	-2881.234531148	-2880.598466550	-2881.332781630	-2880.601677720	-2881.434060089
4.60	-2881.234520554	-2880.598458920	-2881.332770269	-2880.601680260	-2881.434042210
4.70	-2881.234511184	-2880.598447370	-2881.332760281	-2880.601681980	-2881.434024118
4.80	-2881.234502946	-2880.598430150	-2881.332751527	-2880.601683140	-2881.434004498
4.90	-2881.234495730	-2880.598404780	-2881.332743883	-2880.601683910	-2881.433981719
5.00	-2881.234489427	-2752.054720900	-2881.332737236	-2880.601684410	-2809.218985165
5.10	-2881.234483925	-2880.598478070	-2881.332731461	-2880.601684720	-2881.434009369
5.20	-2881.234479129	-2880.598477890	-2881.332726438	-2880.601684910	-2881.434003899
5.30	-2881.234474939	-2880.598477700	-2881.332722062	-2880.601685010	-2881.433999165
5.40	-2881.234471285	-2880.598477520	-2881.332718250	-2880.601685050	-2881.433995065
5.50	-2881.234468090	-2880.598477360	-2881.332714926	-2880.601685060	-2881.433991508
5.60	-2881.234465295	-2880.598477220	-2881.332712019	-2880.601685050	-2881.433988411
5.70	-2881.234462842	-2880.598477090	-2881.332709478	-2880.601685020	-2881.433985722
5.80	-2881.234460689	-2880.598476980	-2881.332707247	-2880.601684980	-2881.433983370
5.90	-2881.234458793	-2880.598476880	-2881.332705286	-2880.601684950	-2881.433981301

6.00	-2881.234457122	-2880.598476790	-2881.332703559	-2880.601684910	-2881.433979488
6.10	-2881.234455642	-2880.598476700	-2881.332702032	-2880.601684880	-2881.433977878
6.20	-2881.234454330	-2880.598476620	-2881.332700686	-2880.601684850	-2881.433976468
6.30	-2881.234453177	-2880.598476540	-2881.332699490	-2880.601684820	-2881.433975199
6.40	-2881.234452145	-2880.598476470	-2881.332698428	-2880.601684790	-2881.433974083
6.50	-2881.234451226	-2880.598476410	-2881.332697483	-2880.601684770	-2881.433973089
6.60	-2881.234450401	-2880.598476350	-2881.332696638	-2880.601684750	-2881.433972199
6.70	-2881.234449671	-2880.598476300	-2881.332695888	-2880.601684730	-2881.433971410
6.80	-2881.234449014	-2880.598476260	-2881.332695213	-2880.601684710	-2881.433970706
6.90	-2881.234448424	-2880.598476220	-2881.332694608	-2880.601684690	-2881.433970075
7.00	-2881.234447894	-2880.598476180	-2881.332694065	-2880.601684670	-2881.433969507
7.10	-2881.234447412	-2880.598476160	-2881.332693574	-2880.601684660	-2881.433969000
7.20	-2881.234446986	-2880.598476130	-2881.332693136	-2880.601684640	-2881.433968545
7.30	-2881.234446597	-2880.598476110	-2881.332692737	-2880.601684630	-2881.433968130
7.40	-2881.234446245	-2880.598476100	-2881.332692378	-2880.601684620	-2881.433967763
7.50	-2881.234445926	-2880.598476090	-2881.332692052	-2880.601684610	-2881.433967429
7.60	-2881.234445632	-2880.598476080	-2881.332691754	-2880.601684600	-2881.433967127
7.70	-2881.234445374	-2880.598476070	-2881.332691489	-2880.601684600	-2881.433966849
7.80	-2881.234445134	-2880.598476070	-2881.332691244	-2880.601684590	-2881.433966603
7.90	-2881.234444916	-2880.598476060	-2881.332691022	-2880.601684580	-2881.433966377
8.00	-2881.234444718	-2880.598476060	-2881.332690819	-2880.601684580	-2881.433966170
8.10	-2881.234444532	-2880.598476060	-2881.332690631	-2880.601684580	-2881.433965981
8.20	-2881.234444370	-2880.598476060	-2881.332690465	-2880.601684570	-2881.433965816
8.30	-2881.234444218	-2880.598476050	-2881.332690309	-2880.601684570	-2881.433965651
8.40	-2881.234444079	-2880.598476050	-2881.332690168	-2880.601684570	-2881.433965506
8.50	-2881.234443951	-2880.598476050	-2881.332690038	-2880.601684570	-2881.433965374
8.60	-2881.234443829	-2880.598476050	-2881.332689916	-2880.601684570	-2881.433965252
8.70	-2881.234443725	-2880.598476050	-2881.332689809	-2880.601684560	-2881.433965147
8.80	-2881.234443626	-2880.598476050	-2881.332689707	-2880.601684560	-2881.433965043
8.90	-2881.234443534	-2880.598476050	-2881.332689614	-2880.601684560	-2881.433964948
9.00	-2881.234443450	-2880.598476050	-2881.332689528	-2880.601684560	-2881.433964861
9.10	-2881.234443372	-2880.598476050	-2881.332689446	-2880.601684560	-2881.433964775
9.20	-2881.234443300	-2880.598476050	-2881.332689376	-2880.601684560	-2881.433964706
9.30	-2881.234443233	-2880.598476050	-2881.332689308	-2880.601684560	-2881.433964636
9.40	-2881.234443172	-2880.598476050	-2881.332689245	-2880.601684560	-2881.433964573
9.50	-2881.234443114	-2880.598476050	-2881.332689187	-2880.601684560	-2881.433964514
9.60	-2881.234443061	-2880.598476050	-2881.332689131	-2880.601684560	-2881.433964455
9.70	-2881.234443012	-2880.598476050	-2881.332689083	-2880.601684560	-2881.433964409
9.80	-2881.234442967	-2880.598476050	-2881.332689037	-2880.601684560	-2881.433964361
9.90	-2881.234442924	-2880.598476050	-2881.332688994	-2880.601684560	-2881.433964318
10.00	-2881.234442885	-2880.598476050	-2881.332688954	-2880.601684560	-2881.433964277
10.10	-2881.234442848	-2880.598476050	-2881.332688914	-2880.601684560	-2881.433964235
10.20	-2881.234442814	-2880.598476050	-2881.332688882	-2880.601684560	-2881.433964204
10.30	-2881.234442782	-2880.598476050	-2881.332688849	-2880.601684560	-2881.433964171
10.40	-2881.234442752	-2880.598476050	-2881.332688819	-2880.601684560	-2881.433964140
10.50	-2881.234442724	-2880.598476050	-2881.332688791	-2880.601684560	-2881.433964112
10.60	-2881.234442698	-2880.598476050	-2881.332688762	-2880.601684560	-2881.433964080
10.70	-2881.234442674	-2880.598476050	-2881.332688740	-2880.601684560	-2881.433964060
10.80	-2881.234442651	-2880.598476050	-2881.332688717	-2880.601684560	-2881.433964037
10.90	-2881.234442630	-2880.598476050	-2881.332688695	-2880.601684560	-2881.433964015
11.00	-2881.234442610	-2880.598476050	-2881.332688675	-2880.601684560	-2881.433963994
11.10	-2881.234442591	-2880.598476050	-2881.332688654	-2880.601684560	-2881.433963971
11.20	-2881.234442573	-2880.598476050	-2881.332688638	-2880.601684560	-2881.433963957
11.30	-2881.234442557	-2880.598476050	-2881.332688622	-2880.601684560	-2881.433963940

11.40	-2881.234442542	-2880.598476050	-2881.332688606	-2880.601684560	-2881.433963924
11.50	-2881.234442527	-2880.598476050	-2881.332688591	-2880.601684560	-2881.433963909
11.60	-2881.234442513	-2880.598476050	-2881.332688575	-2880.601684560	-2881.433963891
11.70	-2881.234442501	-2880.598476050	-2881.332688565	-2880.601684560	-2881.433963883
11.80	-2881.234442489	-2880.598476050	-2881.332688552	-2880.601684560	-2881.433963870
11.90	-2881.234442477	-2880.598476050	-2881.332688541	-2880.601684560	-2881.433963858
12.00	-2881.234442466	-2880.598476050	-2881.332688530	-2880.601684560	-2881.433963847

Table S10: Ne-Xe electronic energies (in Hartree) calculated at CCSD(T)/aug-cc-pV4Z (CCSD(T)-4Z), CCSD(T)/aug-cc-pV5Z (CCSD(T)-5Z), and CCSD(T)/CBS levels.

R(Å)	CCSD(T)-4Z	SCF-4Z	CCSD(T)-5Z	SCF-5Z	CBS
2.00	-457.3969047015	-456.7044146190	-457.4859568291	-456.7075355530	-457.5776352495
2.10	-457.4326313399	-456.7420063060	-457.5214126407	-456.7451028450	-457.6128206200
2.20	-457.4587109626	-456.7697365770	-457.5472733510	-456.7728261730	-457.6384555521
2.30	-457.4776067072	-456.7900578760	-457.5659850732	-456.7931430190	-457.6569767033
2.40	-457.4912053842	-456.8048689380	-457.5794256070	-456.8079464470	-457.6702556051
2.50	-457.5009305031	-456.8156148260	-457.5890140808	-456.8186814630	-457.6797068215
2.60	-457.5078426538	-456.8233806400	-457.5958090874	-456.8264354750	-457.6863855531
2.70	-457.5127243515	-456.8289730550	-457.6005920185	-456.8320176340	-457.6910706218
2.80	-457.5161486139	-456.8329873580	-457.6039347877	-456.8360247850	-457.6943319080
2.90	-457.5185325113	-456.8358603390	-457.6062529827	-456.8388941520	-457.6965831996
3.00	-457.5201779960	-456.8379109000	-457.6078466428	-456.8409442840	-457.6981227273
3.10	-457.5213025597	-456.8393707800	-457.6089311102	-456.8424062370	-457.6991639620
3.20	-457.5220620531	-456.8404076580	-457.6096599728	-456.8434469730	-457.6998585199
3.30	-457.5225674685	-456.8411424560	-457.6101423303	-456.8441867440	-457.7003138917
3.40	-457.5228974478	-456.8416621520	-457.6104551186	-456.8447118860	-457.7006055840
3.50	-457.5231073860	-456.8420291650	-457.6106522829	-456.8450842120	-457.7007863615
3.60	-457.5232360587	-456.8422881480	-457.6107714505	-456.8453478720	-457.7008929289
3.70	-457.5233104498	-456.8424708940	-457.6108386850	-456.8455343470	-457.7009505599
3.80	-457.5233491916	-456.8425999120	-457.6108719215	-456.8456660580	-457.7009765074
3.90	-457.5233650352	-456.8426910490	-457.6108834103	-456.8457589620	-457.7009824346
3.91	-457.5233657178	-456.8426985400	-457.6108837145	-456.8457665870	-457.7009822665
3.92	-457.5233662681	-456.8427057750	-457.6108838880	-456.8457739490	-457.7009819733
3.93	-457.5233666922	-456.8427127630	-457.6108839430	-456.8457810580	-457.7009815731
3.94	-457.5233669954	-456.8427195120	-457.6108838843	-456.8457879210	-457.7009810707
3.95	-457.5233671839	-456.8427260300	-457.6108837137	-456.8457945480	-457.7009804619
3.96	-457.5233672635	-456.8427323250	-457.6108834475	-456.8458009460	-457.7009797751
3.97	-457.5233672397	-456.8427384050	-457.6108830820	-456.8458071230	-457.7009789967
3.98	-457.5233671179	-456.8427442760	-457.6108826258	-456.8458130870	-457.7009781375
3.99	-457.5233669030	-456.8427499460	-457.6108820826	-456.8458188450	-457.7009772004
4.00	-457.5233666000	-456.8427554220	-457.6108814551	-456.8458244040	-457.7009761856
4.01	-457.5233662145	-456.8427607100	-457.6108807547	-456.8458297700	-457.7009751111
4.02	-457.5233657489	-456.8427658170	-457.6108799786	-456.8458349510	-457.7009739676
4.03	-457.5233652086	-456.8427707480	-457.6108791338	-456.8458399530	-457.7009727635
4.04	-457.5233645986	-456.8427755100	-457.6108782243	-456.8458447820	-457.7009715020
4.05	-457.5233639224	-456.8427801080	-457.6108772520	-456.8458494440	-457.7009701833
4.06	-457.5233631836	-456.8427845470	-457.6108762092	-456.8458539440	-457.7009687873
4.07	-457.5233623858	-456.8427888340	-457.6108751421	-456.8458582880	-457.7009674055
4.08	-457.5233615326	-456.8427929720	-457.6108739946	-456.8458624810	-457.7009659183
4.09	-457.5233606273	-456.8427969680	-457.6108728144	-456.8458665290	-457.7009644206
4.10	-457.5233596733	-456.8428008250	-457.6108715899	-456.8458704370	-457.7009628836
4.20	-457.5233480532	-456.8428327410	-457.6108575171	-456.8458704370	-457.7009641677
4.30	-457.5233341949	-456.8428550490	-457.6108415613	-456.8458742090	-457.7009564245

4.40	-457.5233196437	-456.8428705200	-457.6108251906	-456.8458778500	-457.7009447909
4.50	-457.5233053204	-456.8428811360	-457.6108093208	-456.8458813650	-457.7009312878
4.60	-457.5232917634	-456.8428883280	-457.6107944493	-456.8458847570	-457.7009171722
4.70	-457.5232792473	-456.8428931230	-457.6107808262	-456.8458880320	-457.7009032414
4.80	-457.5232678842	-456.8428962590	-457.6107685357	-456.8458911930	-457.7008899638
4.90	-457.5232576841	-456.8428982570	-457.6107575664	-456.8458942440	-457.7008775960
5.00	-457.5232486009	-456.8428994870	-457.6107478466	-456.8458971880	-457.7008662455
5.10	-457.5232405551	-456.8429002050	-457.6107392774	-456.8459000310	-457.7008559331
5.20	-457.5232334548	-456.8429005870	-457.6107317492	-456.8459027740	-457.7008466297
5.30	-457.5232272032	-456.8429007530	-457.6107251453	-456.8459768030	-457.7007981604
5.40	-457.5232217061	-456.8429007830	-457.6107193580	-456.8459771970	-457.7007918642
5.50	-457.5232168745	-456.8429007310	-457.6107142862	-456.8459774500	-457.7007863690
5.60	-457.5232126261	-456.8429006330	-457.6107098348	-456.8459776050	-457.7007815625
5.70	-457.5232088900	-456.8429005110	-457.6107059386	-456.8459776930	-457.7007773804
5.80	-457.5232056012	-456.8429003790	-457.6107025120	-456.8459777360	-457.7007737108
5.90	-457.5232027029	-456.8429002460	-457.6106994968	-456.8459777490	-457.7007704909
6.00	-457.5232001449	-456.8429001180	-457.6106968397	-456.8459777420	-457.7007676618
6.10	-457.5231978837	-456.8428999960	-457.6106944954	-456.8459777220	-457.7007651732
6.20	-457.5231958819	-456.8428998830	-457.6106924220	-456.8459776960	-457.7007629758
6.30	-457.5231941065	-456.8428997800	-457.6106905855	-456.8459776650	-457.7007610348
6.40	-457.5231925290	-456.8428996850	-457.6106889556	-456.8459776330	-457.7007593145
6.50	-457.5231911252	-456.8428995990	-457.6106875067	-456.8459776020	-457.7007577872
6.60	-457.5231898734	-456.8428995210	-457.6106862160	-456.8459775720	-457.7007564287
6.70	-457.5231887553	-456.8428994510	-457.6106850639	-456.8459775440	-457.7007552175
6.80	-457.5231877498	-456.8428993880	-457.6106840341	-456.8459775200	-457.7007541403
6.90	-457.5231868539	-456.8428993320	-457.6106831113	-456.8459774990	-457.7007531695
7.00	-457.5231860535	-456.8428992820	-457.6106822838	-456.8459774800	-457.7007522961
7.10	-457.5231853290	-456.8428992380	-457.6106815407	-456.8459774640	-457.7007515178
7.20	-457.5231846764	-456.8428992000	-457.6106808711	-456.8459774500	-457.7007508169
7.30	-457.5231840875	-456.8428991670	-457.6106802674	-456.8459774380	-457.7007501858
7.40	-457.5231835555	-456.8428991380	-457.6106797220	-456.8459774270	-457.7007496164
7.50	-457.5231830739	-456.8428991140	-457.6106792283	-456.8459774170	-457.7007491019
7.60	-457.5231826374	-456.8428990940	-457.6106787820	-456.8459774080	-457.7007486393
7.70	-457.5231822412	-456.8428990770	-457.6106783766	-456.8459773990	-457.7007482196
7.80	-457.5231818811	-456.8428990630	-457.6106780082	-456.8459773910	-457.7007478394
7.90	-457.5231815533	-456.8428990510	-457.6106776731	-456.8459773840	-457.7007474937
8.00	-457.5231812545	-456.8428990420	-457.6106773672	-456.8459773770	-457.7007471792
8.10	-457.5231809820	-456.8428990340	-457.6106770893	-456.8459773700	-457.7007468951
8.20	-457.5231807328	-456.8428990280	-457.6106768348	-456.8459773640	-457.7007466350
8.30	-457.5231805050	-456.8428990240	-457.6106766021	-456.8459773580	-457.7007463984
8.40	-457.5231802963	-456.8428990200	-457.6106763890	-456.8459773530	-457.7007461813
8.50	-457.5231801050	-456.8428990170	-457.6106761932	-456.8459773480	-457.7007459818
8.60	-457.5231799294	-456.8428990150	-457.6106760146	-456.8459773440	-457.7007458012
8.70	-457.5231797680	-456.8428990130	-457.6106758498	-456.8459773400	-457.7007456340
8.80	-457.5231796196	-456.8428990120	-457.6106756984	-456.8459773360	-457.7007454811
8.90	-457.5231794829	-456.8428990110	-457.6106755590	-456.8459773330	-457.7007453399
9.00	-457.5231793569	-456.8428990100	-457.6106754305	-456.8459773310	-457.7007452094
9.10	-457.5231792407	-456.8428990100	-457.6106753121	-456.8459773290	-457.7007450896
9.20	-457.5231791333	-456.8428990090	-457.6106752025	-456.8459773270	-457.7007449783
9.30	-457.5231790341	-456.8428990090	-457.6106751014	-456.8459773250	-457.7007448764
9.40	-457.5231789423	-456.8428990090	-457.6106750078	-456.8459773240	-457.7007447815
9.50	-457.5231788573	-456.8428990090	-457.6106749206	-456.8459773230	-457.7007446925
9.60	-457.5231787785	-456.8428990090	-457.6106748410	-456.8459773220	-457.7007446126
9.70	-457.5231787054	-456.8428990090	-457.6106747664	-456.8459773210	-457.7007445371

9.80	-457.5231786376	-456.8428990080	-457.6106746973	-456.8459773200	-457.7007444667
9.90	-457.5231785746	-456.8428990080	-457.6106746325	-456.8459773200	-457.7007444000
10.00	-457.5231785159	-456.8428990080	-457.6106745728	-456.8459773200	-457.7007443393
10.10	-457.5231784615	-456.8428990080	-457.6106745180	-456.8459773190	-457.7007442846
10.20	-457.5231784106	-456.8428990080	-457.6106744662	-456.8459773190	-457.7007442318
10.30	-457.5231783633	-456.8428990080	-457.6106744180	-456.8459773190	-457.7007441828
10.40	-457.5231783191	-456.8428990080	-457.6106743731	-456.8459773190	-457.7007441370
10.50	-457.5231782779	-456.8428990080	-457.6106743306	-456.8459773190	-457.7007440932
10.60	-457.5231782394	-456.8428990080	-457.6106742922	-456.8459773190	-457.7007440548
10.70	-457.5231782034	-456.8428990080	-457.6106742555	-456.8459773190	-457.7007440173
10.80	-457.5231781698	-456.8428990080	-457.6106742213	-456.8459773180	-457.7007439831
10.90	-457.5231781383	-456.8428990080	-457.6106741893	-456.8459773180	-457.7007439506
11.00	-457.5231781088	-456.8428990080	-457.6106741593	-456.8459773180	-457.7007439201
11.10	-457.5231780813	-456.8428990080	-457.6106741314	-456.8459773180	-457.7007438918
11.20	-457.5231780553	-456.8428990080	-457.6106741049	-456.8459773180	-457.7007438648
11.30	-457.5231780310	-456.8428990080	-457.6106740803	-456.8459773180	-457.7007438398
11.40	-457.5231780082	-456.8428990080	-457.6106740571	-456.8459773180	-457.7007438162

Table S11: Ne-Rn electronic energies (in Hartree) calculated at CCSD(T)/aug-cc-pV4Z (CCSD(T)-4Z), CCSD(T)/aug-cc-pV5Z (CCSD(T)-5Z), and CCSD(T)/CBS levels.

R(Å)	CCSD(T)-4Z	SCF-4Z	CCSD(T)-5Z	SCF-5Z	CBS
3.00	-416.7909853672	-416.0808831110	-416.8983245210	-416.0839200620	-417.0092365106
3.10	-416.7924090770	-416.0826858200	-416.8996976785	-416.0857239950	-417.0105559420
3.20	-416.7933902394	-416.0839831620	-416.9006392740	-416.0870245370	-417.0114542268
3.30	-416.7940585163	-416.0849150610	-416.9012769092	-416.0879609190	-417.0120571950
3.40	-416.7945069848	-416.0855832950	-416.9017019679	-416.0886343100	-417.0124547953
3.50	-416.7948021988	-416.0860617690	-416.9019794255	-416.0891180580	-417.0127106605
3.60	-416.7949914885	-416.0864040460	-416.9021553525	-416.0894652250	-417.0128698204
3.70	-416.7951083245	-416.0866488220	-416.9022621523	-416.0897141200	-417.0129637764
3.80	-416.7951762379	-416.0868239260	-416.9023224951	-416.0898923650	-417.0130144118
3.90	-416.7952116728	-416.0869492600	-416.9023621206	-416.0900198630	-417.0130572182
3.91	-416.7952139036	-416.0869596410	-416.9023538558	-416.0900304130	-417.0130378466
3.92	-416.7952159324	-416.0869696820	-416.9023553994	-416.0900406130	-417.0130387918
3.93	-416.7952177720	-416.0869793920	-416.9023567596	-416.0900504760	-417.0130395631
3.94	-416.7952194300	-416.0869887830	-416.9023579501	-416.0900600130	-417.0130401810
3.95	-416.7952209142	-416.0869978660	-416.9023589774	-416.0900692340	-417.0130406515
3.96	-416.7952222328	-416.0870066500	-416.9023598494	-416.0900781490	-417.0130409813
3.97	-416.7952233931	-416.0870151450	-416.9023605730	-416.0900867690	-417.0130411766
3.98	-416.7952244023	-416.0870233610	-416.9023611557	-416.0900951030	-417.0130412454
3.99	-416.7952252619	-416.0870313060	-416.9023616034	-416.0901031610	-417.0130411976
4.00	-416.7952259947	-416.0870389890	-416.9023619232	-416.0901109510	-417.0130410240
4.01	-416.7952265861	-416.0870464200	-416.9023621207	-416.0901184830	-417.0130407514
4.02	-416.7952270566	-416.0870536060	-416.9023622016	-416.0901257650	-417.0130403696
4.03	-416.7952274092	-416.0870605550	-416.9023621721	-416.0901328050	-417.0130398882
4.04	-416.7952276491	-416.0870672750	-416.9023620369	-416.0901396110	-417.0130393111
4.05	-416.7952277813	-416.0870737730	-416.9023618019	-416.0901461920	-417.0130386441
4.06	-416.7952278113	-416.0870800560	-416.9023614720	-416.0901525530	-417.0130378929
4.07	-416.7952277440	-416.0870861320	-416.9023610524	-416.0901587030	-417.0130370621
4.08	-416.7952275842	-416.0870920060	-416.9023605473	-416.0901646480	-417.0130361546
4.09	-416.7952273369	-416.0870976860	-416.9023599608	-416.0901703960	-417.0130351742
4.10	-416.7949914885	-416.0871031780	-416.9023592975	-416.0901759520	-417.0132812265
4.20	-416.7948021988	-416.0871489900	-416.9023492857	-416.0902222840	-417.0134590175
4.30	-416.7945069848	-416.0871815510	-416.9023352520	-416.0902552720	-417.0137397526

4.40	-416.7940585163	-416.0872045620	-416.9023193803	-416.0902787300	-417.0141775020
4.50	-416.7933902394	-416.0872207070	-416.9023030731	-416.0902953930	-417.0148449374
4.60	-416.7924090770	-416.0872319430	-416.9022871996	-416.0903072170	-417.0158414957
4.70	-416.7909853672	-416.0872396950	-416.9022722494	-416.0903156040	-417.0173042317
4.80	-416.7951399669	-416.0872449900	-416.9022584879	-416.0903215530	-417.0129167401
4.90	-416.7951284803	-416.0872485660	-416.9022460090	-416.0903257750	-417.0129028572
5.00	-416.7951181255	-416.0872509460	-416.9022348091	-416.0903287690	-417.0128904258
5.10	-416.7951088529	-416.0872524990	-416.9022248470	-416.0903308900	-417.0128794211
5.20	-416.7951006016	-416.0872534810	-416.9022160218	-416.0903323840	-417.0128697063
5.30	-416.7950932873	-416.0872540730	-416.9022082365	-416.0903334270	-417.0128611733
5.40	-416.7950868200	-416.0872543990	-416.9022014002	-416.0903341470	-417.0128537286
5.50	-416.7950811110	-416.0872545500	-416.9021953720	-416.0903346350	-417.0128471761
5.60	-416.7950760724	-416.0872545850	-416.9021900720	-416.0903349580	-417.0128414401
5.70	-416.7950716296	-416.0872545490	-416.9021854116	-416.0903351650	-417.0128364150
5.80	-416.7950677096	-416.0872544720	-416.9021813102	-416.0903352920	-417.0128320086
5.90	-416.7950642485	-416.0872543730	-416.9021776974	-416.0903353640	-417.0128281405
6.00	-416.7950584844	-416.0872541600	-416.9021745109	-416.0903353990	-417.0128275191
6.10	-416.7950560850	-416.0872540580	-416.9021716964	-416.0903354100	-417.0128242054
6.20	-416.7950539574	-416.0872539620	-416.9021692066	-416.0903354040	-417.0128212851
6.30	-416.7950520666	-416.0872538740	-416.9021670003	-416.0903353870	-417.0128187080
6.40	-416.7950503832	-416.0872537930	-416.9021650421	-416.0903353640	-417.0128164288
6.50	-416.7950488821	-416.0872537190	-416.9021633010	-416.0903353370	-417.0128144095
6.60	-416.7950397338	-416.0872536510	-416.9021617500	-416.0903353080	-417.0128208076
6.70	-416.7950475413	-416.0872535890	-416.9021603661	-416.0903352800	-417.0128097612
6.80	-416.7950463415	-416.0872535320	-416.9021591290	-416.0903352530	-417.0128084681
6.90	-416.7950452662	-416.0872534800	-416.9021580212	-416.0903352280	-417.0128073109
7.00	-416.7950443008	-416.0872534330	-416.9021570274	-416.0903352050	-417.0128062740
7.10	-416.7950434327	-416.0872533900	-416.9021561345	-416.0903351840	-417.0128053425
7.20	-416.7950426507	-416.0872533520	-416.9021553308	-416.0903351650	-417.0128045055
7.30	-416.7950419453	-416.0872533170	-416.9021546064	-416.0903351490	-417.0128037505
7.40	-416.7950413079	-416.0872532860	-416.9021539522	-416.0903351340	-417.0128030697
7.50	-416.7950407311	-416.0872532590	-416.9021533606	-416.0903351210	-417.0128024547
7.60	-416.7950402083	-416.0872532360	-416.9021528248	-416.0903351090	-417.0128018991
7.70	-416.7950393027	-416.0872532160	-416.9021523388	-416.0903350990	-417.0128018477
7.80	-416.7950389102	-416.0872531980	-416.9021518974	-416.0903350890	-417.0128013504
7.90	-416.7950385527	-416.0872531830	-416.9021514958	-416.0903350800	-417.0128008994
8.00	-416.7950382264	-416.0872531710	-416.9021511302	-416.0903350720	-417.0128004901
8.10	-416.7950382271	-416.0872531710	-416.9021507966	-416.0903350640	-417.0127998105
8.20	-416.7950379283	-416.0872531600	-416.9021504921	-416.0903350580	-417.0127994971
8.30	-416.7950376558	-416.0872531520	-416.9021502137	-416.0903350510	-417.0127992118
8.40	-416.7950374061	-416.0872531440	-416.9021499588	-416.0903350460	-417.0127989498
8.50	-416.7950371772	-416.0872531390	-416.9021497252	-416.0903350410	-417.0127987113
8.60	-416.7950369672	-416.0872531340	-416.9021495109	-416.0903350360	-417.0127984925
8.70	-416.7950367743	-416.0872531300	-416.9021493141	-416.0903350320	-417.0127982916
8.80	-416.7950365968	-416.0872531270	-416.9021491331	-416.0903350280	-417.0127981075
8.90	-416.7950364334	-416.0872531250	-416.9021489665	-416.0903350250	-417.0127979381
9.00	-416.7950362828	-416.0872531230	-416.9021488130	-416.0903350230	-417.0127977816
9.10	-416.7950361444	-416.0872531210	-416.9021486714	-416.0903350200	-417.0127976372
9.20	-416.7950360157	-416.0872531200	-416.9021485407	-416.0903350180	-417.0127975050
9.30	-416.7950358971	-416.0872531190	-416.9021484200	-416.0903350160	-417.0127973827
9.40	-416.7950357874	-416.0872531190	-416.9021483083	-416.0903350150	-417.0127972694
9.50	-416.7950356859	-416.0872531180	-416.9021482049	-416.0903350140	-417.0127971640
9.60	-416.7950355917	-416.0872531180	-416.9021481091	-416.0903350130	-417.0127970670
9.70	-416.7950355044	-416.0872531170	-416.9021480203	-416.0903350120	-417.0127969766

9.80	-416.7950354234	-416.0872531170	-416.9021479378	-416.0903350110	-417.0127968932
9.90	-416.7950353482	-416.0872531170	-416.9021478613	-416.0903350100	-417.0127968158
10.00	-416.7950352782	-416.0872531170	-416.9021477900	-416.0903350100	-417.0127967433
10.10	-416.7950352131	-416.0872531170	-416.9021477238	-416.0903350090	-417.0127966765
10.20	-416.7950351524	-416.0872531170	-416.9021476622	-416.0903350090	-417.0127966138
10.30	-416.7950350959	-416.0872531170	-416.9021476047	-416.0903350090	-417.0127965553
10.40	-416.7950350432	-416.0872531170	-416.9021475511	-416.0903350090	-417.0127965008
10.50	-416.7950349940	-416.0872531170	-416.9021475011	-416.0903350080	-417.0127964505
10.60	-416.7950349481	-416.0872531170	-416.9021474545	-416.0903350080	-417.0127964031
10.70	-416.7950349051	-416.0872531170	-416.9021474108	-416.0903350080	-417.0127963587
10.80	-416.7950348650	-416.0872531170	-416.9021473701	-416.0903350080	-417.0127963173
10.90	-416.7950348275	-416.0872531170	-416.9021473319	-416.0903350080	-417.0127962785
11.00	-416.7950347923	-416.0872531170	-416.9021472962	-416.0903350080	-417.0127962422
11.10	-416.7950347593	-416.0872531170	-416.9021472631	-416.0903350080	-417.0127962091
11.20	-416.7950347284	-416.0872531170	-416.9021472312	-416.0903350080	-417.0127961761
11.30	-416.7950346995	-416.0872531170	-416.9021472020	-416.0903350080	-417.0127961466
11.40	-416.7950346723	-416.0872531170	-416.9021471743	-416.0903350080	-417.0127961184
11.50	-416.7950346467	-416.0872531170	-416.9021471484	-416.0903350080	-417.0127960921
11.60	-416.7950346227	-416.0872531170	-416.9021471240	-416.0903350080	-417.0127960673
11.70	-416.7950346001	-416.0872531170	-416.9021471011	-416.0903350080	-417.0127960441
11.80	-416.7950345789	-416.0872531170	-416.9021470795	-416.0903350080	-417.0127960222
11.90	-416.7950345589	-416.0872531170	-416.9021470593	-416.0903350080	-417.0127960016
12.00	-416.7950345401	-416.0872531170	-416.9021470402	-416.0903350080	-417.0127959822

Table S12: Ar_2 electronic energies (in Hartree) calculated at CCSD(T)/aug-cc-pV4Z (CCSD(T)-4Z), CCSD(T)/aug-cc-pV5Z (CCSD(T)-5Z), and CCSD(T)/CBS levels.

R(Å)	CCSD(T)-4Z	SCF-4Z	CCSD(T)-5Z	SCF-5Z	CBS
2.00	-1053.980408483	-1053.442113870	-1053.999982665	-1053.444229560	-1054.019332124
2.10	-1054.029239909	-1053.494882940	-1054.048024348	-1053.496596290	-1054.066769255
2.20	-1054.064749473	-1053.533585410	-1054.082974630	-1053.535049110	-1054.101275064
2.30	-1054.090350425	-1053.561785340	-1054.108177433	-1053.563095580	-1054.126144654
2.40	-1054.108663420	-1053.582221010	-1054.126206277	-1053.583438130	-1054.143928001
2.50	-1054.121665598	-1053.596961840	-1054.139005275	-1053.598123170	-1054.156544310
2.60	-1054.130828373	-1053.607552310	-1054.148022387	-1053.608680480	-1054.165427808
2.70	-1054.137235205	-1053.615134000	-1054.154324348	-1053.616242420	-1054.171630740
2.80	-1054.141677027	-1053.620544230	-1054.158690284	-1053.621640700	-1054.175923715
2.90	-1054.144727153	-1053.624393410	-1054.161685150	-1053.625482460	-1054.178865179
3.00	-1054.146798454	-1053.627124260	-1054.163715923	-1053.628208650	-1054.180856830
3.10	-1054.148186423	-1053.629056510	-1054.165073846	-1053.630138060	-1054.182184634
3.20	-1054.149101274	-1053.630420190	-1054.165966006	-1053.631500220	-1054.183053673
3.30	-1054.149691492	-1053.631380270	-1054.166538675	-1053.632459680	-1054.183609185
3.40	-1054.150061280	-1053.632054740	-1054.166894481	-1053.633134000	-1054.183948531
3.50	-1054.150283203	-1053.632527670	-1054.167104956	-1053.633606920	-1054.184147996
3.60	-1054.150407360	-1053.632858760	-1054.167219524	-1053.633937910	-1054.184253610
3.70	-1054.150468086	-1053.633090210	-1054.167272078	-1053.634169120	-1054.184296302
3.71	-1054.150471640	-1053.633109110	-1054.167274886	-1053.634187990	-1054.184298269
3.72	-1054.150474810	-1053.633127340	-1054.167277318	-1053.634206190	-1054.184299237
3.73	-1054.150477633	-1053.633144920	-1054.167279410	-1053.634223740	-1054.184300205
3.74	-1054.150480098	-1053.633161880	-1054.167281169	-1053.634240670	-1054.184302222
3.75	-1054.150482244	-1053.633178240	-1054.167282629	-1053.634257000	-1054.184304238
3.76	-1054.150484079	-1053.633194030	-1054.167283773	-1053.634272750	-1054.184304212
3.77	-1054.150485618	-1053.633209250	-1054.167284631	-1053.634287940	-1054.184304179
3.78	-1054.150486876	-1053.633223930	-1054.167285218	-1053.634302590	-1054.184303147

3.79	-1054.150487866	-1053.633238090	-1054.167285547	-1053.634316720	-1054.184304164
3.80	-1054.150488601	-1053.633251750	-1054.167285632	-1053.634330350	-1054.184303132
3.81	-1054.150489094	-1053.633264920	-1054.167285485	-1053.634343490	-1054.184301099
3.82	-1054.150489358	-1053.633277620	-1054.167285118	-1053.634356170	-1054.184301111
3.83	-1054.150489404	-1053.633289870	-1054.167284545	-1053.634368390	-1054.184301127
3.84	-1054.150489243	-1053.633301680	-1054.167283775	-1053.634380180	-1054.184299089
3.85	-1054.150488886	-1053.633313080	-1054.167282818	-1053.634391550	-1054.184297057
3.86	-1054.150488344	-1053.633324060	-1054.167281684	-1053.634402510	-1054.184296068
3.87	-1054.150487627	-1053.633334650	-1054.167280384	-1053.634413090	-1054.184291976
3.88	-1054.150486743	-1053.633344870	-1054.167278928	-1053.634423280	-1054.184290992
3.89	-1054.150485702	-1053.633354710	-1054.167277324	-1053.634433110	-1054.184287949
3.90	-1054.150484516	-1053.633364210	-1054.167275576	-1053.634442600	-1054.184286955
4.00	-1054.150466145	-1053.633442250	-1054.167252141	-1053.634520630	-1054.184257714
4.10	-1054.150440212	-1053.633496150	-1054.167221945	-1053.634574820	-1054.184223355
4.20	-1054.150411045	-1053.633533200	-1054.167189197	-1053.634612410	-1054.184185855
4.30	-1054.150381286	-1053.633558510	-1054.167156476	-1053.634638440	-1054.184149302
4.40	-1054.150352526	-1053.633575730	-1054.167125280	-1053.634656420	-1054.184114728
4.50	-1054.150325653	-1053.633587420	-1054.167096411	-1053.634668790	-1054.184083248
4.60	-1054.150301083	-1053.633595340	-1054.167070216	-1053.634677250	-1054.184055895
4.70	-1054.150278975	-1053.633600730	-1054.167046774	-1053.634683000	-1054.184031644
4.80	-1054.150259268	-1053.633604400	-1054.167025986	-1053.634686870	-1054.184009482
4.90	-1054.150241838	-1053.633606920	-1054.167007673	-1053.634689450	-1054.183990399
5.00	-1054.150226499	-1053.633608650	-1054.166991610	-1053.634691170	-1054.183974405
5.10	-1054.150213043	-1053.633609830	-1054.166977559	-1053.634692310	-1054.183959378
5.20	-1054.150201263	-1053.633610620	-1054.166965287	-1053.634693090	-1054.183945335
5.30	-1054.150190960	-1053.633611120	-1054.166954576	-1053.634693630	-1054.183935312
5.40	-1054.150181949	-1053.633611420	-1054.166945241	-1053.634694010	-1054.183924218
5.50	-1054.150174066	-1053.633611570	-1054.166937075	-1053.634694280	-1054.183916151
5.60	-1054.150167161	-1053.633611610	-1054.166929924	-1053.634694470	-1054.183909066
5.70	-1054.150161106	-1053.633611570	-1054.166923675	-1053.634694590	-1054.183902976
5.80	-1054.150155789	-1053.633611480	-1054.166918207	-1053.634694670	-1054.183895832
5.90	-1054.150151110	-1053.633611360	-1054.166913394	-1053.634694710	-1054.183890742
6.00	-1054.150146985	-1053.633611220	-1054.166909157	-1053.634694720	-1054.183886658
6.10	-1054.150143342	-1053.633611080	-1054.166905411	-1053.634694710	-1054.183882585
6.20	-1054.150140119	-1053.633610930	-1054.166902109	-1053.634694680	-1054.183879517
6.30	-1054.150137261	-1053.633610790	-1054.166899195	-1053.634694650	-1054.183876455
6.40	-1054.150134722	-1053.633610660	-1054.166896592	-1053.634694610	-1054.183874405
6.50	-1054.150132461	-1053.633610540	-1054.166894286	-1053.634694560	-1054.183871365
6.60	-1054.150130445	-1053.633610420	-1054.166892231	-1053.634694520	-1054.183869320
6.70	-1054.150128645	-1053.633610320	-1054.166890389	-1053.634694480	-1054.183866238
6.80	-1054.150127032	-1053.633610230	-1054.166888756	-1053.634694440	-1054.183866259
6.90	-1054.150125586	-1053.633610140	-1054.166887285	-1053.634694410	-1054.183863176
7.00	-1054.150124292	-1053.633610070	-1054.166885966	-1053.634694380	-1054.183863202
7.10	-1054.150123119	-1053.633610000	-1054.166884779	-1053.634694350	-1054.183862180
7.20	-1054.150122064	-1053.633609950	-1054.166883710	-1053.634694320	-1054.183861169
7.30	-1054.150121115	-1053.633609900	-1054.166882745	-1053.634694300	-1054.183860152
7.40	-1054.150120255	-1053.633609850	-1054.166881874	-1053.634694270	-1054.183859141
7.50	-1054.150119476	-1053.633609810	-1054.166881085	-1053.634694250	-1054.183858129
7.60	-1054.150118770	-1053.633609780	-1054.166880370	-1053.634694230	-1054.183856075
7.70	-1054.150118129	-1053.633609760	-1054.166879721	-1053.634694210	-1054.183857124
7.80	-1054.150117546	-1053.633609730	-1054.166879131	-1053.634694200	-1054.183855063
7.90	-1054.150117015	-1053.633609710	-1054.166878594	-1053.634694180	-1054.183856113
8.00	-1054.150116531	-1053.633609700	-1054.166878104	-1053.634694170	-1054.183854063
8.10	-1054.150116089	-1053.633609690	-1054.166877658	-1053.634694160	-1054.183855113

8.20	-1054.150115686	-1053.633609680	-1054.166877251	-1053.634694150	-1054.183853063
8.30	-1054.150115316	-1053.633609670	-1054.166876876	-1053.634694140	-1054.183854113
8.40	-1054.150114977	-1053.633609660	-1054.166876534	-1053.634694130	-1054.183854113
8.50	-1054.150114667	-1053.633609660	-1054.166876221	-1053.634694130	-1054.183852063
8.60	-1054.150114381	-1053.633609660	-1054.166875933	-1053.634694120	-1054.183853118
8.70	-1054.150114119	-1053.633609650	-1054.166875669	-1053.634694120	-1054.183853113
8.80	-1054.150113878	-1053.633609650	-1054.166875425	-1053.634694120	-1054.183851063
8.90	-1054.150113656	-1053.633609650	-1054.166875201	-1053.634694110	-1054.183851069
9.00	-1054.150113451	-1053.633609650	-1054.166874995	-1053.634694110	-1054.183852118
9.10	-1054.150113262	-1053.633609650	-1054.166874804	-1053.634694110	-1054.183852118
9.20	-1054.150113087	-1053.633609650	-1054.166874628	-1053.634694110	-1054.183852118
9.30	-1054.150112925	-1053.633609650	-1054.166874466	-1053.634694110	-1054.183850069
9.40	-1054.150112776	-1053.633609650	-1054.166874315	-1053.634694110	-1054.183850069
9.50	-1054.150112638	-1053.633609650	-1054.166874176	-1053.634694110	-1054.183850069
9.60	-1054.150112509	-1053.633609650	-1054.166874047	-1053.634694110	-1054.183850069
9.70	-1054.150112390	-1053.633609650	-1054.166873927	-1053.634694110	-1054.183851118
9.80	-1054.150112279	-1053.633609650	-1054.166873816	-1053.634694110	-1054.183851118
9.90	-1054.150112177	-1053.633609650	-1054.166873712	-1053.634694110	-1054.183851118
10.00	-1054.150112081	-1053.633609650	-1054.166873616	-1053.634694100	-1054.183851124
10.10	-1054.150111992	-1053.633609650	-1054.166873527	-1053.634694100	-1054.183851124
10.20	-1054.150111909	-1053.633609650	-1054.166873443	-1053.634694100	-1054.183849075
10.30	-1054.150111832	-1053.633609650	-1054.166873366	-1053.634694100	-1054.183849075
10.40	-1054.150111760	-1053.633609650	-1054.166873293	-1053.634694100	-1054.183849075
10.50	-1054.150111693	-1053.633609640	-1054.166873226	-1053.634694100	-1054.183849069
10.60	-1054.150111630	-1053.633609640	-1054.166873163	-1053.634694100	-1054.183849069
10.70	-1054.150111571	-1053.633609650	-1054.166873104	-1053.634694100	-1054.183849075
10.80	-1054.150111516	-1053.633609640	-1054.166873049	-1053.634694100	-1054.183849069
10.90	-1054.150111465	-1053.633609640	-1054.166872997	-1053.634694100	-1054.183850118
11.00	-1054.150111416	-1053.633609640	-1054.166872949	-1053.634694100	-1054.183850118
11.10	-1054.150111371	-1053.633609640	-1054.166872903	-1053.634694100	-1054.183850118
11.20	-1054.150111329	-1053.633609640	-1054.166872861	-1053.634694100	-1054.183850118
11.30	-1054.150111289	-1053.633609640	-1054.166872821	-1053.634694100	-1054.183850118
11.40	-1054.150111252	-1053.633609640	-1054.166872784	-1053.634694100	-1054.183850118
11.50	-1054.150111217	-1053.633609640	-1054.166872748	-1053.634694100	-1054.183850118
11.60	-1054.150111184	-1053.633609640	-1054.166872715	-1053.634694100	-1054.183850118
11.70	-1054.150111153	-1053.633609640	-1054.166872684	-1053.634694100	-1054.183850118
11.80	-1054.150111124	-1053.633609640	-1054.166872655	-1053.634694100	-1054.183850118
11.90	-1054.150111097	-1053.633609640	-1054.166872628	-1053.634694100	-1054.183850118
12.00	-1054.150111071	-1053.633609640	-1054.166872602	-1053.634694100	-1054.183850118

Table S13: Ar-Kr electronic energies (in Hartree) calculated at CCSD(T)/aug-cc-pV4Z (CCSD(T)-4Z), CCSD(T)/aug-cc-pV5Z (CCSD(T)-5Z), and CCSD(T)/CBS levels.

R(Å)	CCSD(T)-4Z	SCF-4Z	CCSD(T)-5Z	SCF-5Z	CBS
2.00	-3279.223852976	-3278.605343170	-3279.321535328	-3278.606938400	-3279.423125540
2.10	-3279.288616051	-3278.674721300	-3279.385499255	-3278.676006030	-3279.486425455
2.20	-3279.336728815	-3278.726703530	-3279.433005003	-3278.727785270	-3279.533408372
2.30	-3279.372196054	-3278.765405670	-3279.468007508	-3278.766355340	-3279.567997482
2.40	-3279.398151156	-3278.794060810	-3279.493605212	-3278.794925200	-3279.593268120
2.50	-3279.417013043	-3278.815173910	-3279.512191582	-3278.815983800	-3279.611596043
2.60	-3279.430626307	-3278.830662970	-3279.525592166	-3278.831438400	-3279.624792844
2.70	-3279.440382932	-3278.841982190	-3279.535184635	-3278.842736010	-3279.634225226
2.80	-3279.447323870	-3278.850225090	-3279.541999230	-3278.850965360	-3279.640914877
2.90	-3279.452221847	-3278.856208340	-3279.546800498	-3278.856940090	-3279.645619465

3.00	-3279.455646732	-3278.860538300	-3279.550151907	-3278.861264660	-3279.648896813
3.10	-3279.458016333	-3278.863662930	-3279.552466003	-3278.864385860	-3279.651154601
3.20	-3279.459635300	-3278.865911720	-3279.554043198	-3278.866632500	-3279.652689178
3.30	-3279.460724371	-3278.867526060	-3279.555100797	-3278.868245520	-3279.653714499
3.40	-3279.461442617	-3278.868682160	-3279.555795181	-3278.869400860	-3279.654384275
3.50	-3279.461903902	-3278.869508230	-3279.556238189	-3278.870226490	-3279.654808353
3.60	-3279.462189158	-3278.870097240	-3279.556509157	-3278.870815220	-3279.655064488
3.70	-3279.462355530	-3278.870516410	-3279.556664099	-3278.871234170	-3279.655207562
3.80	-3279.462442930	-3278.870814170	-3279.556742137	-3278.871531730	-3279.655275889
3.90	-3279.462479039	-3278.871025300	-3279.556770419	-3278.871742680	-3279.655296061
3.91	-3279.462480626	-3278.871271260	-3279.556771284	-3278.871760040	-3279.655409792
3.92	-3279.462481902	-3278.871261970	-3279.556771858	-3278.871776810	-3279.655394853
3.93	-3279.462482890	-3278.871252350	-3279.556772156	-3278.871793020	-3279.655379785
3.94	-3279.462483603	-3278.871242390	-3279.556772188	-3278.871808670	-3279.655364580
3.95	-3279.462484054	-3278.871232070	-3279.556771971	-3278.871823780	-3279.655349223
3.96	-3279.462484257	-3278.871221380	-3279.556771517	-3278.871838390	-3279.655333726
3.97	-3279.462484223	-3278.871210320	-3279.556770835	-3278.871852490	-3279.655318066
3.98	-3279.462483965	-3278.871198860	-3279.556769939	-3278.871866120	-3279.655302257
3.99	-3279.462483490	-3278.871187000	-3279.556768840	-3278.871879280	-3279.655286273
4.00	-3279.462482817	-3278.871174720	-3279.556767547	-3278.871891990	-3279.655258899
4.10	-3279.462467064	-3278.871280230	-3279.556746123	-3278.871997500	-3279.655222172
4.20	-3279.462440346	-3278.871354570	-3279.556714553	-3278.872071960	-3279.655181436
4.30	-3279.462408232	-3278.871406800	-3279.556678298	-3278.872124430	-3279.655140100
4.40	-3279.462374287	-3278.871443400	-3279.556640834	-3278.872161350	-3279.655100308
4.50	-3279.462340705	-3278.871468980	-3279.556604317	-3278.872187280	-3279.655070080
4.60	-3279.462308773	-3278.871499210	-3279.556569915	-3278.872205470	-3279.655022237
4.70	-3279.462279194	-3278.871486810	-3279.556538310	-3278.872218190	-3279.654998641
4.80	-3279.462252252	-3278.871507820	-3279.556509676	-3278.872227070	-3279.654971447
4.90	-3279.462228017	-3278.871513780	-3279.556484054	-3278.872233240	-3279.654947432
5.00	-3279.462206404	-3278.871517890	-3279.556461313	-3278.872237510	-3279.654926310
5.10	-3279.462187236	-3278.871520730	-3279.556441222	-3278.872240460	-3279.654907807
5.20	-3279.462170327	-3278.871522680	-3279.556423554	-3278.872242480	-3279.654891611
5.30	-3279.462155434	-3278.871524010	-3279.556408039	-3278.872243860	-3279.654877451
5.40	-3279.462142344	-3278.871524910	-3279.556394438	-3278.872244800	-3279.654865068
5.50	-3279.462130848	-3278.871525510	-3279.556382519	-3278.872245440	-3279.654854235
5.60	-3279.462120747	-3278.871525900	-3279.556372078	-3278.872245890	-3279.654844720
5.70	-3279.462111882	-3278.871526130	-3279.556362915	-3278.872246190	-3279.654836382
5.80	-3279.462104083	-3278.871526260	-3279.556354878	-3278.872246410	-3279.654829067
5.90	-3279.462097218	-3278.871526320	-3279.556347818	-3278.872246560	-3279.654822620
6.00	-3279.462091166	-3278.871526320	-3279.556341601	-3278.872246660	-3279.654816934
6.10	-3279.462085816	-3278.871526280	-3279.556336117	-3278.872246730	-3279.654811911
6.20	-3279.462081096	-3278.871526220	-3279.556331276	-3278.872246770	-3279.654807467
6.30	-3279.462076909	-3278.871526140	-3279.556326991	-3278.872246790	-3279.654803541
6.40	-3279.462073186	-3278.871526060	-3279.556323191	-3278.872246790	-3279.654800051
6.50	-3279.462069881	-3278.871525970	-3279.556319818	-3278.872246780	-3279.654796945
6.60	-3279.462066933	-3278.871525890	-3279.556316812	-3278.872246770	-3279.654794181
6.70	-3279.462064307	-3278.871525810	-3279.556314135	-3278.872246750	-3279.654791718
6.80	-3279.462061956	-3278.871525740	-3279.556311740	-3278.872246720	-3279.654789512
6.90	-3279.462059850	-3278.871525670	-3279.556309596	-3278.872246690	-3279.654787533
7.00	-3279.462057959	-3278.871525600	-3279.556307676	-3278.872246670	-3279.654785770
7.10	-3279.462056254	-3278.871525550	-3279.556305948	-3278.872246640	-3279.654784166
7.20	-3279.462054729	-3278.871525490	-3279.556304396	-3278.872246620	-3279.654782737
7.30	-3279.462053349	-3278.871525450	-3279.556302995	-3278.872246590	-3279.654781433
7.40	-3279.462052104	-3278.871525400	-3279.556301729	-3278.872246570	-3279.654780265

7.50	-3279.462050975	-3278.871525360	-3279.556300587	-3278.872246550	-3279.654779211
7.60	-3279.462049943	-3278.871525320	-3279.556299549	-3278.872246530	-3279.654778242
7.70	-3279.462049024	-3278.871525290	-3279.556298611	-3278.872246520	-3279.654777372
7.80	-3279.462048180	-3278.871525260	-3279.556297757	-3278.872246500	-3279.654776572
7.90	-3279.462047413	-3278.871525230	-3279.556296980	-3278.872246490	-3279.654775860
8.00	-3279.462046713	-3278.871525210	-3279.556296274	-3278.872246470	-3279.654775210
8.10	-3279.462046065	-3278.871525190	-3279.556295628	-3278.872246460	-3279.654774602
8.20	-3279.462045492	-3278.871525170	-3279.556295040	-3278.872246450	-3279.654774052
8.30	-3279.462044958	-3278.871525150	-3279.556294501	-3278.872246440	-3279.654773552
8.40	-3279.462044470	-3278.871525140	-3279.556294007	-3278.872246430	-3279.654773098
8.50	-3279.462044022	-3278.871525130	-3279.556293557	-3278.872246420	-3279.654772682
8.60	-3279.462043602	-3278.871525120	-3279.556293141	-3278.872246420	-3279.654772291
8.70	-3279.462043234	-3278.871525110	-3279.556292762	-3278.872246410	-3279.654771943
8.80	-3279.462042887	-3278.871525110	-3279.556292411	-3278.872246400	-3279.654771610
8.90	-3279.462042567	-3278.871525100	-3279.556292088	-3278.872246400	-3279.654771313
9.00	-3279.462042272	-3278.871525100	-3279.556291792	-3278.872246400	-3279.654771047
9.10	-3279.462041991	-3278.871525090	-3279.556291518	-3278.872246390	-3279.654770780
9.20	-3279.462041749	-3278.871525090	-3279.556291264	-3278.872246390	-3279.654770547
9.30	-3279.462041517	-3278.871525090	-3279.556291032	-3278.872246390	-3279.654770335
9.40	-3279.462041302	-3278.871525090	-3279.556290816	-3278.872246380	-3279.654770126
9.50	-3279.462041104	-3278.871525080	-3279.556290615	-3278.872246380	-3279.654769952
9.60	-3279.462040910	-3278.871525080	-3279.556290431	-3278.872246380	-3279.654769769
9.70	-3279.462040749	-3278.871525080	-3279.556290259	-3278.872246380	-3279.654769606
9.80	-3279.462040590	-3278.871525080	-3279.556290098	-3278.872246380	-3279.654769460
9.90	-3279.462040443	-3278.871525080	-3279.556289951	-3278.872246380	-3279.654769321
10.00	-3279.462040306	-3278.871525080	-3279.556289813	-3278.872246380	-3279.654769199
10.10	-3279.462040169	-3278.871525080	-3279.556289684	-3278.872246380	-3279.654769072
10.20	-3279.462040059	-3278.871525080	-3279.556289566	-3278.872246380	-3279.654768960
10.30	-3279.462039949	-3278.871525080	-3279.556289454	-3278.872246380	-3279.654768854
10.40	-3279.462039845	-3278.871525080	-3279.556289349	-3278.872246380	-3279.654768759
10.50	-3279.462039749	-3278.871525080	-3279.556289254	-3278.872246380	-3279.654768678
10.60	-3279.462039650	-3278.871525080	-3279.556289164	-3278.872246380	-3279.654768581
10.70	-3279.462039575	-3278.871525080	-3279.556289078	-3278.872246380	-3279.654768504
10.80	-3279.462039496	-3278.871525080	-3279.556289000	-3278.872246380	-3279.654768430
10.90	-3279.462039423	-3278.871525080	-3279.556288926	-3278.872246380	-3279.654768358
11.00	-3279.462039354	-3278.871525080	-3279.556288856	-3278.872246380	-3279.654768306
11.10	-3279.462039280	-3278.871525080	-3279.556288793	-3278.872246380	-3279.654768235
11.20	-3279.462039229	-3278.871525080	-3279.556288732	-3278.872246380	-3279.654768175
11.30	-3279.462039172	-3278.871525080	-3279.556288674	-3278.872246380	-3279.654768124
11.40	-3279.462039119	-3278.871525080	-3279.556288621	-3278.872246380	-3279.654768074
11.50	-3279.462039069	-3278.871525080	-3279.556288571	-3278.872246380	-3279.654768034
11.60	-3279.462039012	-3278.871525080	-3279.556288523	-3278.872246380	-3279.654767982
11.70	-3279.462038977	-3278.871525080	-3279.556288480	-3278.872246380	-3279.654767940
11.80	-3279.462038936	-3278.871525080	-3279.556288438	-3278.872246380	-3279.654767898
11.90	-3279.462038897	-3278.871525080	-3279.556288398	-3278.872246380	-3279.654767864
12.00	-3279.462038860	-3278.871525080	-3279.556288362	-3278.872246380	-3279.654767864

Table S14: Ar-Xe electronic energies (in Hartree) calculated at CCSD(T)/aug-cc-pV4Z (CCSD(T)-4Z), CCSD(T)/aug-cc-pV5Z (CCSD(T)-5Z), and CCSD(T)/CBS levels.

R(Å)	CCSD(T)-4Z	SCF-4Z	CCSD(T)-5Z	SCF-5Z	CBS
2.00	-855.4109498880	-854.7419495970	-855.4996815005	-854.7440716950	-855.5915847821
2.10	-855.4969198633	-854.8325837790	-855.5845016184	-854.8341973190	-855.6754841968
2.20	-855.5622915676	-854.9021623640	-855.6489801693	-854.9034341660	-855.7392176548

2.30	-855.6117334969	-854.9553121870	-855.6977293467	-854.9563546110	-855.7873688738
2.40	-855.6489102281	-854.9957125880	-855.7343679739	-854.9966014940	-855.8235291782
2.50	-855.6766963141	-855.0262768950	-855.7617360995	-855.0270639610	-855.8505160010
2.60	-855.6973366542	-855.0492972760	-855.7820520803	-855.0500175400	-855.8705291993
2.70	-855.7125733340	-855.0665641950	-855.7970367271	-855.0672409800	-855.8852738443
2.80	-855.7237488497	-855.0794661240	-855.8080153558	-855.0801145920	-855.8960618113
2.90	-855.7318905085	-855.0890723990	-855.8160019233	-855.0897022230	-855.9038961341
3.00	-855.7377791470	-855.0962013500	-855.8217673039	-855.0968186480	-855.9095392319
3.10	-855.7420043844	-855.1014756080	-855.8258940049	-855.1020842730	-855.9135674005
3.20	-855.7450088234	-855.1053664550	-855.8288196288	-855.1059690170	-855.9164137618
3.30	-855.7471228326	-855.1082289480	-855.8308707821	-855.1088271130	-855.9184014380
3.40	-855.7485917053	-855.1103294610	-855.8322898087	-855.1109244210	-855.9197699678
3.50	-855.7495965870	-855.1118670620	-855.8332554368	-855.1124596780	-855.9206957284
3.60	-855.7502705039	-855.1129899980	-855.8338985339	-855.1157354820	-855.9200970231
3.70	-855.7507105597	-855.1138083000	-855.8343144565	-855.1143979070	-855.9216987831
3.80	-855.7509871891	-855.1144033790	-855.8345721264	-855.1135808900	-855.9227298671
3.90	-855.7511511120	-855.1148352810	-855.8347210889	-855.1154231650	-855.9220707955
4.00	-855.7512385721	-855.1151481580	-855.8347965852	-855.1157354820	-855.9221340541
4.10	-855.7512752303	-855.1153743790	-855.8348236000	-855.1159613280	-855.9221511620
4.11	-855.7512768504	-855.1153932090	-855.8348243524	-855.1159801310	-855.9221510191
4.12	-855.7512781595	-855.1154114340	-855.8348248180	-855.1159983300	-855.9221506142
4.13	-855.7512791735	-855.1154290730	-855.8348250047	-855.1160159450	-855.9221499465
4.14	-855.7512799059	-855.1154461440	-855.8348249265	-855.1160329950	-855.9221490297
4.15	-855.7512803728	-855.1154626660	-855.8348245974	-855.1160494970	-855.9221478766
4.16	-855.7512805804	-855.1154786560	-855.8348240260	-855.1160654700	-855.9221464975
4.17	-855.7512805439	-855.1154941300	-855.8348232237	-855.1160809280	-855.9221449006
4.18	-855.7512802749	-855.1155091060	-855.8348222040	-855.1160958900	-855.9221431013
4.19	-855.7512797842	-855.1155235980	-855.8348209755	-855.1161103700	-855.9221411054
4.20	-855.7512790825	-855.1156450800	-855.8348195526	-855.1161243840	-855.9221993007
4.30	-855.7512625782	-855.1156551680	-855.8347965180	-855.1162419300	-855.9221090453
4.40	-855.7512342443	-855.1157396160	-855.8347627239	-855.1163265460	-855.9220694282
4.50	-855.7511997894	-855.1158001430	-855.8347236706	-855.1163873690	-855.9220253840
4.60	-855.7511629829	-855.1158434270	-855.8346829304	-855.1164310260	-855.9219803072
4.70	-855.7511261736	-855.1158743130	-855.8346428272	-855.1164623110	-855.9219365240
4.80	-855.7510908408	-855.1158963130	-855.8346046911	-855.1164846910	-855.9218952331
4.90	-855.7510578010	-855.1159119610	-855.8345692933	-855.1165006690	-855.9218571759
5.00	-855.7510274577	-855.1159230770	-855.8345369545	-855.1165120510	-855.9218225941
5.10	-855.7509999372	-855.1159309660	-855.8345077697	-855.1165201360	-855.9217915530
5.20	-855.7509752045	-855.1159365560	-855.8344816363	-855.1165258670	-855.9217638707
5.30	-855.7509531206	-855.1159405090	-855.8344583980	-855.1165299190	-855.9217393656
5.40	-855.7509334920	-855.1159432970	-855.8344378023	-855.1165327780	-855.9217177155
5.50	-855.7509160982	-855.1159452510	-855.8344196184	-855.1165347920	-855.9216986689
5.60	-855.7509007178	-855.1159466130	-855.8344035793	-855.1165362130	-855.9216819056
5.70	-855.7508871307	-855.1159475500	-855.8343894528	-855.1165372140	-855.9216671771
5.80	-855.7508751344	-855.1159481840	-855.8343770088	-855.1165379210	-855.9216542225
5.90	-855.7508645409	-855.1159486040	-855.8343660469	-855.1165384210	-855.9216428291
6.00	-855.7508551816	-855.1159488700	-855.8343563790	-855.1165387720	-855.9216327897
6.10	-855.7508469048	-855.1159490290	-855.8343478463	-855.1165390190	-855.9216239391
6.20	-855.7508395784	-855.1159491120	-855.8343403051	-855.1165391900	-855.9216161229
6.30	-855.7508330790	-855.1159491420	-855.8343336313	-855.1165393070	-855.9216092173
6.40	-855.7508273143	-855.1159491350	-855.8343277131	-855.1165393850	-855.9216030904
6.50	-855.7508221888	-855.1159491030	-855.8343224595	-855.1165394340	-855.9215976569
6.60	-855.7508176245	-855.1159490550	-855.8343177855	-855.1165394620	-855.9215928251
6.70	-855.7508135525	-855.1159489960	-855.8343136216	-855.1165394750	-855.9215885244

6.80	-855.7508099144	-855.1159489310	-855.8343099043	-855.1165394770	-855.9215846862
6.90	-855.7508066581	-855.1159488630	-855.8343065816	-855.1165394700	-855.9215812597
7.00	-855.7508037384	-855.1159487950	-855.8343036038	-855.1165394570	-855.9215781900
7.10	-855.7508011159	-855.1159487270	-855.8343009336	-855.1165394400	-855.9215754410
7.20	-855.7507987573	-855.1159486620	-855.8342985318	-855.1165394200	-855.9215729685
7.30	-855.7507966318	-855.1159486000	-855.8342963713	-855.1165393990	-855.9215707485
7.40	-855.7507947135	-855.1159485420	-855.8342944214	-855.1165393780	-855.9215687445
7.50	-855.7507929798	-855.1159484870	-855.8342926619	-855.1165393570	-855.9215669389
7.60	-855.7507914103	-855.1159484370	-855.8342910681	-855.1165393360	-855.9215653035
7.70	-855.7507899874	-855.1159483900	-855.8342896263	-855.1165393180	-855.9215638254
7.80	-855.7507886954	-855.1159483470	-855.8342883162	-855.1165393010	-855.9215624817
7.90	-855.7507875209	-855.1159483090	-855.8342871275	-855.1165392850	-855.9215612657
8.00	-855.7507864517	-855.1159482730	-855.8342860441	-855.1165392720	-855.9215601545
8.10	-855.7507854783	-855.1159482420	-855.8342850587	-855.1165392600	-855.9215591458
8.20	-855.7507845885	-855.1159482130	-855.8342841579	-855.1165392490	-855.9215582234
8.30	-855.7507837747	-855.1159481880	-855.8342833368	-855.1165392390	-855.9215573863
8.40	-855.7507830306	-855.1159481660	-855.8342825840	-855.1165392300	-855.9215566169
8.50	-855.7507823490	-855.1159481460	-855.8342818965	-855.1165392220	-855.9215559165
8.60	-855.7507817239	-855.1159481290	-855.8342812643	-855.1165392150	-855.9215552713
8.70	-855.7507811499	-855.1159481140	-855.8342806858	-855.1165392080	-855.9215546836
8.80	-855.7507806224	-855.1159481010	-855.8342801524	-855.1165392010	-855.9215541407
8.90	-855.7507801370	-855.1159480900	-855.8342796636	-855.1165391950	-855.9215536455
9.00	-855.7507796899	-855.1159480800	-855.8342792117	-855.1165391890	-855.9215531863
9.10	-855.7507792776	-855.1159480720	-855.8342787969	-855.1165391830	-855.9215527676
9.20	-855.7507788973	-855.1159480650	-855.8342784124	-855.1165391780	-855.9215523776
9.30	-855.7507785459	-855.1159480600	-855.8342780589	-855.1165391720	-855.9215520227
9.40	-855.7507782210	-855.1159480550	-855.8342777305	-855.1165391680	-855.9215516899
9.50	-855.7507779203	-855.1159480510	-855.8342774284	-855.1165391630	-855.9215513869
9.60	-855.7507776417	-855.1159480480	-855.8342771466	-855.1165391590	-855.9215511024
9.70	-855.7507773835	-855.1159480460	-855.8342768874	-855.1165391560	-855.9215508425
9.80	-855.7507771440	-855.1159480440	-855.8342766450	-855.1165391530	-855.9215505977
9.90	-855.7507769215	-855.1159480420	-855.8342764217	-855.1165391500	-855.9215503742
10.00	-855.7507767147	-855.1159480410	-855.8342762125	-855.1165391470	-855.9215501635
10.10	-855.7507765224	-855.1159480400	-855.8342760197	-855.1165391450	-855.9215499708
10.20	-855.7507763434	-855.1159480390	-855.8342758384	-855.1165391430	-855.9215497877
10.30	-855.7507761766	-855.1159480390	-855.8342756714	-855.1165391410	-855.9215496215
10.40	-855.7507760212	-855.1159480380	-855.8342755139	-855.1165391400	-855.9215494619
10.50	-855.7507758762	-855.1159480380	-855.8342753688	-855.1165391390	-855.9215493173
10.60	-855.7507757408	-855.1159480380	-855.8342752316	-855.1165391380	-855.9215491787
10.70	-855.7507756144	-855.1159480370	-855.8342751053	-855.1165391370	-855.9215490525
10.80	-855.7507754961	-855.1159480370	-855.8342749853	-855.1165391360	-855.9215489312
10.90	-855.7507753855	-855.1159480370	-855.8342748750	-855.1165391360	-855.9215488213
11.00	-855.7507752820	-855.1159480370	-855.8342747699	-855.1165391350	-855.9215487150
11.10	-855.7507751850	-855.1159480370	-855.8342746732	-855.1165391350	-855.9215486187
11.20	-855.7507750942	-855.1159480370	-855.8342745809	-855.1165391340	-855.9215485254
11.30	-855.7507750089	-855.1159480370	-855.8342744962	-855.1165391340	-855.9215484412
11.40	-855.7507749290	-855.1159480370	-855.8342744148	-855.1165391340	-855.9215483583
11.50	-855.7507748539	-855.1159480370	-855.8342743402	-855.1165391340	-855.9215482844
11.60	-855.7507747833	-855.1159480370	-855.8342742684	-855.1165391340	-855.9215482111
11.70	-855.7507747170	-855.1159480370	-855.8342742027	-855.1165391340	-855.9215481461
11.80	-855.7507746546	-855.1159480370	-855.8342741391	-855.1165391330	-855.9215480817
11.90	-855.7507745959	-855.1159480370	-855.8342740811	-855.1165391330	-855.9215480245
12.00	-855.7507745407	-855.1159480370	-855.8342740246	-855.1165391330	-855.9215479668

Table S15: Ar-Rn electronic energies (in Hartree) calculated at CCSD(T)/aug-cc-pV4Z (CCSD(T)-4Z), CCSD(T)/aug-cc-pV5Z (CCSD(T)-5Z), and CCSD(T)/CBS levels.

R(Å)	CCSD(T)-4Z	SCF-4Z	CCSD(T)-5Z	SCF-5Z	CBS
3.00	-815.0064580283	-814.3364552870	-815.1101898901	-814.3370893770	-815.2186670912
3.10	-815.0115177209	-814.3426687930	-815.1151313930	-814.3432914050	-815.2234910401
3.20	-815.0151576115	-814.3472901780	-815.1186756304	-814.3479045510	-815.2269395487
3.30	-815.0177529721	-814.3507195610	-815.1211931206	-814.3513278480	-815.2293787579
3.40	-815.0195839958	-814.3532588250	-815.1229608124	-814.3538625190	-815.2310825835
3.50	-815.0208591084	-814.3551350400	-815.1241846822	-814.3557352220	-815.2322546632
3.60	-815.0217325994	-814.3565185150	-815.1250170848	-814.3571160000	-815.2330454720
3.70	-815.0223182715	-814.3575366500	-815.1255700527	-814.3581320640	-815.2335652908
3.80	-815.0226995864	-814.3582845030	-815.1259254734	-814.3588783290	-815.2338944358
3.90	-815.0229374108	-814.3588328290	-815.1261428452	-814.3594254400	-815.2340910318
4.00	-815.0230758219	-814.3592341600	-815.1262651074	-814.3598258580	-815.2341968638
4.01	-815.0230855066	-814.3592678040	-815.1262733691	-814.3598594260	-815.2342036752
4.02	-815.0230945485	-814.3593004080	-815.1262810224	-814.3598919560	-815.2342099133
4.03	-815.0231029723	-814.3593320040	-815.1262880902	-814.3599234800	-815.2342155989
4.04	-815.0231108021	-814.3593626220	-815.1262945959	-814.3599540290	-815.2342207539
4.05	-815.0231180609	-814.3593922910	-815.1263005619	-814.3599836320	-815.2342254007
4.06	-815.0231247722	-814.3594210420	-815.1263060093	-814.3600123190	-815.2342295579
4.07	-815.0231309580	-814.3594489010	-815.1263109592	-814.3600401170	-815.2342332455
4.08	-815.0231366390	-814.3594758970	-815.1263154321	-814.3600670540	-815.2342364841
4.09	-815.0231418357	-814.3595020540	-815.1263194479	-814.3600931550	-815.2342392923
4.10	-815.0231465674	-814.3595274000	-815.1263230261	-814.3601184470	-815.2342416907
4.11	-815.0231508543	-814.3595519580	-815.1263261844	-814.3601429540	-815.2342436934
4.12	-815.0231547142	-814.3595757530	-815.1263289406	-814.3601666990	-815.2342453197
4.13	-815.0231581649	-814.3595988080	-815.1263313120	-814.3601897080	-815.2342465846
4.14	-815.0231612235	-814.3596211460	-815.1263333152	-814.3602120010	-815.2342475058
4.15	-815.0231639067	-814.3596427880	-815.1263349667	-814.3602336010	-815.2342480985
4.16	-815.0231662303	-814.3596637550	-815.1263362808	-814.3602545290	-815.2342483752
4.17	-815.0231682102	-814.3596840700	-815.1263372734	-814.3602748070	-815.2342483528
4.18	-815.0231698608	-814.3597037510	-815.1263379578	-814.3602944530	-815.2342480432
4.19	-815.0231711969	-814.3597228170	-815.1263383489	-814.3603134870	-815.2342474608
4.20	-815.0231722325	-814.3597412890	-815.1263384596	-814.3603319280	-815.2342466185
4.21	-815.0231729804	-814.3597591830	-815.1263383025	-814.3603497940	-815.2342455276
4.22	-815.0231734538	-814.3597765170	-815.1263378941	-814.3603671040	-815.2342442076
4.23	-815.0231736652	-814.3597933100	-815.1263372388	-814.3603838730	-815.2342426563
4.24	-815.0231736266	-814.3598095770	-815.1263363510	-814.3604001190	-815.2342408894
4.25	-815.0231733497	-814.3598253350	-815.1263352433	-814.3604158580	-815.2342389207
4.26	-815.0231728454	-814.3598405980	-815.1263339256	-814.3604311050	-815.2342367588
4.27	-815.0231721245	-814.3598553830	-815.1263324081	-814.3604458760	-815.2342344132
4.28	-815.0231711973	-814.3598697050	-815.1263307016	-814.3604601850	-815.2342318965
4.29	-815.0231700742	-814.3598835760	-815.1263288140	-814.3604740470	-815.2342292117
4.30	-815.0231687645	-814.3598970120	-815.1263267562	-814.3604874750	-815.2342263735
4.40	-815.0231473052	-814.3600101650	-815.1262985666	-814.3606006580	-815.2341911058
4.50	-815.0231154618	-814.3600922110	-815.1262611914	-814.3606829070	-815.2341478127
4.60	-815.0230784143	-814.3601515740	-815.1262195290	-814.3607425950	-815.2341011259
4.70	-815.0230395662	-814.3601944370	-815.1261767953	-814.3607858480	-815.2340540964
4.80	-815.0230010922	-814.3602253310	-815.1261350306	-814.3608171460	-815.2340086522
4.90	-815.0229643173	-814.3602475660	-815.1260954545	-814.3608397550	-815.2339659269
5.00	-815.0229299901	-814.3602635550	-815.1260587421	-814.3608560590	-815.2339265352
5.10	-815.0228984688	-814.3602750450	-815.1260251894	-814.3608677910	-815.2338907151
5.20	-815.0228698643	-814.3602832980	-815.1259948618	-814.3608762150	-815.2338584837
5.30	-815.0228441181	-814.3602892200	-815.1259676564	-814.3608822480	-815.2338296849

5.40	-815.0228210891	-814.3602934640	-815.1259433975	-814.3608865610	-815.2338040970
5.50	-815.0228005777	-814.3602964960	-815.1259218517	-814.3608896380	-815.2337814405
5.60	-815.0227823692	-814.3602986520	-815.1259027724	-814.3608918330	-815.2337614257
5.70	-815.0227662238	-814.3603001740	-815.1258859049	-814.3608933990	-815.2337437759
5.80	-815.0227519290	-814.3603012360	-815.1258710033	-814.3608945190	-815.2337282050
5.90	-815.0227392783	-814.3603019670	-815.1258578740	-814.3608953200	-815.2337145342
6.00	-815.0227280815	-814.3603024590	-815.1258462634	-814.3608958940	-815.2337024435
6.10	-815.0227181666	-814.3603027800	-815.1258360052	-814.3608963040	-815.2336917750
6.20	-815.0227093803	-814.3603029780	-815.1258269331	-814.3608965960	-815.2336823502
6.30	-815.0227015860	-814.3603030900	-815.1258189004	-814.3608968020	-815.2336740147
6.40	-815.0226946629	-814.3603031410	-815.1258117778	-814.3608969450	-815.2336666310
6.50	-815.0226885054	-814.3603031500	-815.1258054525	-814.3608970410	-815.2336600808
6.60	-815.0226830206	-814.3603031300	-815.1257998266	-814.3608971030	-815.2336542609
6.70	-815.0226781275	-814.3603030910	-815.1257948142	-814.3608971410	-815.2336490800
6.80	-815.0226737552	-814.3603030410	-815.1257903406	-814.3608971610	-815.2336444607
6.90	-815.0226698419	-814.3603029840	-815.1257863412	-814.3608971680	-815.2336403351
7.00	-815.0226663338	-814.3603029240	-815.1257827593	-814.3608971650	-815.2336366437
7.10	-815.0226631837	-814.3603028640	-815.1257795456	-814.3608971560	-815.2336333348
7.20	-815.0226603505	-814.3603028040	-815.1257766584	-814.3608971420	-815.2336303650
7.30	-815.0226577981	-814.3603027470	-815.1257740595	-814.3608971250	-815.2336276948
7.40	-815.0226554952	-814.3603026920	-815.1257717163	-814.3608971060	-815.2336252891
7.50	-815.0226534143	-814.3603026410	-815.1257696004	-814.3608970860	-815.2336231191
7.60	-815.0226515309	-814.3603025930	-815.1257676868	-814.3608970650	-815.2336211586
7.70	-815.0226498238	-814.3603025480	-815.1257659535	-814.3608970460	-815.2336193831
7.80	-815.0226482744	-814.3603025070	-815.1257643811	-814.3608970260	-815.2336177750
7.90	-815.0226468689	-814.3603024690	-815.1257629528	-814.3608970090	-815.2336163109
8.00	-815.0226455855	-814.3603024330	-815.1257616535	-814.3608969920	-815.2336149841
8.10	-815.0226444169	-814.3603024010	-815.1257604699	-814.3608969760	-815.2336137759
8.20	-815.0226433507	-814.3603023710	-815.1257593903	-814.3608969620	-815.2336126732
8.30	-815.0226423764	-814.3603023440	-815.1257584044	-814.3608969490	-815.2336116673
8.40	-815.0226414851	-814.3603023190	-815.1257575028	-814.3608969380	-815.2336107471
8.50	-815.0226406686	-814.3603022970	-815.1257566774	-814.3608969270	-815.2336099062
8.60	-815.0226399200	-814.3603022770	-815.1257559209	-814.3608969170	-815.2336091356
8.70	-815.0226392327	-814.3603022590	-815.1257552266	-814.3608969080	-815.2336084290
8.80	-815.0226386012	-814.3603022430	-815.1257545889	-814.3608969000	-815.2336077803
8.90	-815.0226380201	-814.3603022280	-815.1257540023	-814.3608968920	-815.2336071840
9.00	-815.0226374850	-814.3603022160	-815.1257534624	-814.3608968850	-815.2336066362
9.10	-815.0226369917	-814.3603022050	-815.1257529646	-814.3608968780	-815.2336061315
9.20	-815.0226365365	-814.3603021950	-815.1257525057	-814.3608968720	-815.2336056664
9.30	-815.0226361161	-814.3603021870	-815.1257520819	-814.3608968670	-815.2336052374
9.40	-815.0226357273	-814.3603021800	-815.1257516901	-814.3608968610	-815.2336048419
9.50	-815.0226353676	-814.3603021740	-815.1257513277	-814.3608968570	-815.2336044756
9.60	-815.0226350345	-814.3603021680	-815.1257509921	-814.3608968520	-815.2336041368
9.70	-815.0226347257	-814.3603021640	-815.1257506811	-814.3608968480	-815.2336038234
9.80	-815.0226344392	-814.3603021600	-815.1257503926	-814.3608968450	-815.2336035323
9.90	-815.0226341732	-814.3603021570	-815.1257501249	-814.3608968420	-815.2336032628
10.00	-815.0226339259	-814.3603021550	-815.1257498760	-814.3608968390	-815.2336030128
10.10	-815.0226336960	-814.3603021530	-815.1257496446	-814.3608968370	-815.2336027799
10.20	-815.0226334821	-814.3603021510	-815.1257494294	-814.3608968340	-815.2336025638
10.30	-815.0226332827	-814.3603021500	-815.1257492289	-814.3608968330	-815.2336023622
10.40	-815.0226330969	-814.3603021490	-815.1257490420	-814.3608968310	-815.2336021747
10.50	-815.0226329236	-814.3603021480	-815.1257488677	-814.3608968300	-815.2336019993
10.60	-815.0226327618	-814.3603021470	-815.1257487051	-814.3608968280	-815.2336018363
10.70	-815.0226326107	-814.3603021470	-815.1257485532	-814.3608968270	-815.2336016842

10.80	-815.0226324695	-814.3603021460	-815.1257484112	-814.3608968270	-815.2336015409
10.90	-815.0226323374	-814.3603021460	-815.1257482784	-814.3608968260	-815.2336014080
11.00	-815.0226322137	-814.3603021460	-815.1257481541	-814.3608968250	-815.2336012836
11.10	-815.0226320979	-814.3603021460	-815.1257480377	-814.3608968250	-815.2336011665
11.20	-815.0226319893	-814.3603021450	-815.1257479287	-814.3608968240	-815.2336010570
11.30	-815.0226318875	-814.3603021450	-815.1257478265	-814.3608968240	-815.2336009544
11.40	-815.0226317920	-814.3603021450	-815.1257477305	-814.3608968240	-815.2336008580
11.50	-815.0226317023	-814.3603021450	-815.1257476405	-814.3608968240	-815.2336007676
11.60	-815.0226316181	-814.3603021450	-815.1257475559	-814.3608968230	-815.2336006832
11.70	-815.0226315389	-814.3603021450	-815.1257474765	-814.3608968230	-815.2336006035
11.80	-815.0226314644	-814.3603021450	-815.1257474017	-814.3608968230	-815.2336005284
11.90	-815.0226313944	-814.3603021450	-815.1257473313	-814.3608968230	-815.2336004577
12.00	-815.0226313284	-814.3603021450	-815.1257472651	-814.3608968230	-815.2336003913

Table S16: Kr_2 electronic energies (in Hartree) calculated at CCSD(T)/aug-cc-pV4Z (CCSD(T)-4Z), CCSD(T)/aug-cc-pV5Z (CCSD(T)-5Z), and CCSD(T)/CBS levels.

R(Å)	CCSD(T)-4Z	SCF-4Z	CCSD(T)-5Z	SCF-5Z	CBS
2.00	-5504.444070238	-5503.744268120	-5504.619534084	-5503.744805270	-5504.803325533
2.10	-5504.529639216	-5503.835167840	-5504.704464797	-5503.835651340	-5504.887616729
2.20	-5504.594130548	-5503.904258180	-5504.768431063	-5503.904709150	-5504.951050384
2.30	-5504.642471455	-5503.956530920	-5504.816325164	-5503.956959170	-5504.998488468
2.40	-5504.678499577	-5503.995899020	-5504.851966252	-5503.996309550	-5505.033733441
2.50	-5504.705193326	-5504.025419240	-5504.878325956	-5504.025815280	-5505.059750812
2.60	-5504.724851257	-5504.047464790	-5504.897700551	-5504.047848990	-5505.078834788
2.70	-5504.739236841	-5504.063865610	-5504.911850638	-5504.064240340	-5505.092743116
2.80	-5504.749694584	-5504.076023400	-5504.922116391	-5504.076390910	-5505.102811493
2.90	-5504.757243042	-5504.085005450	-5504.929511044	-5504.085367830	-5505.110047660
3.00	-5504.762649014	-5504.091620090	-5504.934795871	-5504.091979150	-5505.115207250
3.10	-5504.766486432	-5504.096476570	-5504.938539434	-5504.102983780	-5505.115398352
3.20	-5504.769182616	-5504.100032000	-5504.941163982	-5504.100388230	-5505.121403319
3.30	-5504.771053979	-5504.102627870	-5504.942981290	-5504.096833720	-5505.126619158
3.40	-5504.772333640	-5504.104518290	-5504.944220497	-5504.091979150	-5505.131605212
3.50	-5504.773192341	-5504.105891620	-5504.945048999	-5504.106247550	-5505.125157665
3.60	-5504.773754384	-5504.106886990	-5504.945588420	-5504.107242970	-5505.125673323
3.70	-5504.774109699	-5504.107606810	-5504.945926584	-5504.107962840	-5505.125993464
3.80	-5504.774322819	-2752.054722200	-5504.946126472	-5504.108482320	-3959.040825154
3.90	-5504.774439728	-5504.108500260	-5504.946232930	-5504.108482320	-5505.126485057
4.00	-5504.774492862	-5504.108769050	-5504.946277572	-5504.109125370	-5505.126310532
4.01	-5504.774495612	-5504.108791340	-5504.946279560	-5504.109147680	-5505.126311708
4.02	-5504.774497976	-5504.108812910	-5504.946281157	-5504.109169260	-5505.126312496
4.03	-5504.774499965	-5504.108833770	-5504.946282409	-5504.109190140	-5505.126312963
4.04	-5504.774501603	-5504.108853940	-5504.946283318	-5504.109210330	-5505.126313097
4.05	-5504.774502903	-5504.108873460	-5504.946283905	-5504.109229860	-5505.126312930
4.06	-5504.774503883	-5504.108892330	-5504.946284185	-5504.109248750	-5505.126312465
4.07	-5504.774504556	-5504.108910590	-5504.946284174	-5504.109267030	-5505.126311723
4.08	-5504.774504938	-5504.108928250	-5504.946283881	-5504.109284700	-5505.126310718
4.09	-5504.774505043	-5504.108945330	-5504.946283317	-5504.109301790	-5505.126309446
4.10	-5504.774504883	-5504.108961850	-5504.946282491	-5504.109318330	-5505.126307911
4.11	-5504.774504472	-5504.108977820	-5504.946281432	-5504.109334320	-5505.126306159
4.12	-5504.774503823	-5504.108993270	-5504.946280141	-5504.109349780	-5505.126304190
4.13	-5504.774502947	-5504.109008220	-5504.946278641	-5504.109364740	-5505.126302030
4.14	-5504.774501855	-5504.109022670	-5504.946276947	-5504.109379200	-5505.126299698
4.15	-5504.774500560	-5504.109036640	-5504.946275050	-5504.109393190	-5505.126297159

4.16	-5504.774499070	-5504.109050160	-5504.946272957	-5504.109406720	-5505.126294427
4.17	-5504.774497398	-5504.109063230	-5504.946270698	-5504.109419810	-5505.126291540
4.18	-5504.774495552	-5504.109075870	-5504.946268241	-5504.109432460	-5505.126288437
4.19	-5504.774493541	-5504.109088090	-5504.946265687	-5504.109444690	-5505.126285308
4.20	-5504.774491372	-5504.109099910	-5504.946262980	-5504.109456520	-5505.126282032
4.30	-5504.774462930	-5504.109198640	-5504.946229343	-5504.109555340	-5505.126242893
4.40	-5504.774426632	-5504.109269140	-5504.946188536	-5504.109625880	-5505.126197333
4.50	-5504.774387075	-5504.109319440	-5504.946145065	-5504.109676160	-5505.126149766
4.60	-5504.774347174	-5504.109355270	-5504.946101780	-5504.109711950	-5505.126102953
4.70	-5504.774308689	-5504.109380750	-5504.946060447	-5504.109737410	-5505.126058644
4.80	-5504.774272622	-5504.109398840	-5504.946021959	-5504.109755490	-5505.126017621
4.90	-5504.774239463	-5504.109411630	-5504.945986785	-5504.109768320	-5505.125980310
5.00	-5504.774209404	-5504.109420660	-5504.945955017	-5504.109777410	-5505.125946715
5.10	-5504.774182409	-5504.109426990	-5504.945926609	-5504.109783850	-5505.125916763
5.20	-5504.774158321	-5504.109431420	-5504.945901383	-5504.109788390	-5505.125890281
5.30	-5504.774136947	-5504.109434500	-5504.945879067	-5504.109791590	-5505.125866909
5.40	-5504.774118030	-5504.109436630	-5504.945859352	-5504.109793830	-5505.125846295
5.50	-5504.774101330	-5504.109438090	-5504.945842019	-5504.109795390	-5505.125828244
5.60	-5504.774086611	-5504.109439100	-5504.945826764	-5504.109796470	-5505.125812385
5.70	-5504.774073626	-5504.109439790	-5504.945813357	-5504.109797220	-5505.125798503
5.80	-5504.774062188	-5504.109440260	-5504.945801562	-5504.109797730	-5505.125786310
5.90	-5504.774052104	-5504.109440570	-5504.945791186	-5504.109798070	-5505.125775612
6.00	-5504.774043205	-5504.109440790	-5504.945782037	-5504.109798310	-5505.125766190
6.10	-5504.774035350	-5504.109440930	-5504.945773972	-5504.109798480	-5505.125757887
6.20	-5504.774028390	-5504.109441010	-5504.945766848	-5504.109798600	-5505.125750569
6.30	-5504.774022231	-5504.109441070	-5504.945760546	-5504.109798680	-5505.125744104
6.40	-5504.774016767	-5504.109441090	-5504.945754961	-5504.109798740	-5505.125738370
6.50	-5504.774011911	-5504.109441100	-5504.945750003	-5504.109798780	-5505.125733288
6.60	-5504.774007596	-5504.109441090	-5504.945745593	-5504.109798810	-5505.125728756
6.70	-5504.774003733	-5504.109441070	-5504.945741672	-5504.109798830	-5505.125724753
6.80	-5504.774000277	-5504.109441040	-5504.945738157	-5504.109798840	-5505.125721153
6.90	-5504.773997206	-5504.109441010	-5504.945735021	-5504.109798850	-5505.125717926
7.00	-5504.773994432	-5504.109440970	-5504.945732212	-5504.109798850	-5505.125715057
7.10	-5504.773991967	-5504.109440940	-5504.945729690	-5504.109798850	-5505.125712459
7.20	-5504.773989725	-5504.109440900	-5504.945727421	-5504.109798840	-5505.125710144
7.30	-5504.773987704	-5504.109440860	-5504.945725381	-5504.109798830	-5505.125708068
7.40	-5504.773985888	-5504.109440830	-5504.945723539	-5504.109798820	-5505.125706187
7.50	-5504.773984246	-5504.109440800	-5504.945721874	-5504.109798810	-5505.125704488
7.60	-5504.773982771	-5504.109440770	-5504.945720368	-5504.109798790	-5505.125702944
7.70	-5504.773981423	-5504.109440740	-5504.945719003	-5504.109798780	-5505.125701548
7.80	-5504.773980187	-5504.109440720	-5504.945717764	-5504.109798770	-5505.125700300
7.90	-5504.773979074	-5504.109440700	-5504.945716637	-5504.109798760	-5505.125699154
8.00	-5504.773978059	-5504.109440680	-5504.945715612	-5504.109798750	-5505.125698112
8.10	-5504.773977142	-5504.109440660	-5504.945714677	-5504.109798740	-5505.125697153
8.20	-5504.773976288	-5504.109440640	-5504.945713822	-5504.109798730	-5505.125696291
8.30	-5504.773975516	-5504.109440630	-5504.945713045	-5504.109798720	-5505.125695509
8.40	-5504.773974809	-5504.109440610	-5504.945712331	-5504.109798710	-5505.125694783
8.50	-5504.773974161	-5504.109440600	-5504.945711678	-5504.109798710	-5505.125694118
8.60	-5504.773973576	-5504.109440590	-5504.945711079	-5504.109798700	-5505.125693504
8.70	-5504.773973021	-5504.109440580	-5504.945710529	-5504.109798690	-5505.125692960
8.80	-5504.773972519	-5504.109440570	-5504.945710023	-5504.109798690	-5505.125692445
8.90	-5504.773972057	-5504.109440560	-5504.945709558	-5504.109798680	-5505.125691976
9.00	-5504.773971632	-5504.109440550	-5504.945709130	-5504.109798680	-5505.125691539
9.10	-5504.773971240	-5504.109440550	-5504.945708735	-5504.109798670	-5505.125691147

9.20	-5504.773970878	-5504.109440540	-5504.945708371	-5504.109798670	-5505.125690775
9.30	-5504.773970543	-5504.109440540	-5504.945708034	-5504.109798670	-5505.125690436
9.40	-5504.773970234	-5504.109440530	-5504.945707722	-5504.109798660	-5505.125690122
9.50	-5504.773969947	-5504.109440530	-5504.945707434	-5504.109798660	-5505.125689832
9.60	-5504.773969682	-5504.109440530	-5504.945707167	-5504.109798660	-5505.125689564
9.70	-5504.773969436	-5504.109440520	-5504.945706920	-5504.109798660	-5505.125689309
9.80	-5504.773969207	-5504.109440520	-5504.945706691	-5504.109798660	-5505.125689079
9.90	-5504.773968995	-5504.109440520	-5504.945706477	-5504.109798650	-5505.125688870
10.00	-5504.773968798	-5504.109440510	-5504.945706279	-5504.109798650	-5505.125688665
10.10	-5504.773968615	-5504.109440510	-5504.945706095	-5504.109798650	-5505.125688480
10.20	-5504.773968444	-5504.109440510	-5504.945705924	-5504.109798650	-5505.125688308
10.30	-5504.773968285	-5504.109440510	-5504.945705764	-5504.109798650	-5505.125688147
10.40	-5504.773968136	-5504.109440510	-5504.945705615	-5504.109798650	-5505.125687998
10.50	-5504.773967998	-5504.109440510	-5504.945705476	-5504.109798650	-5505.125687859
10.60	-5504.773967869	-5504.109440510	-5504.945705346	-5504.109798650	-5505.125687728
10.70	-5504.773967748	-5504.109440510	-5504.945705225	-5504.109798650	-5505.125687606
10.80	-5504.773967635	-5504.109440510	-5504.945705112	-5504.109798650	-5505.125687493
10.90	-5504.773967530	-5504.109440510	-5504.945705006	-5504.109798650	-5505.125687386
11.00	-5504.773967441	-5504.109440510	-5504.945704907	-5504.109798650	-5505.125687276
11.10	-5504.773967338	-5504.109440510	-5504.945704814	-5504.109798650	-5505.125687194
11.20	-5504.773967251	-5504.109440510	-5504.945704727	-5504.109798650	-5505.125687106
11.30	-5504.773967170	-5504.109440510	-5504.945704645	-5504.109798650	-5505.125687024
11.40	-5504.773967093	-5504.109440510	-5504.945704568	-5504.109798650	-5505.125686948
11.50	-5504.773967022	-5504.109440510	-5504.945704496	-5504.109798650	-5505.125686875
11.60	-5504.773966954	-5504.109440510	-5504.945704429	-5504.109798650	-5505.125686808
11.70	-5504.773966891	-5504.109440510	-5504.945704365	-5504.109798650	-5505.125686744
11.80	-5504.773966831	-5504.109440510	-5504.945704305	-5504.109798650	-5505.125686684
11.90	-5504.773966775	-5504.109440510	-5504.945704249	-5504.109798650	-5505.125686627
12.00	-5504.773966722	-5504.109440510	-5504.945704196	-5504.109798650	-5505.125686575

Table S17: Kr-Xe electronic energies (in Hartree) calculated at CCSD(T)/aug-cc-pV4Z (CCSD(T)-4Z), CCSD(T)/aug-cc-pV5Z (CCSD(T)-5Z), and CCSD(T)/CBS levels.

R(Å)	CCSD(T)-4Z	SCF-4Z	CCSD(T)-5Z	SCF-5Z	CBS
2.00	-3080.594904091	-3079.843552360	-3080.761620845	-3079.844434630	-3080.936041131
2.10	-3080.709649308	-3079.963628400	-3080.875335033	-3079.964257870	-3081.048815605
2.20	-3080.797335399	-3080.056244250	-3080.962201470	-3080.056729700	-3081.134902986
2.30	-3080.864203177	-3080.127537210	-3081.028399984	-3080.127938830	-3081.200446416
2.40	-3080.915035846	-3080.182275000	-3081.078674784	-3080.182625070	-3081.250164872
2.50	-3080.953526324	-3080.224174410	-3081.116694464	-3080.224490190	-3081.287709863
2.60	-3080.982539179	-3080.256141460	-3081.145308703	-3080.256432810	-3081.315919608
2.70	-3081.004298809	-3080.280448750	-3081.166732359	-3080.280721970	-3081.337000951
2.80	-3081.020529976	-3080.298869970	-3081.182682833	-3080.299129480	-3081.352664631
2.90	-3081.032566166	-3080.312784750	-3081.194486902	-3080.313033920	-3081.364230972
3.00	-3081.041434526	-3080.323262020	-3081.203165194	-3080.323503500	-3081.372714168
3.10	-3081.047922858	-3080.331126600	-3081.209499363	-3080.331362570	-3081.378889687
3.20	-3081.052632573	-3080.337012380	-3081.214085187	-3080.337244590	-3081.383347639
3.30	-3081.056020677	-3080.341404590	-3081.217374646	-3080.341634370	-3081.386534968
3.40	-3081.058432621	-3080.344673220	-3081.219708742	-3080.344901520	-3081.388788218
3.50	-3081.060128296	-3080.347099300	-3081.221343589	-3080.347326750	-3081.390359724
3.60	-3081.061302211	-3080.348895500	-3081.222470332	-3080.349122480	-3081.391437237
3.70	-3081.062099142	-3080.350222160	-3081.223230922	-3080.350448890	-3081.392159841
3.80	-3081.062626228	-3080.351199790	-3081.223730063	-3080.351426390	-3081.392629736
3.90	-3081.062962258	-3080.351918640	-3081.224044601	-3080.352145170	-3081.392921763

4.00	-3081.063164762	-3080.352446100	-3081.224230456	-3080.352672600	-3081.393090167
4.10	-3081.063275421	-3080.352832330	-3081.224328084	-3080.353058850	-3081.393174113
4.20	-3081.063324195	-3080.353114620	-3081.224366461	-3080.353341170	-3081.393201564
4.21	-3081.063326557	-3080.353138260	-3081.224367893	-3080.353364810	-3081.393202021
4.22	-3081.063328527	-3080.353161170	-3081.224368959	-3080.353387730	-3081.393202133
4.23	-3081.063330130	-3080.353183370	-3081.224369677	3080.353409930	-6542.437443383
4.24	-3081.063331382	-3080.353204870	-3081.224370062	-3080.353431440	-3081.393201392
4.25	-3081.063332296	-3080.353225710	-3081.224370131	-3080.353452280	-3081.393200574
4.26	-3081.063332897	-3080.353245900	-3081.224369891	-3080.353472470	-3081.393199452
4.29	-3081.063332909	-3080.353302770	-3081.224367492	-3080.353547140	-3081.393184522
4.30	-3081.063332362	-3080.353320550	-3081.224366171	-3080.353547140	-3081.393192378
4.40	-3081.063314865	-3080.353470530	-3081.224341703	-3080.353697160	-3081.393160575
4.50	-3081.063282082	-3080.353579570	-3081.224303049	-3080.353806240	-3081.393115738
4.60	-3081.063241067	-3080.353658750	-3081.224257038	-3080.353885430	-3081.393064480
4.70	-3081.063196524	-3080.353716160	-3081.224208197	-3080.353942840	-3081.393011131
4.80	-3081.063151514	-3080.353757730	-3081.224159472	-3080.353984400	-3081.392958511
4.90	-3081.063107907	-3080.353787800	-3081.224112670	-3080.354014450	-3081.392908369
5.00	-3081.063066827	-3080.353809500	-3081.224068852	-3080.354036150	-3081.392861678
5.10	-3081.063028856	-3080.353825140	-3081.224028553	-3080.354051800	-3081.392818933
5.20	-3081.062994226	-3080.353836380	-3081.223991926	-3080.354063080	-3081.392780188
5.30	-3081.062962936	-3080.353844430	-3081.223958950	-3080.354071190	-3081.392745408
5.40	-3081.062934863	-3080.353850190	-3081.223929475	-3080.354077020	-3081.392714423
5.50	-3081.062909800	-3080.353854280	-3081.223903233	-3080.354081200	-3081.392686893
5.60	-3081.062887503	-3080.353857180	-3081.223879968	-3080.354084190	-3081.392662563
5.70	-3081.062867714	-3080.353859230	-3081.223859365	-3080.354086330	-3081.392641055
5.80	-3081.062850176	-3080.353860680	-3081.223841162	-3080.354087850	-3081.392622116
5.90	-3081.062834644	-3080.353861690	-3081.223825077	-3080.354088930	-3081.392605411
6.00	-3081.062820894	-3080.353862400	-3081.223810872	-3080.354089690	-3081.392590700
6.10	-3081.062808717	-3080.353862900	-3081.223798311	-3080.354090230	-3081.392577713
6.20	-3081.062797927	-3080.353863240	-3081.223787207	-3080.354090620	-3081.392566252
6.30	-3081.062788357	-3080.353863480	-3081.223777366	-3080.354090890	-3081.392556110
6.40	-3081.062779861	-3080.353863650	-3081.223768650	-3080.354091080	-3081.392547152
6.50	-3081.062772307	-3080.353863760	-3081.223760903	-3080.354091210	-3081.392539191
6.60	-3081.062765583	-3080.353863830	-3081.223754021	-3080.354091310	-3081.392532127
6.70	-3081.062759588	-3080.353863880	-3081.223747885	-3080.354091380	-3081.392525832
6.80	-3081.062754229	-3080.353863900	-3081.223742418	-3080.354091420	-3081.392520239
6.90	-3081.062749446	-3080.353863910	-3081.223737526	-3080.354091460	-3081.392515216
7.00	-3081.062745157	-3080.353863910	-3081.223733153	-3080.354091480	-3081.392510744
7.10	-3081.062741307	-3080.353863900	-3081.223729228	-3080.354091500	-3081.392506723
7.20	-3081.062737847	-3080.353863890	-3081.223725707	-3080.354091510	-3081.392503127
7.30	-3081.062734733	-3080.353863870	-3081.223722536	-3080.354091510	-3081.392499886
7.40	-3081.062731920	-3080.353863850	-3081.223719683	-3080.354091510	-3081.392496981
7.50	-3081.062729383	-3080.353863820	-3081.223717105	-3080.354091510	-3081.392494341
7.60	-3081.062727084	-3080.353863800	-3081.223714779	-3080.354091510	-3081.392491976
7.70	-3081.062725006	-3080.353863770	-3081.223712672	-3080.354091510	-3081.392489820
7.80	-3081.062723121	-3080.353863750	-3081.223710761	-3080.354091500	-3081.392487878
7.90	-3081.062721408	-3080.353863720	-3081.223709025	-3080.354091500	-3081.392486100
8.00	-3081.062719849	-3080.353863700	-3081.223707450	-3080.354091490	-3081.392484502
8.10	-3081.062718429	-3080.353863680	-3081.223706012	-3080.354091490	-3081.392483036
8.20	-3081.062717135	-3080.353863660	-3081.223704705	-3080.354091480	-3081.392481709
8.30	-3081.062715955	-3080.353863640	-3081.223703508	-3080.354091470	-3081.392480489
8.40	-3081.062714872	-3080.353863620	-3081.223702418	-3080.354091470	-3081.392479379
8.50	-3081.062713881	-3080.353863600	-3081.223701416	-3080.354091460	-3081.392478361
8.60	-3081.062712973	-3080.353863590	-3081.223700502	-3080.354091460	-3081.392477434

8.70	-3081.062712140	-3080.353863570	-3081.223699660	-3080.354091450	-3081.392476577
8.80	-3081.062711374	-3080.353863560	-3081.223698889	-3080.354091450	-3081.392475796
8.90	-3081.062710669	-3080.353863550	-3081.223698177	-3080.354091450	-3081.392475071
9.00	-3081.062710021	-3080.353863540	-3081.223697525	-3080.354091440	-3081.392474416
9.10	-3081.062709423	-3080.353863530	-3081.223696923	-3080.354091440	-3081.392473803
9.20	-3081.062708872	-2752.054720290	-3081.223696368	-3080.354091440	-2896.956191432
9.30	-3081.062708363	-3080.353863520	-3081.223695855	-3080.354091440	-3081.392472721
9.40	-3081.062707892	-3080.353863510	-3081.223695381	-3080.354091430	-3081.392472244
9.50	-3081.062707457	-3080.353863500	-3081.223694941	-3080.354091430	-3081.392471794
9.60	-3081.062707053	-3080.353863500	-3081.223694537	-3080.354091430	-3081.392471388
9.70	-3081.062706680	-3080.353863490	-3081.223694160	-3080.354091430	-3081.392471001
9.80	-3081.062706334	-3080.353863490	-3081.223693812	-3080.354091420	-3081.392470658
9.90	-3081.062706012	-3080.353863490	-3081.223693487	-3080.354091420	-3081.392470329
10.00	-3081.062705713	-3080.353863480	-3081.223693187	-3080.354091420	-3081.392470023
10.10	-3081.062705430	-3080.353863480	-3081.223692906	-3080.354091420	-3081.392469744
10.20	-3081.062705176	-3080.353863480	-3081.223692648	-3080.354091420	-3081.392469480
10.30	-3081.062704936	-3080.353863480	-3081.223692404	-3080.354091410	-3081.392469239
10.40	-3081.062704711	-3080.353863480	-3081.223692179	-3080.354091410	-3081.392469014
10.50	-3081.062704502	-3080.353863480	-3081.223691967	-3080.354091410	-3081.392468800
10.60	-3081.062704307	-3080.353863480	-3081.223691772	-3080.354091410	-3081.392468605
10.70	-3081.062704124	-3080.353863480	-3081.223691587	-3080.354091410	-3081.392468418
10.80	-3081.062703954	-3080.353863480	-3081.223691417	-3080.354091410	-3081.392468248
10.90	-3081.062703794	-3080.353863480	-3081.223691256	-3080.354091410	-3081.392468084
11.00	-3081.062703640	-3080.353863480	-3081.223691107	-3080.354091410	-3081.392467941
11.10	-3081.062703505	-3080.353863480	-3081.223690965	-3080.354091410	-3081.392467792
11.20	-3081.062703374	-3080.353863480	-3081.223690835	-3080.354091410	-3081.392467662
11.30	-3081.062703251	-3080.353863480	-3081.223690710	-3080.354091410	-3081.392467535
11.40	-3081.062703136	-3080.353863480	-3081.223690595	-3080.354091410	-3081.392467421
11.50	-3081.062703028	-3080.353863480	-3081.223690485	-3080.354091410	-3081.392467309
11.60	-3081.062702926	-3080.353863480	-3081.223690385	-3080.354091410	-3081.392467210
11.70	-3081.062702831	-3080.353863480	-3081.223690287	-3080.354091410	-3081.392467111
11.80	-3081.062702741	-3080.353863480	-3081.223690199	-3080.354091410	-3081.392467023
11.90	-3081.062702656	-3080.353863480	-3081.223690112	-3080.354091410	-3081.392466935

Table S18: Kr-Rn electronic energies (in Hartree) calculated at CCSD(T)/aug-cc-pV4Z (CCSD(T)-4Z), CCSD(T)/aug-cc-pV5Z (CCSD(T)-5Z), and CCSD(T)/CBS levels.

R(Å)	CCSD(T)-4Z	SCF-4Z	CCSD(T)-5Z	SCF-5Z	CBS
3.00	-3040.308681372	-3039.561825110	-3040.490195391	-3039.562077470	-3040.680494554
3.10	-3040.316338965	-3039.570982370	-3040.497673169	-3039.571227960	-3040.687787477
3.20	-3040.321946690	-3039.577879890	-3040.503135162	-3039.578120620	-3040.693099303
3.30	-3040.326021700	-3039.583062830	-3040.507092527	-3039.583300150	-3040.696935152
3.40	-3040.328956291	-3039.586948230	-3040.509932500	-3039.587183200	-3040.699677174
3.50	-3040.331046955	-3039.589854230	-3040.511947401	-3039.590087620	-3040.701613474
3.60	-3040.332516885	-3039.592022870	-3040.513357006	-3039.592255200	-3040.702960383
3.70	-3040.333533383	-3039.593637740	-3040.514325776	-3039.593869380	-3040.703879464
3.80	-3040.334221330	-3039.594837770	-3040.514976211	-3039.595068940	-3040.704490807
3.90	-3040.334673411	-3039.595727730	-3040.515398989	-3039.595958590	-3040.704883015
4.00	-3040.334958052	-3039.596386460	-3040.515660812	-3039.596617130	-3040.705121003
4.10	-3040.335125421	-3039.596873140	-3040.515810438	-3039.597103680	-3040.705252088
4.20	-3040.335212044	-3039.597232050	-3040.515883161	-3039.597462520	-3040.705310266
4.25	-3040.335233629	-3039.597374290	-3040.515898933	-3039.597604740	-3040.705319951
4.26	-3040.335236557	-3039.597400210	-3040.515900788	-3039.597630650	-3040.705320684
4.27	-3040.335239067	-3039.597425350	-3040.515902240	-3039.597655790	-3040.705321027

4.28	-3040.335241176	-3039.597449730	-3040.515903314	-3039.597680160	-3040.705321022
4.29	-3040.335242902	-3039.597473360	-3040.515904029	-3039.597703800	-3040.705320669
4.30	-3040.335244260	-3039.597496290	-3040.515904397	-3039.597726720	-3040.705320006
4.31	-3040.335245266	-3039.597518510	-3040.515904437	-3039.597748940	-3040.705319031
4.32	-3040.335245937	-3039.597540070	-3040.515904162	-3039.597770490	-3040.705317770
4.33	-3040.335246286	-3039.597560960	-3040.515903586	-3039.597791380	-3040.705316223
4.34	-3040.335246329	-3039.597581230	-3040.515902722	-3039.597811650	-3040.705314407
4.35	-3040.335246078	-3039.597600870	-3040.515901584	-3039.597831290	-3040.705312340
4.36	-3040.335245547	-3039.597619930	-3040.515900179	-3039.597850340	-3040.705310023
4.40	-3040.335240877	-3039.597690510	-3040.515892220	-3039.597920910	-3040.705298618
4.50	-3040.335215172	-3039.597833040	-3040.515859347	-3039.598063430	-3040.705258231
4.60	-3040.335176396	-3039.597937490	-3040.515814612	-3039.598167870	-3040.705207248
4.70	-3040.335130853	-3039.598013930	-3040.515764054	-3039.598244290	-3040.705151440
4.80	-3040.335082759	-3039.598069800	-3040.515711692	-3039.598300150	-3040.705094606
4.90	-3040.335034845	-3039.598110570	-3040.515660100	-3039.598340910	-3040.705039161
5.00	-3040.334988806	-3039.598140280	-3040.515610881	-3039.598370630	-3040.704986599
5.10	-3040.334945616	-3039.598161890	-3040.515564956	-3039.598392270	-3040.704937788
5.20	-3040.334905773	-3039.598177600	-3040.515522755	-3039.598408020	-3040.704893091
5.30	-3040.334869454	-3039.598188980	-3040.515484413	-3039.598419450	-3040.704852598
5.40	-3040.334836638	-3039.598197220	-3040.515449862	-3039.598427750	-3040.704816195
5.50	-3040.334807170	-3039.598203160	-3040.515418924	-3039.598433770	-3040.704783669
5.60	-3040.334780823	-3039.598207440	-3040.515391339	-3039.598438120	-3040.704754744
5.70	-3040.334757346	-3039.598210520	-3040.515366824	-3039.598441260	-3040.704729107
5.80	-3040.334736473	-3039.598212720	-3040.515345073	-3039.598443520	-3040.704706402
5.90	-3040.334717940	-3039.598214300	-3040.515325795	-3039.598445140	-3040.704686319
6.00	-3040.334701498	-3039.598215420	-3040.515308759	-3039.598446310	-3040.704668633
6.10	-3040.334686912	-3039.598216210	-3040.515293647	-3039.598447140	-3040.704652945
6.20	-3040.334673971	-3039.598216770	-3040.515280298	-3039.598447740	-3040.704639146
6.30	-3040.334662483	-3039.598217160	-3040.515268450	-3039.598448170	-3040.704626898
6.40	-3040.334652276	-3039.598217430	-3040.515257941	-3039.598448470	-3040.704616056
6.50	-3040.334643197	-3039.598217620	-3040.515248608	-3039.598448690	-3040.704606438
6.60	-3040.334635112	-3039.598217750	-3040.515240307	-3039.598448850	-3040.704597895
6.70	-3040.334627902	-3039.598217830	-3040.515232917	-3039.598448960	-3040.704590299
6.80	-3040.334621464	-3039.598217890	-3040.515226324	-3039.598449040	-3040.704583533
6.90	-3040.334615706	-3039.598217920	-3040.515220434	-3039.598449100	-3040.704577486
7.00	-3040.334610548	-3039.598217940	-3040.515215160	-3039.598449140	-3040.704572081
7.10	-3040.334605912	-3039.598217940	-3040.515210438	-3039.598449160	-3040.704567255
7.20	-3040.334601754	-3039.598217940	-3040.515206194	-3039.598449180	-3040.704562911
7.30	-3040.334598010	-3039.598217930	-3040.515202380	-3039.598449200	-3040.704559006
7.40	-3040.334594644	-3039.598217920	-3040.515198945	-3039.598449200	-3040.704555493
7.50	-3040.334591596	-3039.598217900	-3040.515195845	-3039.598449210	-3040.704552322
7.60	-3040.334588840	-3039.598217880	-3040.515193045	-3039.598449210	-3040.704549464
7.70	-3040.334586344	-3039.598217870	-3040.515190512	-3039.598449210	-3040.704546887
7.80	-3040.334584080	-3039.598217850	-3040.515188215	-3039.598449200	-3040.704544550
7.90	-3040.334582023	-3039.598217830	-3040.515186130	-3039.598449200	-3040.704542424
8.00	-3040.334580153	-3039.598217810	-3040.515184235	-3039.598449190	-3040.704540497
8.10	-3040.334578449	-3039.598217790	-3040.515184232	-3039.598449190	-3040.704542268
8.20	-3040.334576895	-3039.598217770	-3040.515182509	-3039.598449180	-3040.704540363
8.30	-3040.334575475	-3039.598217750	-3040.515180937	-3039.598449180	-3040.704538619
8.40	-3040.334574178	-3039.598217740	-3040.515179502	-3039.598449170	-3040.704537040
8.50	-3040.334572990	-3039.598217720	-3040.515178191	-3039.598449170	-3040.704535587
8.60	-3040.334571901	-3039.598217710	-3040.515176990	-3039.598449160	-3040.704534270
8.70	-3040.334570902	-3039.598217690	-3040.515175891	-3039.598449160	-3040.704533054
8.80	-3040.334569984	-3039.598217680	-3040.515174883	-3039.598449150	-3040.704531951

9.00	-3040.334568363	-3039.598217670	-3040.515172320	-3039.598449140	-3040.704528400
9.10	-3040.334567647	-3039.598217660	-3040.515171600	-3039.598449140	-3040.704527671
9.20	-3040.334566987	-3039.598217650	-3040.515170935	-3039.598449130	-3040.704527001
10.00	-3040.334563205	-3039.598217600	-3040.515167127	-3039.598449110	-3040.704523149
10.10	-3040.334562873	-3039.598217600	-3040.515166795	-3039.598449110	-3040.704522816
10.20	-3040.334562563	-3039.598217590	-3040.515166484	-3039.598449110	-3040.704522497
11.00	-3040.334560731	-3039.598217580	-3040.515164641	-3039.598449100	-3040.704520645
11.10	-3040.334560564	-3039.598217580	-3040.515164475	-3039.598449100	-3040.704520480
12.00	-3040.334559454	-3039.598217580	-3040.515163361	-3039.598449100	-3040.704519360

Table S19: Xe_2 electronic energies (in Hartree) calculated at CCSD(T)/aug-cc-pV4Z (CCSD(T)-4Z), CCSD(T)/aug-cc-pV5Z (CCSD(T)-5Z), and CCSD(T)/CBS levels.

R(Å)	CCSD(T)-4Z	SCF-4Z	CCSD(T)-5Z	SCF-5Z	CBS
2.00	-656.681306427	-655.876774026	-656.840389778	-655.878248229	-657.006468704
2.10	-656.841347336	-656.042330974	-656.998782822	-656.043292284	-657.163420980
2.20	-656.963472172	-656.169798741	-657.119630350	-656.170450495	-657.283102288
2.30	-657.056976784	-656.268277482	-657.212119737	-656.268742381	-657.374631495
2.40	-657.128659507	-656.344481538	-657.282980897	-656.344832565	-657.444694658
2.50	-657.183590054	-656.403457905	-657.337234334	-656.403737458	-657.498277840
2.60	-657.225610533	-656.449059700	-657.378687195	-656.449292206	-657.539161597
2.70	-657.257667019	-656.484262140	-657.410262512	-656.484461631	-657.570250628
2.80	-657.282035449	-656.511379162	-657.434221238	-656.511553895	-657.593793410
2.90	-657.300481195	-656.532217249	-657.452318452	-656.532372510	-657.611535891
3.00	-657.314375625	-656.548188545	-657.465917764	-656.548328196	-657.624834340
3.10	-657.324783894	-656.560396503	-657.476078003	-656.560523717	-657.634741339
3.20	-657.332532058	-656.569702093	-657.483619548	-656.569819646	-657.642071529
3.30	-657.338259292	-656.576775653	-657.489176364	-656.576885983	-657.647453604
3.40	-657.342458653	-656.582137746	-657.493236582	-656.582242931	-657.651370727
3.50	-657.345509102	-656.586191470	-657.496174674	-656.586293188	-657.654192884
3.60	-657.347700712	-656.589247997	-657.498276499	-656.589347517	-657.656201742
3.70	-657.349254485	-656.591546758	-657.499759303	-656.591644978	-657.657610818
3.80	-657.350337998	-656.593271374	-657.500787258	-656.593368879	-657.658580883
3.90	-657.351077631	-656.594562190	-657.501483758	-656.594659335	-657.659232332
4.00	-657.351568151	-656.595526130	-657.501940967	-656.595623117	-657.659654680
4.10	-657.351880222	-656.596244404	-657.502227418	-656.596341332	-657.659914285
4.20	-657.352066094	-656.596778497	-657.502393545	-656.596875418	-657.660059700
4.30	-657.352164219	-656.597174837	-657.502476345	-656.597271772	-657.660126413
4.31	-657.352170363	-656.597208324	-657.502481151	-656.597305261	-657.660129814
4.32	-657.352175938	-656.597240819	-657.502485424	-656.597337758	-657.660132719
4.33	-657.352180963	-656.597272352	-657.502489176	-656.597369294	-657.660135134
4.34	-657.352185455	-656.597302950	-657.502492425	-656.597399894	-657.660137080
4.35	-657.352189452	-656.597332641	-657.502495223	-656.597429588	-657.660138617
4.36	-657.352192975	-656.597361451	-657.502497550	-656.597458400	-657.660139689
4.37	-657.352196030	-656.597389405	-657.502499434	-656.597486357	-657.660140341
4.38	-657.352198638	-656.597416528	-657.502500920	-656.597513484	-657.660140648
4.39	-657.352200810	-656.597442846	-657.502501998	-656.597539804	-657.660140577
4.40	-657.352202590	-656.597468380	-657.502502684	-656.597565342	-657.660140112
4.41	-657.352203967	-656.597493154	-657.502503020	-656.597590119	-657.660139355
4.42	-657.352204981	-656.597685382	-657.502502988	-656.597614159	-657.660232712
4.43	-657.352205635	-656.597666522	-657.502502639	-656.597637483	-657.660207613
4.44	-657.352205942	-656.597647080	-657.502501957	-656.597660111	-657.660182259
4.45	-657.352205922	-656.597627039	-657.502500971	-656.597682064	-657.660156668
4.46	-657.352205580	-656.597606380	-657.502499683	-656.597703362	-657.660130815

4.47	-657.352204952	-656.597585086	-657.502498136	-656.597724024	-657.660104734
4.48	-657.352204026	-656.597563136	-657.502496309	-656.597744069	-657.660078369
4.49	-657.352202835	-656.597540511	-657.502494219	-656.597763514	-657.660051702
4.50	-657.352201377	-656.597517191	-657.502491899	-656.597782378	-657.660024779
4.60	-657.352175009	-656.597845515	-657.502457749	-656.597942547	-657.660076932
4.70	-657.352133613	-656.597963488	-657.502409881	-656.598060550	-657.660022256
4.80	-657.352084166	-656.598050268	-657.502355001	-656.598147346	-657.659961667
4.90	-657.352031438	-656.598114013	-657.502297604	-656.598211089	-657.659899373
5.00	-657.351978561	-656.598160777	-657.502240645	-656.598257833	-657.659838142
5.10	-657.351927438	-656.598195042	-657.502185990	-656.598292064	-657.659779801
5.20	-657.351879260	-656.598220115	-657.502134725	-656.598317097	-657.659725319
5.30	-657.351834646	-656.598238436	-657.502087449	-656.598335380	-657.659675272
5.40	-657.351793843	-656.598251798	-657.502044295	-656.598348718	-657.659629664
5.50	-657.351756858	-656.598261524	-657.502005287	-656.598358436	-657.659588539
5.60	-657.351723556	-656.598268586	-657.501970251	-656.598365508	-657.659551678
5.70	-657.351693708	-656.598273698	-657.501938931	-656.598370650	-657.659518797
5.80	-657.351667044	-656.598277388	-657.501911033	-656.598374385	-657.659489577
5.90	-657.351643283	-656.598280042	-657.501886235	-656.598377094	-657.659463661
6.00	-657.351622137	-656.598281947	-657.501864231	-656.598379057	-657.659440725
6.10	-657.351603333	-656.598283310	-657.501844717	-656.598380479	-657.659420432
6.20	-657.351586620	-656.598284285	-657.501827413	-656.598381508	-657.659402479
6.30	-657.351571759	-656.598284982	-657.501812065	-656.598382252	-657.659386593
6.40	-657.351558542	-656.598285480	-657.501798439	-656.598382789	-657.659372517
6.50	-657.351546778	-656.598285838	-657.501786331	-656.598383176	-657.659360031
6.60	-657.351536297	-656.598286096	-657.501775559	-656.598383456	-657.659348941
6.70	-657.351526949	-656.598286282	-657.501765963	-656.598383658	-657.659339076
6.80	-657.351518601	-656.598286417	-657.501757403	-656.598383803	-657.659330287
6.90	-657.351511136	-656.598286514	-657.501749753	-656.598383907	-657.659322440
7.00	-657.351504451	-656.598286584	-657.501742910	-656.598383982	-657.659315429
7.10	-657.351498457	-656.598286634	-657.501736778	-656.598384036	-657.659309149
7.20	-657.351493071	-656.598286667	-657.501731274	-656.598384074	-657.659303518
7.30	-657.351488228	-656.598286690	-657.501726327	-656.598384102	-657.659298459
7.40	-657.351483864	-656.598286702	-657.501721873	-656.598384122	-657.659293907
7.50	-657.351479925	-656.598286708	-657.501717858	-656.598384135	-657.659289808
7.60	-657.351476367	-656.598286708	-657.501714232	-656.598384145	-657.659286105
7.70	-657.351473146	-656.598286703	-657.501710954	-656.598384152	-657.659282760
7.80	-657.351470227	-656.598286695	-657.501707985	-656.598384157	-657.659279732
7.90	-657.351467577	-656.598286684	-657.501705293	-656.598384160	-657.659276988
8.00	-657.351465169	-656.598286672	-657.501702849	-656.598384162	-657.659274498
8.10	-657.351462976	-656.598286658	-657.501700626	-656.598384164	-657.659272234
8.20	-657.351460978	-656.598286643	-657.501698602	-656.598384165	-657.659270175
8.30	-657.351459154	-656.598286629	-657.501696757	-656.598384165	-657.659268298
8.40	-657.351457488	-656.598286613	-657.501695073	-656.598384166	-657.659266586
8.50	-657.351455959	-656.598286599	-657.501693532	-656.598384166	-657.659265026
8.60	-657.351454567	-656.598286584	-657.501692123	-656.598384166	-657.659263590
8.70	-657.351453283	-656.598286570	-657.501690831	-656.598384166	-657.659262281
8.80	-657.351452108	-656.598286556	-657.501689645	-656.598384164	-657.659261078
8.90	-657.351451028	-656.598286544	-657.501688556	-656.598384164	-657.659259973
9.00	-657.351450035	-656.598286532	-657.501687554	-656.598384164	-657.659258954
9.10	-657.351449124	-656.598286520	-657.501686631	-656.598384164	-657.659258011
9.20	-657.351448275	-656.598286510	-657.501685781	-656.598384164	-657.659257155
9.30	-657.351447496	-656.598286500	-657.501684996	-656.598384164	-657.659256357
9.40	-657.351446777	-328.299143246	-657.501684271	-656.598384166	-657.659254941
9.50	-657.351446111	-656.598286484	-657.501683600	-656.598384164	-657.659254313

9.60	-657.351445495	-656.598286476	-657.501682980	-656.598384164	-657.659253731
9.70	-657.351444924	-656.598286470	-657.501682405	-656.598384164	-657.659253191
9.80	-657.351444394	-656.598286464	-657.501681872	-656.598384164	-657.659252691
9.90	-657.351443904	-656.598286459	-657.501681378	-656.598384164	-657.659252227
10.00	-657.351443448	-656.598286454	-657.501680919	-656.598384164	-657.659251794
10.10	-657.351443024	-656.598286450	-657.501680493	-656.598384164	-657.659247386

Table S20: Xe-Rn electronic energies (in Hartree) calculated at CCSD(T)/aug-cc-pV4Z (CCSD(T)-4Z), CCSD(T)/aug-cc-pV5Z (CCSD(T)-5Z), and CCSD(T)/CBS levels.

R(Å)	CCSD(T)-4Z	SCF-4Z	CCSD(T)-5Z	SCF-5Z	CBS
3.50	-616.6157693017	-615.8280558450	-616.7073668405	-615.8281689940	-616.8034056100
3.60	-616.6184245007	-615.8316698250	-616.7099108280	-615.8317794060	-616.8058349210
3.70	-616.6203291015	-615.8344049580	-616.7117257921	-615.8345121300	-616.8075571934
3.80	-616.6216755935	-615.8364703090	-616.7130006884	-615.8365759260	-616.8087578465
3.90	-616.6226100883	-615.8380264780	-616.7138784735	-615.8381311640	-616.8095766558
4.00	-616.6232429560	-615.8391965080	-616.7144668488	-615.8393006490	-616.8101186568
4.10	-616.6236571314	-615.8400744090	-616.7148464289	-615.8401782450	-616.8104621115
4.20	-616.6239145895	-615.8407318140	-616.7150772089	-615.8408354720	-616.8106650014
4.30	-616.6240613257	-615.8412231610	-616.7152033834	-615.8413267350	-616.8107696502
4.40	-616.6241312278	-615.8415897150	-616.7152574715	-615.8416932450	-616.8108071712
4.42	-616.6241382962	-615.8416509460	-616.7152618266	-615.8417544720	-616.8108086819
4.43	-616.6241411095	-615.8416802320	-616.7152633321	-615.8417837570	-616.8108088158
4.44	-616.6241434674	-615.8417086660	-616.7152644173	-615.8418121900	-616.8108085663
4.45	-616.6241453875	-615.8417362710	-616.7152650981	-615.8418397950	-616.8108079469
4.46	-616.6241468889	-615.8417630730	-616.7152653886	-615.8418665940	-616.8108069687
4.47	-616.6241479887	-615.8417890930	-616.7152653079	-615.8418926140	-616.8108056492
4.48	-616.6241487038	-615.8418143540	-616.7152648719	-615.8419178740	-616.8108040062
4.49	-616.6241490499	-615.8418388770	-616.7152640935	-615.8419423980	-616.8108020474
4.50	-616.6241490431	-615.8418626850	-616.7152629894	-615.8419662050	-616.8107997926
4.51	-616.6241486991	-615.8418857970	-616.7152615731	-615.8419893170	-616.8107972513
4.52	-616.6241480315	-615.8419082330	-616.7152598600	-615.8420117530	-616.8107944414
4.53	-616.6241470545	-615.8419300130	-616.7152578646	-615.8420335330	-616.8107913774
4.54	-616.6241457833	-615.8419511550	-616.7152555956	-615.8420546760	-616.8107880610
4.55	-616.6241442310	-615.8419716780	-616.7152530739	-615.8420751900	-616.8107845273
4.56	-616.6241424095	-615.8419916000	-616.7152502994	-615.8420951130	-616.8107807523
4.57	-616.6241403321	-615.8420109380	-616.7152472914	-615.8421144520	-616.8107767675
4.60	-616.6241326783	-615.8420656120	-616.7152369756	-615.8421691290	-616.8107636569
4.70	-616.6240949196	-615.8422162220	-616.7151914871	-615.8423197330	-616.8107100619
4.80	-616.6240447892	-615.8423278270	-616.7151350485	-615.8424313530	-616.8106469964
4.90	-616.6239885065	-615.8424104080	-616.7150735227	-615.8425139400	-616.8105799664
5.00	-616.6239302707	-615.8424714290	-616.7150108296	-615.8425749570	-616.8105125988
5.10	-616.6238728551	-615.8425164620	-616.7149495357	-615.8426199750	-616.8104472444
5.20	-616.6238179239	-615.8425496540	-616.7148912414	-615.8426531450	-616.8103854340
5.30	-616.6237664887	-615.8425740890	-616.7148368478	-615.8426775520	-616.8103279521
5.40	-616.6237190398	-615.8425920550	-616.7147867836	-615.8426954930	-616.8102751582
5.50	-616.6236757383	-615.8426052460	-616.7147411915	-615.8427086610	-616.8102271757
5.60	-616.6236365319	-615.8426149150	-616.7146999772	-615.8427183190	-616.8101838609
5.70	-616.6236012353	-615.8426219900	-616.7146629377	-615.8427253930	-616.8101449934
5.80	-616.6235695900	-615.8426271560	-616.7146298019	-615.8427305690	-616.8101102881
5.90	-616.6235413023	-615.8426309190	-616.7146002410	-615.8427343520	-616.8100793802
6.00	-616.6235160675	-615.8426336540	-616.7145739353	-615.8427371150	-616.8100519353
6.10	-616.6234935787	-615.8426356380	-616.7145505443	-615.8427391300	-616.8100275802
6.20	-616.6234735458	-615.8426370720	-616.7145297690	-615.8427406000	-616.8100060058

6.30	-616.6234557174	-615.8426381070	-616.7145113172	-615.8427416700	-616.8099868803
6.40	-616.6234398422	-615.8426388530	-616.7144949170	-615.8427424490	-616.8099699107
6.50	-616.6234257000	-615.8426393900	-616.7144803614	-615.8427430160	-616.8099549044
6.60	-616.6234130919	-615.8426397770	-616.7144673910	-615.8427434280	-616.8099415399
6.70	-616.6234018416	-615.8426400550	-616.7144558410	-615.8427437270	-616.8099296638
6.80	-616.6233917914	-615.8426402550	-616.7144455237	-615.8427439440	-616.8099190566
6.90	-616.6233828131	-615.8426404000	-616.7144363122	-615.8427441010	-616.8099095936
7.00	-616.6233747631	-615.8426405040	-616.7144280707	-615.8427442150	-616.8099011457
7.10	-616.6233675439	-615.8426405800	-616.7144206869	-615.8427442970	-616.8098935857
7.20	-616.6233610600	-615.8426406340	-616.7144140619	-615.8427443570	-616.8098868093
7.30	-616.6233552282	-615.8426406720	-616.7144081079	-615.8427443990	-616.8098807249
7.40	-616.6233499751	-615.8426406980	-616.7144027494	-615.8427444300	-616.8098752531
7.50	-616.6233452363	-615.8426407160	-616.7143979207	-615.8427444520	-616.8098703278
7.60	-616.6233409547	-615.8426407270	-616.7143935622	-615.8427444670	-616.8098658864
7.70	-616.6233370807	-615.8426407320	-616.7143896223	-615.8427444780	-616.8098618739
7.80	-616.6233335704	-615.8426407340	-616.7143860551	-615.8427444860	-616.8098582437
7.90	-616.6233303849	-615.8426407320	-616.7143828215	-615.8427444910	-616.8098549557
8.00	-616.6233274900	-615.8426407270	-616.7143798859	-615.8427444950	-616.8098519723
8.10	-616.6233248556	-615.8426407210	-616.7143772169	-615.8427444980	-616.8098492619
8.20	-616.6233224554	-615.8426407130	-616.7143747873	-615.8427444990	-616.8098467965
8.60	-616.6233147578	-615.8426406760	-616.7143670100	-615.8427445030	-616.8098389124
9.00	-616.6233093210	-615.8426406760	-616.7143615316	-615.8427445030	-616.8098333906
9.40	-616.6233054123	-615.8426406050	-616.7143575997	-615.8427445030	-616.8098293943
9.80	-616.6233025577	-615.8426405790	-616.7143547308	-615.8427445010	-616.8098264971
10.20	-616.6233004429	-615.8426405610	-616.7143526070	-615.8427445000	-616.8098243542
10.60	-616.6232988559	-615.8426405500	-616.7143510142	-615.8427444990	-616.8098227497
11.00	-616.6232976509	-615.8426405430	-616.7143498055	-615.8427444990	-616.8098215331
11.40	-616.6232963452	-615.8426405400	-616.7143488781	-615.8427444980	-616.8098210015
11.80	-616.6232971585	-615.8426405380	-616.7143481592	-615.8427444970	-616.8098186746
12.20	-616.6232954492	-615.8426405370	-616.7143475970	-615.8427444970	-616.8098193152
12.60	-616.6232950053	-615.8426405370	-616.7143471533	-615.8427444970	-616.8098188718
13.00	-616.6232946529	-615.8426405370	-616.7143468004	-615.8427444970	-616.8098185183
13.40	-616.6232943706	-615.8426405370	-616.7143465175	-615.8427444970	-616.8098182349
13.80	-616.6232941426	-615.8426405370	-616.7143462894	-615.8427444970	-616.8098180064
14.20	-616.6232939575	-615.8426405370	-616.7143461040	-615.8427444970	-616.8098178209
14.60	-616.6232938061	-615.8426405370	-616.7143459524	-615.8427444970	-616.8098176692

Table S21: Rn_2 electronic energies (in Hartree) calculated at CCSD(T)/aug-cc-pV4Z (CCSD(T)-4Z), CCSD(T)/aug-cc-pV5Z (CCSD(T)-5Z), and CCSD(T)/CBS levels.

R(Å)	CCSD(T)-4Z	SCF-4Z	CCSD(T)-5Z	SCF-5Z	CBS
3.50	-575.8857722961	-575.0695621520	-576.0759220961	-575.0696834490	-576.2753553818
3.60	-575.8889615042	-575.0738099790	-576.0789778568	-575.0739259790	-576.2782741079
3.70	-575.8912709406	-575.0770416590	-576.0811783182	-575.0771537670	-576.2803624214
3.80	-575.8929216941	-575.0794950710	-576.0827405056	-575.0796045570	-576.2818331599
3.90	-575.8940824193	-575.0813540520	-576.0838298415	-575.0814616500	-576.2828486562
4.00	-575.8948812892	-575.0827596910	-576.0845716740	-575.0828660730	-576.2835313293
4.10	-575.8954152286	-575.0838204610	-576.0850606169	-575.0839260960	-576.2839734825
4.20	-575.8957572166	-575.0846194600	-576.0853675138	-575.0847246510	-576.2842438119
4.30	-575.8959618499	-575.0852201750	-576.0855451309	-575.0853251200	-576.2843932224
4.40	-575.8960697547	-575.0856710050	-576.0856323049	-575.0857758280	-576.2844587146
4.47	-575.8961041574	-575.0859175220	-576.0856550983	-575.0860222810	-576.2844693637
4.48	-575.8961068971	-575.0859488340	-576.0856563471	-575.0860535860	-576.2844690521
4.49	-575.8961091468	-575.0859792510	-576.0856571546	-575.0860839960	-576.2844683505

4.50	-575.8961108808	-575.0860087980	-576.0856575333	-575.0861135370	-576.2844673107
4.51	-575.8961122259	-575.0860374980	-576.0856575003	-575.0861422310	-576.2844658351
4.52	-575.8961131477	-575.0860653770	-576.0856570680	-575.0861701050	-576.2844639849
4.53	-575.8961136581	-575.0860924570	-576.0856562729	-575.0861971790	-576.2844618234
4.54	-575.8961137764	-575.0861187600	-576.0856551216	-575.0862234780	-576.2844593423
4.55	-575.8961135209	-575.0861443090	-576.0856536220	-575.0862490220	-576.2844565404
4.56	-575.8961129054	-575.0861691240	-576.0856517983	-575.0862738340	-576.2844534505
4.57	-575.8961119491	-575.0861932270	-576.0856496616	-575.0862979330	-576.2844500777
4.58	-575.8961106638	-575.0862166370	-576.0856472311	-575.0863213390	-576.2844464479
4.59	-575.8961090595	-575.0862393740	-576.0856445092	-575.0863440730	-576.2844425552
4.60	-575.8961072331	-575.0862614570	-576.0856415166	-575.0863661520	-576.2844383412
4.70	-575.8960745175	-575.0864501350	-576.0855991992	-575.0865548070	-576.2843859629
4.80	-575.8960241323	-575.0865908140	-576.0855411817	-575.0866954780	-576.2843199420
4.90	-575.8959641331	-575.0866955460	-576.0854749092	-575.0868002100	-576.2842470878
5.00	-575.8958999363	-575.0867733980	-576.0854055558	-575.0868780710	-576.2841723191
5.10	-575.8958352920	-575.0868312050	-576.0853364500	-575.0869358760	-576.2840985337
5.20	-575.8957725239	-575.0868740680	-576.0852698229	-575.0869787330	-576.2840278611
5.30	-575.8957130830	-575.0869057940	-576.0852070045	-575.0870104680	-576.2839614941
5.40	-575.8956578137	-575.0869292870	-576.0851486877	-575.0870339380	-576.2838999929
5.50	-575.8956070441	-575.0869466500	-576.0850951796	-575.0870512740	-576.2838436266
5.60	-575.8955608324	-575.0869594690	-576.0850465279	-575.0870640660	-576.2837924300
5.70	-575.8955190903	-575.0869689230	-576.0850025754	-575.0870734930	-576.2837461736
5.80	-575.8954815108	-575.0869758860	-576.0849630625	-575.0870804340	-576.2837046447
5.90	-575.8954478148	-575.0869810070	-576.0849276913	-575.0870855390	-576.2836675249
6.00	-575.8954176774	-575.0869847650	-576.0848961216	-575.0870892900	-576.2836344563
6.10	-575.8953907742	-575.0869875190	-576.0848961216	-575.0870892900	-576.2836642298
6.50	-575.8953093167	-575.0869928330	-576.0847831996	-575.0870974190	-576.2835167144
7.00	-575.8952479707	-575.0869944460	-576.0847199513	-575.0870991240	-576.2834514186
7.50	-575.8952124007	-575.0869947650	-576.0846834990	-575.0870994700	-576.2834140254
8.00	-575.8951910463	-575.0869948250	-576.0846617197	-575.0870995360	-576.2833917969
8.50	-575.8951777586	-575.0869948180	-576.0846482317	-575.0870995480	-576.2833780881
9.00	-575.8951692176	-575.0869947910	-576.0846395965	-575.0870995500	-576.2833693379
10.00	-575.8951597343	-575.0869947420	-576.0846300529	-575.0870995480	-576.2833597047
11.00	-575.8951552223	-575.0869947170	-576.0846255103	-575.0870995440	-576.2833551181
12.00	-575.8951528993	-575.0869947100	-576.0846231783	-574.5119755020	-576.6064536460
13.00	-575.8951516318	-575.0869947080	-576.0846219089	-574.5107452270	-576.6071435337
14.00	-575.8951509047	-575.0869947080	-576.0846211799	-574.5082214470	-576.6085606457
15.00	-575.8951504696	-575.0869947080	-576.0846207443	-574.5082202910	-576.6085608589
16.00	-575.8951501998	-575.0869947080	-576.0846204741	-574.5082201750	-576.6085606536

Table S22: Root-mean-square deviation values (in hartree) obtained in the fitting of the ILJ β parameter using the CCSD(T)/aug-cc-pVQZ, CCSD(T)/aug-cc-pV5Z, and CCSD(T)/CBS electronic energies.

Systems	CCSD(T)/aug-cc-pVQZ	CCSD(T)/aug-cc-pV5Z	CCSD(T)/CBS
He_2	9.76x10^{-8}	1.18x10^{-7}	1.05 x10^{-7}
He-Ne	2.15x10^{-7}	9.14x10^{-8}	3.74 x10^{-7}
He-Ar	1.50x10^{-7}	2.59x10^{-7}	2.89 x10^{-7}
He-Kr	1.29x10^{-7}	1.52x10^{-7}	5.37 x10^{-7}
He-Xe	1.25x10^{-7}	2.77x10^{-7}	4.46 x10^{-6}
He-Rn	1.58x10^{-7}	2.78x10^{-7}	5.28 x10^{-7}
Ne_2	2.12x10^{-7}	3.58x10^{-7}	1.36 x10^{-6}
Ne-Ar	4.70x10^{-7}	5.35x10^{-7}	8.91 x10^{-7}
Ne-Kr	3.69x10^{-7}	5.67x10^{-7}	2.45 x10^{-6}
Ne-Xe	5.39x10^{-7}	7.67x10^{-7}	1.10 x10^{-5}
Ne-Rn	5.09x10^{-7}	9.21x10^{-7}	1.06 x10^{-6}
Ar_2	1.21x10^{-6}	1.82x10^{-6}	5.00 x10^{-6}
Ar-Kr	1.40x10^{-6}	2.54x10^{-6}	3.97 x10^{-5}
Ar-Xe	1.86x10^{-6}	2.98x10^{-6}	5.48 x10^{-6}
Ar-Rn	2.41x10^{-6}	2.93x10^{-6}	2.93 x10^{-6}
Kr-kr	2.18x10^{-6}	2.63x10^{-6}	7.23 x10^{-6}
Kr-Xe	2.60x10^{-6}	4.62x10^{-6}	5.23 x10^{-6}
Kr-Rn	3.99x10^{-6}	5.15x10^{-6}	4.64 x10^{-6}
Xe_2	4.22x10^{-6}	4.90x10^{-6}	5.95 x10^{-6}
Xe-Rn	4.23x10^{-6}	6.19x10^{-6}	7.08 x10^{-6}
Rn_2	7.34x10^{-6}	5.94x10^{-6}	9.02 x10^{-6}

Table S23: Ng-Ng molecule reduced mass values (atomic units).

Systems	Reduced mass	Systems	Reduced mass
He_2	3647.5969	Ar_2	36410.3449
He-Ne	6088.5874	Ar-Kr	49312.9086
He-Ar	6631.8115	Ar-Xe	55823.4657
He-Kr	6963.6764	Ar-Rn	61715.2761
He-Xe	7080.2848	Kr_2	76378.9653
He-Rn	7167.0672	Kr-Xe	93217.8628
Ne_2	18392.0184	Kr-Rn	110896.8626
Ne-Ar	24439.0823	Xe_2	119581.3872
Ne-Kr	29645.4311	Xe-Rn	150323.1481
Ne-Xe	31880.6811	Rn_2	202340.4570
Ne-Rn	33719.0928	–	–

Table S24: Ne_2, Ar_2, Kr_2, Xe_2, and Rn_2 rovibrational energies (RE). The CBS acronym stands for the RE calculated with DVR method and a ILJ PEC with R_e and D_e determined at CCSD(T)/CBS level and β fitted from CCSD(T)/CBS electronic energies. The β9-Exp acronym stands for RE determined by DVR method and a ILJ PEC with R_e, D_e, β (equal 9) experimental values. v and J represent the vibrational and rotational quantum numbers, respectively. The RE values are given in cm^{-1}.

		Ne_2	Ar_2		Kr_2		Xe_2		Rn_2		
v	J	CBS	β9-Exp	CBS	β9-Exp	CBS	β9-Exp	CBS	β9-Exp	CB	β9-Exp
0		12.23	12.33	15.02	14.61	11.07	11.40	9.88	10.02	7.11	-
1		26.55	26.27	40.83	39.91	31.64	32.56	28.78	29.17	22.38	-
2		-	-	61.29	60.21	50.18	51.56	46.54	47.14	37.05	-
3		-	-	76.70	75.71	66.71	68.45	63.18	63.94	51.11	-
4		-	-	87.50	86.77	81.27	83.27	78.71	79.59	64.58	-
5		-	-	94.32	93.90	93.90	96.08	93.14	94.10	77.44	-
6		-	-	98.12	97.93	104.65	106.94	106.49	107.48	89.71	-
7		-	-	-	-	113.61	115.94	118.76	119.75	101.39	-
8		-	-	-	-	120.87	123.20	129.97	130.94	112.48	-
9		-	-	-	-	126.55	128.85	140.15	141.06	122.99	-
10		-	-	-	-	130.80	133.04	149.32	150.14	132.93	-
11		-	-	-	-	133.80	135.99	157.51	158.22	142.29	-
12		-	-	-	-	135.93	138.07	164.74	165.33	151.08	-
13		-	-	-	-	-	-	171.06	171.51	159.31	-
14		-	-	-	-	-	-	176.50	176.80	166.99	-
15		-	-	-	-	-	-	181.12	181.27	174.12	-
16		-	-	-	-	-	-	184.96	184.96	180.72	-
17		-	-	-	-	-	-	188.10	187.95	186.79	-
18		-	-	-	-	-	-	190.59	190.30	192.36	-
19		-	-	-	-	-	-	192.54	192.14	197.42	-
20		-	-	-	-	-	-	194.33	193.91	202.00	-
21		-	-	-	-	-	-	-	-	206.11	-
22		-	-	-	-	-	-	-	-	209.78	-
23		-	-	-	-	-	-	-	-	213.01	-
24		-	-	-	-	-	-	-	-	215.84	-
25		-	-	-	-	-	-	-	-	218.29	-
26		-	-	-	-	-	-	-	-	220.38	-
27	0	-	-	-	-	-	-	-	-	222.13	-
28		-	-	-	-	-	-	-	-	223.58	-
29		-	-	-	-	-	-	-	-	224.75	-
30		-	-	-	-	-	-	-	-	225.67	-
31		-	-	-	-	-	-	-	-	226.37	-
32		-	-	-	-	-	-	-	-	226.87	-
33		-	-	-	-	-	-	-	-	227.22	-
34		-	-	-	-	-	-	-	-	227.45	-
35		-	-	-	-	-	-	-	-	227.69	-
36		-	-	-	-	-	-	-	-	227.98	-
37		-	-	-	-	-	-	-	-	228.32	-
38		-	-	-	-	-	-	-	-	228.72	-
39		-	-	-	-	-	-	-	-	229.15	-
40		-	-	-	-	-	-	-	-	229.63	-
41		-	-	-	-	-	-	-	-	230.15	-
42		-	-	-	-	-	-	-	-	230.70	-
43		-	-	-	-	-	-	-	-	231.29	-
44		-	-	-	-	-	-	-	-	231.91	-
45		-	-	-	-	-	-	-	-	232.56	-

46	-	-	-	-	-	-	-	-	233.25	-
47	-	-	-	-	-	-	-	-	233.97	-
48	-	-	-	-	-	-	-	-	234.72	-
49	-	-	-	-	-	-	-	-	235.50	-
50	-	-	-	-	-	-	-	-	236.30	-
51	-	-	-	-	-	-	-	-	237.14	-
52	-	-	-	-	-	-	-	-	238.01	-
53	-	-	-	-	-	-	-	-	238.91	-
54	-	-	-	-	-	-	-	-	239.83	-
55	-	-	-	-	-	-	-	-	240.78	-
56	-	-	-	-	-	-	-	-	241.76	-
57	-	-	-	-	-	-	-	-	242.77	-
58	-	-	-	-	-	-	-	-	243.80	-
0	13.33	13.43	15.13	14.72	11.11	11.45	9.91	10.05	7.13	-
1	27.29	27.00	40.94	40.02	31.69	32.60	28.80	29.20	22.40	-
2	-	-	61.39	60.31	50.22	51.60	46.57	47.16	37.06	-
3	-	-	76.78	75.80	66.75	68.49	63.21	63.97	51.13	-
4	-	-	87.57	86.85	81.31	83.31	78.74	79.61	64.59	-
5	-	-	94.38	93.96	93.93	96.11	93.17	94.12	77.45	-
6	-	-	98.17	97.97	104.69	106.97	106.51	107.50	89.72	-
7	-	-	-	-	113.65	115.98	118.78	119.77	101.40	-
8	-	-	-	-	120.90	123.23	129.99	130.96	112.50	-
9	-	-	-	-	126.58	128.87	140.17	141.08	123.01	-
10	-	-	-	-	130.82	133.07	149.34	150.16	132.94	-
11	-	-	-	-	133.82	136.01	157.53	158.24	142.30	-
12	-	-	-	-	135.95	138.09	164.76	165.35	151.09	-
13	-	-	-	-	-	-	171.07	171.52	159.32	-
14	-	-	-	-	-	-	176.51	176.82	167.00	-
15	-	-	-	-	-	-	181.13	181.28	174.13	-
16	-	-	-	-	-	-	184.98	184.97	180.73	-
17	-	-	-	-	-	-	188.11	187.96	186.80	-
18	-	-	-	-	-	-	190.60	190.31	192.37	-
19	-	-	-	-	-	-	192.55	192.15	197.43	-
20	-	-	-	-	-	-	194.34	193.92	202.01	-
21	-	-	-	-	-	-	-	-	206.12	-
22	-	-	-	-	-	-	-	-	209.79	-
23	-	-	-	-	-	-	-	-	213.02	-
24	-	-	-	-	-	-	-	-	215.85	-
25	-	-	-	-	-	-	-	-	218.30	-
26	-	-	-	-	-	-	-	-	220.38	-
27 1	-	-	-	-	-	-	-	-	222.14	-
28	-	-	-	-	-	-	-	-	223.58	-
29	-	-	-	-	-	-	-	-	224.75	-
30	-	-	-	-	-	-	-	-	225.67	-
31	-	-	-	-	-	-	-	-	226.37	-
32	-	-	-	-	-	-	-	-	226.88	-
33	-	-	-	-	-	-	-	-	227.22	-
34	-	-	-	-	-	-	-	-	227.46	-
35	-	-	-	-	-	-	-	-	227.69	-
36	-	-	-	-	-	-	-	-	227.98	-
37	-	-	-	-	-	-	-	-	228.33	-
38	-	-	-	-	-	-	-	-	228.72	-
39	-	-	-	-	-	-	-	-	229.16	-

40	-	-	-	-	-	-	-	-	229.64	-
41	-	-	-	-	-	-	-	-	230.15	-
42	-	-	-	-	-	-	-	-	230.70	-
43	-	-	-	-	-	-	-	-	231.29	-
44	-	-	-	-	-	-	-	-	231.91	-
45	-	-	-	-	-	-	-	-	232.57	-
46	-	-	-	-	-	-	-	-	233.25	-
47	-	-	-	-	-	-	-	-	233.97	-
48	-	-	-	-	-	-	-	-	234.72	-
49	-	-	-	-	-	-	-	-	235.50	-
50	-	-	-	-	-	-	-	-	236.31	-
51	-	-	-	-	-	-	-	-	237.15	-
52	-	-	-	-	-	-	-	-	238.01	-
53	-	-	-	-	-	-	-	-	238.91	-
54	-	-	-	-	-	-	-	-	239.83	-
55	-	-	-	-	-	-	-	-	240.78	-
56	-	-	-	-	-	-	-	-	241.76	-
57	-	-	-	-	-	-	-	-	242.77	-
58	-	-	-	-	-	-	-	-	243.80	-

Table S25: He-Ne, He-Ar, He-Kr, He-Xe, He-Rn, Ne-Ar, Ne-Kr, Ne-Xe, Ne-Rn, Ar-Kr, Ar-Xe, Ar-Rn, Kr-Xe, Kr-Rn, and Xe-Rn rovibrational energies (RE). The CBS acronym stands for the RE calculated with DVR method and a ILJ PEC with R_e and D_e determined at CCSD(T)/CBS level and β fitted from CCSD(T)/CBS electronic energies. The $\beta 9$ acronym stands for RE determined by DVR method and a ILJ PEC with R_e and D_e obtained at CCSD(T)/CBS level and β equal to 9 (Experimental value). v and J represent the vibrational and rotational quantum numbers, respectively. The RE values are given in cm^{-1}.

		He-Ne		He-Ar		He-Kr		He-Xe	
v	J	CBS	$\beta 9$	CBS	$\beta 9$	CBS	$\beta 9$	CBS	$\beta 9$
0	0	12.48	12.16	13.65	13.67	12.95	13.09	13.16	12.14
0	1	13.20	12.88	15.75	15.78	13.47	13.61	14.69	13.73
		He-Rn		Ne-Ar		Ne-Kr		Ne-Xe	
v	J	CBS	$\beta 9$	CBS	$\beta 9$	CBS	$\beta 9$	CBS	$\beta 9$
0		10.59	-	12.82	12.46	11.67	11.42	10.87	10.62
1		18.72	-	31.66	30.88	29.85	29.28	28.33	27.71
2	0	20.35	-	42.31	41.41	41.66	40.92	40.43	39.60
3		-	-	46.55	45.66	47.97	47.15	47.77	46.80
4		-	-	-	-	50.58	-	51.47	50.42
0		10.99	-	13.51	13.14	12.19	11.94	10.99	10.74
1		18.88	-	32.23	31.44	30.30	29.73	28.44	27.82
2	1	20.49	-	42.73	41.82	42.03	41.29	40.52	39.69
3		-	-	46.80	45.91	48.24	47.42	47.84	46.88
4		-	-	-	-	50.77	-	51.53	50.47
		Ne-Rn		Ar-Kr		Ar-Xe		Ar-Rn	
v	J	CBS	$\beta 9$	CBS	$\beta 9$	CBS	$\beta 9$	CBS	$\beta 9$-Exp
0		3.32	-	13.52	13.12	12.38	12.52	22.54	-
1		20.54	-	37.68	36.69	35.10	35.42	42.75	-
2		33.00	-	58.10	56.78	55.18	55.51	60.57	-
3		41.04	-	74.95	73.51	72.64	72.87	76.05	-
4		45.35	-	88.39	87.00	87.56	87.56	89.26	-
5		46.99	-	98.67	97.45	100.01	99.69	100.29	-
6		47.62	-	106.09	105.09	110.08	109.39	109.24	-
7		48.50	-	111.08	110.28	117.94	116.84	116.26	-
8		49.69	-	114.69	113.98	123.76	122.27	121.52	-
9		51.13	-	-	-	127.82	126.01	125.24	-
10		52.83	-	-	-	131.00	129.14	127.65	-
11	0	-	-	-	-	-	-	129.04	-
12		-	-	-	-	-	-	129.75	-
13		-	-	-	-	-	-	130.43	-
14		-	-	-	-	-	-	131.34	-
15		-	-	-	-	-	-	132.45	-
16		-	-	-	-	-	-	133.73	-
17		-	-	-	-	-	-	135.19	-
18		-	-	-	-	-	-	136.79	-
19		-	-	-	-	-	-	138.54	-
20		-	-	-	-	-	-	140.44	-
0		3.32	-	13.60	13.20	12.44	12.59	22.59	-
1		20.54	-	37.75	36.76	35.17	35.48	42.80	-
2		33.00	-	58.17	56.85	55.24	55.57	60.62	-
3		41.04	-	75.01	73.58	72.70	72.92	76.10	-

4		45.35	-	88.45	87.06	87.61	87.61	89.31	-
5		46.99	-	98.72	97.50	100.05	99.73	100.33	-
6		47.62	-	106.14	105.13	110.13	109.43	109.28	-
7		48.50	-	111.11	110.32	117.97	116.88	116.29	-
8		49.69	-	114.73	114.01	123.79	122.30	121.55	-
9		51.13	-	-	-	127.85	126.04	125.26	-
10		52.83	-	-	-	131.02	129.16	127.67	-
11	1	-	-	-	-	-	-	129.05	-
12		-	-	-	-	-	-	129.76	-
13		-	-	-	-	-	-	130.44	-
14		-	-	-	-	-	-	131.35	-
15		-	-	-	-	-	-	132.46	-
16		-	-	-	-	-	-	133.75	-
17		-	-	-	-	-	-	135.20	-
18		-	-	-	-	-	-	136.81	-
19		-	-	-	-	-	-	138.56	-
20		-	-	-	-	-	-	140.46	-
		Kr-Xe		Kr-Rn		Xe-Rn		-	
v	J	CBS	$\beta 9$	CBS	$\beta 9$	CBS	$\beta 9$	CBS	$\beta 9$
0		10.48	10.63	15.98	-	10.85	-	-	-
1		30.25	30.66	33.65	-	27.50	-	-	-
2		48.48	49.07	50.05	-	43.31	-	-	-
3		65.16	65.89	65.18	-	58.28	-	-	-
4		80.33	81.13	79.06	-	72.42	-	-	-
5		94.01	94.84	91.72	-	85.72	-	-	-
6		106.22	107.04	103.18	-	98.20	-	-	-
7		117.00	117.78	113.45	-	109.87	-	-	-
8		126.40	127.09	122.58	-	120.74	-	-	-
9		134.46	135.04	130.59	-	130.81	-	-	-
10		141.24	141.70	137.53	-	140.09	-	-	-
11		146.83	147.15	143.45	-	148.61	-	-	-
12		151.30	151.48	148.41	-	156.37	-	-	-
13		154.77	154.81	152.48	-	163.40	-	-	-
14		157.34	157.26	155.73	-	169.71	-	-	-
15		159.23	159.06	158.23	-	175.33	-	-	-
16		160.99	-	160.09	-	180.28	-	-	-
17		-	-	161.39	-	184.60	-	-	-
18		-	-	162.24	-	188.31	-	-	-
19		-	-	162.72	-	191.47	-	-	-
20		-	-	162.98	-	194.09	-	-	-
21		-	-	163.28	-	196.24	-	-	-
22		-	-	163.66	-	197.95	-	-	-
23		-	-	164.12	-	199.27	-	-	-
24	0	-	-	164.65	-	200.25	-	-	-
25		-	-	165.24	-	200.93	-	-	-
26		-	-	165.90	-	201.38	-	-	-
27		-	-	166.61	-	201.65	-	-	-
28		-	-	167.38	-	201.91	-	-	-
29		-	-	168.20	-	202.25	-	-	-
30		-	-	169.08	-	202.65	-	-	-
31		-	-	170.00	-	203.11	-	-	-
32		-	-	170.98	-	203.63	-	-	-
33		-	-	172.00	-	204.19	-	-	-
34		-	-	173.08	-	204.81	-	-	-

35		-	-	174.20	-	205.47	-	-	-
36		-	-	175.37	-	206.17	-	-	-
37		-	-	-	-	206.92			
38		-	-	-	-	207.71	-	-	-
39		-	-	-	-	208.53	-	-	-
40		-	-	-	-	209.40	-	-	-
41		-	-	-	-	210.31	-	-	-
42		-	-	-	-	211.26	-	-	-
43		-	-	-	-	212.25	-	-	-
44		-	-	-	-	213.27	-	-	-
45		-	-	-	-	214.33	-	-	-
46		-	-	-	-	215.42	-	-	-
47		-	-	-	-	216.55	-	-	-
						-	-	-	-
0		10.61	10.76	16.01	-	10.87	-	-	-
1		30.38	30.78	33.68	-	27.52	-	-	-
2		48.60	49.19	50.07	-	43.33	-	-	-
3		65.28	66.00	65.21	-	58.30	-	-	-
4		80.44	81.25	79.09	-	72.43	-	-	-
5		94.12	94.95	91.75	-	85.74	-	-	-
6		106.32	107.15	103.20	-	98.22	-	-	-
7		117.10	117.87	113.48	-	109.89	-	-	-
8		126.49	127.18	122.60	-	120.75	-	-	-
9		134.55	135.13	130.61	-	130.82	-	-	-
10		141.33	141.78	137.55	-	140.11	-	-	-
11		146.90	147.22	143.47	-	148.62	-	-	-
12		151.37	151.55	148.43	-	156.38	-	-	-
13		154.83	154.87	152.49	-	163.41	-	-	-
14		157.40	157.31	155.74	-	169.72	-	-	-
15		159.27	159.11	158.25	-	175.34	-	-	-
16		161.04	-	160.10	-	180.29	-	-	-
17		-	-	161.40	-	184.61	-	-	-
18		-	-	162.24	-	188.32	-	-	-
19		-	-	162.72	-	191.48	-	-	-
20		-	-	162.99	-	194.10	-	-	-
21		-	-	163.28	-	196.25	-	-	-
22		-	-	163.66	-	197.96	-	-	-
23		-	-	164.13	-	199.28	-	-	-
24	1	-	-	164.66	-	200.25	-	-	-
25		-	-	165.25	-	200.94	-	-	-
26		-	-	165.90	-	201.38	-	-	-
27		-	-	166.62	-	201.66	-	-	-
28		-	-	167.39	-	201.92	-	-	-
29		-	-	168.21	-	202.25	-	-	-
30		-	-	169.08	-	202.65	-	-	-
31		-	-	170.01	-	203.11	-	-	-
32		-	-	170.98	-	203.63	-	-	-
33		-	-	172.01	-	204.20	-	-	-
34		-	-	173.08	-	204.81	-	-	-
35		-	-	174.20	-	205.47	-	-	-
36		-	-	175.37	-	206.17	-	-	-
37		-	-		-	206.92	-	-	-
38		-	-		-	207.71	-	-	-
39		-	-		-	208.54	-	-	-

40	-	-	-	209.41	-	-	-
41	-	-	-	210.32	-	-	-
42	-	-	-	211.26	-	-	-
43	-	-	-	212.25	-	-	-
44	-	-	-	213.27	-	-	-
45	-	-	-	214.33	-	-	-
46	-	-	-	215.43	-	-	-
47	-	-	-	216.56	-	-	-
